# Genetically determined blood pressure, antihypertensive drug classes, and frailty: A Mendelian randomization study

**DOI:** 10.1111/acel.14173

**Published:** 2024-05-09

**Authors:** Zhenhuang Zhuang, Yueying Li, Yimin Zhao, Ninghao Huang, Wenxiu Wang, Wendi Xiao, Jie Du, Xue Dong, Zimin Song, Jinzhu Jia, Zhonghua Liu, Robert Clarke, Lu Qi, Tao Huang

**Affiliations:** ^1^ Department of Epidemiology and Biostatistics, School of Public Health Peking University Beijing China; ^2^ National Institute for Nutrition and Health Chinese Center for Diseases Control and Prevention Beijing China; ^3^ Department of Biostatistics, School of Public Health Peking University Beijing China; ^4^ Department of Biostatistics Columbia University New York New York USA; ^5^ Clinical Trial Service Unit and Epidemiological Studies Unit (CTSU), Nuffield Department of Population Health University of Oxford Oxford UK; ^6^ Department of Epidemiology, School of Public Health and Tropical Medicine Tulane University New Orleans Louisiana USA; ^7^ Department of Nutrition Harvard T.H. Chan School of Public Health Boston Massachusetts USA; ^8^ Key Laboratory of Epidemiology of Major Diseases (Peking University) Ministry of Education Beijing China; ^9^ Center for Intelligent Public Health, Academy for Artificial Intelligence Peking University Beijing China

**Keywords:** antihypertensive medication, causality, frailty, Mendelian randomization

## Abstract

Observational studies have suggested that the use of antihypertensive drugs was associated with the risk of frailty; however, these findings may be biased by confounding and reverse causality. This study aimed to explore the effect of genetically predicted lifelong lowering blood pressure (BP) through different antihypertensive medications on frailty. One‐sample Mendelian randomization (MR) and summary data‐based MR (SMR) were applied. We utilized two kinds of genetic instruments to proxy the antihypertensive medications, including genetic variants within or nearby drugs target genes associated with systolic/diastolic BP, and expression level of the corresponding gene. Among 298,618 UK Biobank participants, one‐sample MR analysis observed that genetically proxied BB use (relative risk ratios, 0.76; 95% CI, 0.65–0.90; *p* = 0.001) and CCB use (0.83; 0.72–0.95; *p* = 0.007), equivalent to a 10‐mm Hg reduction in systolic BP, was significantly associated with lower risk of pre‐frailty. In addition, although not statistically significant, the effect directions of systolic BP through ACEi variants (0.72; 0.39–1.33; *p* = 0.296) or thiazides variants (0.74; 0.53–1.03; *p* = 0.072) on pre‐frailty were also protective. Similar results were obtained in analyses for diastolic BP. SMR of expression in artery showed that decreased expression level of KCNH2, a target gene of BBs, was associated with lower frailty index (beta −0.02, *p* = 2.87 × 10^−4^). This MR analysis found evidence that the use of BBs and CCBs was potentially associated with reduced frailty risk in the general population, and identified KCNH2 as a promising target for further clinical trials to prevent manifestations of frailty.

## Graphical abstract

This study provides genetic evidence that the use of beta‐blockers and calcium channel blockers are potentially associated with reduced frailty risk in the general population, with a particular emphasis on KCNH2, a beta‐blocker‐related gene, as a promising target for further clinical trials to preventing manifestations of frailty. Taken together, these findings call for further investigation on the preventive strategies of frailty targeting blood pressure control.

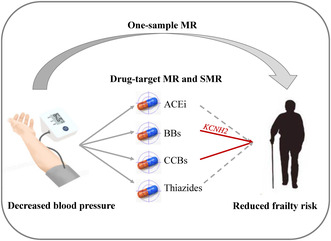

**Abbreviations**
ACEi: angiotensin converting enzyme inhibitor
ARB: angiotensin II receptor blockers
BB: beta‐blocker
BP: blood pressure
CCB: calcium channel blocker
CI: confidence interval
eQTLs: expression quantitative trait locus
FI: frailty index
GRS: genetic risk score
GWAS: genome‐wide association study
HEIDI: heterogeneity in dependent instruments
IV: instrumental variable
LD: linkage disequilibrium
MAF: minor allele frequency
MR: Mendelian randomization
PC: principal component
RRR: relative risk ratio
SMR: summary data‐based Mendelian randomization
SNP: single‐nucleotide polymorphism
THIN: The Health Improvement Network

## 1 BACKGROUND

The prevalence of hypertension, especially systolic hypertension has increased progressively worldwide in recent decades, reflecting rapid population aging (Benetos et al., 2019). First‐line antihypertensive medications (e.g., angiotensin converting enzyme inhibitors [ACEi], angiotensin II receptor blockers [ARB], beta‐blockers [BB], calcium channel blockers [CCB], and thiazides) are not only widely used to lower mean levels of blood pressure (BP) in individuals diagnosed with elevated BP (Wright & Musini, 2009), but also ideal drug‐repurposing candidates to improve aging‐related disorders (Georgakis et al., 2020; Gill et al., 2019; Ou et al., 2021). Recent systematic reviews have reported the significant positive association between hypertension and frailty, which reflects overall health status that accompany elevated BP in mid‐life and late middle‐age. However, the causal relevance of antihypertensive medications for frailty remains uncertain in the general population (Qipo et al., 2022; Vetrano et al., 2018). Moreover, antihypertensive medications have proven potential effect on overall health, including blood, adipose tissue, heart, kidney, and brain disorders (Mancia et al., 1993; van Middelaar et al., 2018; Zanchetti et al., 2015), but their relevance for frailty and possible mediation by lowering BP or by other mechanisms has not been reliably established.

Mendelian randomization (MR) is a novel method using genetic variants as instrumental variables (IVs) to evaluate the causal relevance of risk factors in a nonexperimental (observational) setting (Davies et al., 2018). BP lowering can be achieved through several approved antihypertensive medications with multiple pharmacological targets, such as ACE, KCNH2, SLC12A1, and CACNA1C, which have different mechanisms of action and may affect overall health to different degrees (encapsulating BP‐lowering effects along with any other on‐ and off‐target physiological effects). Therefore, the effects of drug action can be anticipated together by the genetic effects in the genes of their several well‐known protein targets, as has previously been applied to antihypertensive medications (Chauquet et al., 2021). Moreover, novel molecular phenotypes such as gene expression data (GTEx Consortium, 2013) and new methods such as summary data‐based MR (SMR) (Zhu et al., 2016) have been used widely to identify tissue‐specific causal genes for complex diseases, which can be used to investigate the biological mechanisms involved in the action of antihypertensive medications on frailty.

The aims of this report were to (i) assess the causal relevance of antihypertensive medications on the risk of frailty; (ii) compare differences in different antihypertensive medications on frailty, and (iii) expression of drug targets in relevant tissues for prevention of frailty.

## 2 METHODS

### 2.1 Study population

The UK Biobank is a multicenter prospective cohort study of over 500,000 participants aged 40–69 years who were enrolled from 22 assessment centers across the United Kingdom between 2006 and 2010. Detailed information of the study design and cohort profile have been described previously (Sudlow et al., 2015). Of 487,298 participants who were genotyped in the UK Biobank, we included 377,995 white, British‐descent participants with valid baseline measurements of BP, frailty indicator, and relevant covariates. The present analyses were limited to homogenous ethnic background to minimize risk of population stratification due to different allele frequencies in multiple ethnic groups which can cause bias in genetic analyses. We further excluded those with a non‐Caucasian genetic ethnic grouping (*n* = 39,807), missingness rate higher than 1% (*n* = 38,857), mismatch between reported and genetic sex (*n* = 211), recommended genomic analysis exclusions (*n* = 219), sex chromosome aneuploidy (*n* = 651), and genetic kinship to other participants (*n* = 31). A total of 298,618 white British individuals were included in the final analysis (Figure S1).

The UK Biobank study was approved by the National Information Governance Board for Health and Social Care in England and Wales, and the Community Health Index Advisory Group in Scotland and the North West Multicenter Research Ethics Committee. All participants provided written informed consent.

### 2.2 Exposure and outcomes

After a 2‐min rest, BP values were automatically measured twice in a seated position using an appropriate cuff and an Omron HEM‐7015IT digital BP monitor. Measurements involved both systolic and diastolic BP were considered. We calculated mean systolic and diastolic BP from two automated BP measurements. For a few individuals with single automated BP measurements (*n* = 8959), we used that single measurements. A manual sphygmomanometer was used if the standard automated device could not be employed (*n* = 16,834). According to previously published studies (Kanki et al., 2023; Paz et al., 2016; Siedlinski et al., 2020; Tobin et al., 2005; Warren et al., 2017), we added 15 mmHg to systolic BP and 10 mm Hg to diastolic BP for individuals reported to be taking BP‐lowering medication, respectively.

A standard frailty index (FI) has been developed and validated as published previously (Williams et al., 2019), involving 49 items including 11 types of deficits (sensory, cranial, mental well‐being, infirmity, cardiometabolic, respiratory, musculoskeletal, immunological, cancer, pain, and gastrointestinal) indicating poor health status across a variety of physiological and mental domains, symptoms, diagnosed diseases, and disabilities, which were assessed using questionnaire and interview at baseline. A FI was calculated as the sum of deficits accrued by an individual divided by the total number of deficits included in the FI. In this study, we classified participants as being non‐frail (FI <0.12), pre‐frail (FI 0.12–0.24), or frail (FI >0.24). These cutoffs were developed and validated using the ResearchOne and The Health Improvement Network (THIN) cohorts (Clegg et al., 2016), which have been applied in the UK Biobank (Petermann‐Rocha et al., 2020). Relevant details about the FI composition are provided in Table S1.

### 2.3 Covariates

Age at baseline was derived from date of birth and baseline assessment. Standing height and weight measurements recorded at baseline were used to calculate body mass index (calculated as weight in kilograms divided by the square of the height in meters). We defined smoking status based on self‐reported information as never, previous, and current smokers. Self‐reported alcohol intake frequency was categorized as never, occasionally, 1–3 times a month, 1–2 times a week, 3–4 times a week, and daily. Area‐based socioeconomic status (deprivation) was obtained from the postcode of residence using the Townsend score. Further details of these measurements can be seen in the UK Biobank online protocol (http://www.ukbiobank.ac.uk).

### 2.4 Genotype data

The single‐nucleotide polymorphisms (SNPs) used as instrumental variables in this study were extracted from the UK Biobank imputation data set. Genotype data were imputed with IMPUTE4 using the Haplotype Reference Consortium and the UK10K + 1000 Genomes panel to identify approximately 96 million variants for 487,298 participants (Bycroft et al., 2018).

### 2.5 Selection of genetic instruments

The SNPs selected from a genome‐wide association study (GWAS) meta‐analysis that included 757,601 individuals of European ancestry drawn from UK Biobank (*N* = 458,577) and the International Consortium of BP (*N* = 299,024) database (together with their published single‐variant effect sizes on systolic and diastolic BP, respectively) were genome‐wide significant, independent, and common (*p* < 5 × 10^−8^; *R* ^2^ < 0.001; minor allele frequency [MAF] >1%) (Evangelou et al., 2018). We selected SNPs as proxies for the BP‐lowering effects of common antihypertensive medications: ACEI, ARB (no proxy available), BB, CCB, and thiazides on the basis of new consensus guidelines (Wright et al., 2018). We identified the genes encoding pharmacologic targets related to BP lowering for common antihypertensive medications in DrugBank (https://www.drugbank.ca/) (Wishart et al., 2018) and screened the genomic SNPs corresponding to these genes in GeneCards (https://www.genecards.org/) (Table S2) (Fishilevich et al., 2017). From all the identified SNPs in each gene region (±100 kb), we only considered SNPs that were significantly associated with systolic/diastolic BP (*p* < 5 × 10^−8^) and clumped to a linkage disequilibrium (LD) threshold of *R* ^2^ < 0.4 using the 1000G European reference panel as candidate proxies for each drug class. This relatively lenient LD correlation threshold allows for an increase in proportion of variance explained and thus in statistical power (Burgess et al., 2016, 2018; Georgakis et al., 2020; Ou et al., 2021). All these SNPs were used to constructed weighted genetic risk scores (GRS) for BP using the following formula: (β_1_ × SNP_1_ + β_2_ × SNP_2_ + ⋯ + β_n_ × SNP_n_) × number of selected SNPs/sum of regression coefficients, where β_i_ was the regression coefficient associated with SNP_i_ and obtained from the previous GWAS meta‐analysis (Tables S3–S6). Overall, the weighted GRS explained 3.6% and 3.8% of the variance in systolic and diastolic BP, respectively, corresponding to F statistics of 1001 and 1067. An unweighted GRS was also developed to further assess the impact between the weighting method and the estimates.

Secondly, to further clarify the potential mechanism linking antihypertensive medications and frailty, we also used available expression quantitative trait locus (eQTLs) for drugs target genes as the proxy of exposure to each BP‐lowering drug. The eQTLs summary‐level data were obtained from GTEx Consortium V8 (https://gtexportal.org/), which are presented in detail in Table S7. We identified common (MAF >1%) eQTLs SNPs significantly (*p* < 5.0 × 10^−8^) associated with the expression of antihypertensive medication related target genes in adipose tissue, blood, brain, heart, nerve, and kidney, which have been proven to be potentially affected by antihypertensive medications. Only cis‐eQTLs were included to generate genetic instruments in this study, which were defined as eQTLs within 1 Mb on either side of the encoded gene.

### 2.6 Statistical analysis

**FIGURE 1:**
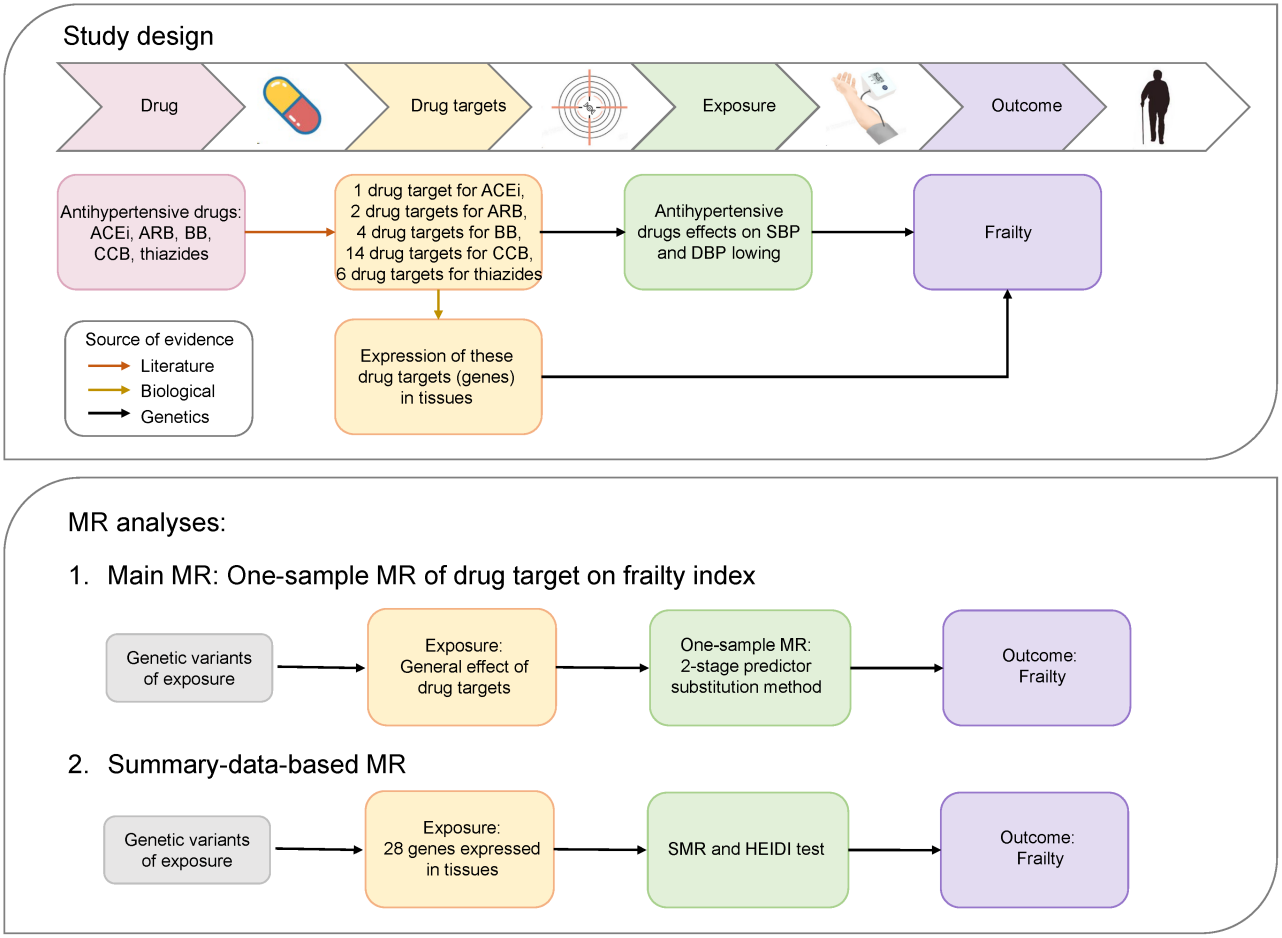
Diagram of the study design. This MR study aims to identify the causal relationships between antihypertensive drugs, and drug targets, general metformin effects (exposure), expression of drug target genes (exposures), and frailty (outcomes). Three levels of evidence were used to construct the causal atlas, including literature and biological and genetic evidence. ACEi, angiotensin‐converting enzyme inhibitors; BB, β‐blockers; CCB, calcium channel blockers; MR, Mendelian randomization.

Figure 1 outlines the design of this study, which included the two main analyses conducted in this study: (1) the one‐sample MR analysis estimating the effect of antihypertensive medication targets on frailty risk; and (2) the SMR analysis estimating the effects of genes encoding antihypertensive medication targets in these tissues on frailty risk. Given the rationale that genetic code is fixed at conception, MR approaches can take account of the constraint of observational studies and evaluates lifelong differences in risk factors, and thereby used to identify potential causal relevance between measured BP (e.g., systolic BP) and frailty.

This one‐sample MR approach involved three assumptions: (1) the GRS is reliably associated with the BP measurements, (2) the GRS have no association with any confounding factors, and (3) the risk of frailty is influenced only through the exposure of interest, instead of other pathways (i.e., no pleiotropic effects) (Davey Smith & Hemani, 2014; Walker et al., 2017). We performed one‐sample MR analysis using an multi‐adjusted, two‐stage predictor substitution method that used the GRS as an instrument variable. In this analysis, a serial regression method was conducted by executing two regression models in succession. In the first stage, BP measurements was regressed on the GRS as an instrumental variable using a multiple linear regression model. The predicted probability and residuals from this model were saved for the next stage. In the second stage, outcomes were regressed on the predicted probability from the first stage, which served as an independent variable (this predicted value was a proxy for unconfounded estimate of measured BP), in multinomial logistic regression models to investigate the association of BP levels with frailty status, defined as non‐frail, pre‐frail, and frail. We included age, sex, assessment centers, smoking status, alcohol intake, BMI, deprivation, genetic array, and the first 10 principal components (PCs) as covariates. All MR associations were presented as relative risk ratios (RRR) and 95% confidence intervals (95% CI) of frailty status per genetically predicted 10‐mm Hg increment in systolic BP and 5‐mm Hg in diastolic BP, in order to be comparable to the results of randomized controlled trials (Ettehad et al., 2016; Georgakis et al., 2020). The associations are considered statistically significant after the correction for multiple testing for two BP measurements [*p* < 0.025 (0.05/2)]. A *p* value above 0.025 but below 0.05 was considered suggestive of evidence for a potential association. All these analyses were implemented in Stata/SE 14/0.0 (StataCorp, College Station, TX).

For the SMR analysis, we used summary‐level data from GWAS and eQTL studies to investigate the causal associations between expression levels of antihypertensive medication‐related genes and the FI (Figure 1) (Atkins et al., 2021; Zhu et al., 2016). Only gene expression probes with at least one cis‐eQTL at *p* < 5 × 10^−8^ were included. We selected the top associated cis‐eQTL with the lowest *p* value to separate from other cis‐eQTLs in a gene. For each probe, we used the effect estimates of the top cis‐eQTL from the eQTL study (βzx) and the GWAS (bzy) to test for the association between a trait and probe (bxy). We performed SMR analysis using the default parameters recommended by the developers of SMR software version 1.03 (https://cnsgenomics.com/software/smr/#Overview). Bonferroni correction was used to account for multiple testing; therefore, a genome‐wide significance level was suggested for *p* < 0.003 (15 genes and 1 outcome) and a suggestive significance level of 0.003 ≤ *p* < 0.05.

### 2.7 Sensitivity analyses

We performed several sensitivity analyses to test the robustness of our findings. For one‐sample MR method, we employed more stringent pairwise LD thresholds (*R* ^2^ < 0.1 and *R* ^2^ < 0.01, respectively) in the selection of independent SNPs for constructing the IVs of drug targets to remove nominally correlated estimates among the genetic variants. To address the possibility that the BP levels may be associated with frailty risk due to underlying associations between BP IVs and cardiometabolic traits (e.g., diabetes, cardiovascular diseases, dyslipidemia, etc.), a modified FI excluding the eight items related to cardiometabolic health was also used for MR analyses (Table S1). To minimize the influence of the missing data on our results, we repeated the primary analysis using a sample if they did not have missing data on 10 or more frailty items. To increase the generalizability of the study findings, we included participants from other ethnic/genetic groups. In addition, we also applied Fried frailty phenotype as a complementary indicator of physical frailty (Hanlon et al., 2018). For SMR method, the heterogeneity in dependent instruments (HEIDI) test was used to test whether observed association between gene expression and FI was likely due to a pleiotropic scenario, which was indicated by a HEIDI test (*p* < 0.01) (Zhu et al., 2016). In the HEIDI test, we excluded SNPs in LDs with top cis‐eQTLs with *r* ^2^ > 0.9, as SNPs in almost perfect LDs with top cis‐eQTLs are not able to provide information for the HEIDI test. To avoid weak instrumental variables, we also removed SNPs in the cis‐eQTL region with *p*_eQTL_ > 1.6 × 10^−3^ (Zhu et al., 2016).

### 2.8 Patient and public involvement

No participants were involved in setting the research question or the outcome measures, nor were they involved in the design or implementation of the study. No participants were asked to advise on interpretation or writing up of results. There are no plans to disseminate the results of the research to study participants or other relevant patient groups.

## 3 RESULTS

### 3.1 Descriptive statistics and observational findings

Selected baseline characteristics of the 298,618 unrelated individuals of white British ancestry who contributed to the main analyses are presented by frailty status in Table 1. Among these, 192,952 (64.6%) participants had the criteria for non‐frailty, 95,907 (32.1%) had pre‐frailty, and 9759 (3.3%) had frailty. Compared with non‐frail participants, frail participants were older and more likely to be female, socioeconomically deprived, current smokers, obese, and report lower alcohol intake (Table 1). Compared with non‐frail participants, mean BP measurements were higher in both frail and in pre‐frail participants, particularly for systolic BP (Table 1). GRSs composed of 436/441 independent genetic variants were strongly and positively associated with higher levels of both systolic and diastolic BP (*p* < 1 × 10^−4^, see Figure S2). In addition, there was little evidence of heterogeneity in the effect of systolic/diastolic BP, suggesting that the associations between the variants and BP might be similar across the population.

**TABLE 1:** Distribution of demographic and clinical characteristics, overall and across participants' frailty status.

	Frailty status		
	Non‐frail	Pre‐frail	Frail
	(FI <0.12)	(FI 0.12–0.24)	(FI >0.24)
*n* (%)	192,952 (64.6)	95,907 (32.1)	9759 (3.3)
Age, mean (SD), years	56 (8.1)	58 (7.7)	59 (7.1)
Women, *n* (%)	100,315 (52.0)	53,506 (55.8)	5540 (56.8)
Systolic BP, mean (SD), mm Hg	138.9 (19.6)	141.3 (20.0)	140.9 (19.8)
Diastolic BP, mean (SD), mm Hg	82.9 (10.7)	83.9 (11.1)	83.5 (11.7)
Fifths of deprivation, *n* (%)			
First (least deprived)	46,192 (23.9)	18,482 (19.3)	1034 (10.6)
Second	44,149 (22.9)	19,240 (20.1)	1325 (13.6)
Third	40,966 (21.2)	19,647 (20.5)	1587 (16.3)
Fourth	36,060 (18.7)	19,452 (20.3)	2123 (21.8)
Fifth (most deprived)	25,585 (13.3)	19,086 (19.9)	3690 (37.8)
Smoking status, *n* (%)			
Never	113,679 (58.9)	46,705 (48.7)	3592 (36.8)
Previous	63,564 (32.9)	37,880 (39.5)	4202 (43.1)
Current	15,709 (8.1)	11,322 (11.8)	1965 (20.1)
Frequency of alcohol intake, *n* (%)			
Daily	44,071 (22.8)	19,534 (19)	1408 (14.4)
1–4 times/week	103,977 (53.9)	44,884 (46.8)	3387 (34.7)
1–3 times/month	19,846 (10.3)	11,502 (12.0)	1249 (12.8)
Occasionally/never	25,058 (13.0)	19,987 (20.84)	3715 (38.1)
Categories of body mass index, *n* (%)			
<18 kg/m^2^	478 (0.3)	293 (0.3)	44 (0.5)
18–25 kg/m^2^	72,401 (37.5)	25,038 (26.1)	1457 (14.9)
25–30 kg/m^2^	85,102 (44.1)	40,108 (41.8)	3212 (32.9)
≥30 kg/m^2^	34,971 (18.1)	30,468 (31.8)	5046 (51.7)

*Note*: Results shown as mean (SD), unless otherwise indicated.

Abbreviation: FI, frailty index.

### 3.2 Mendelian randomization findings

**FIGURE 2:**
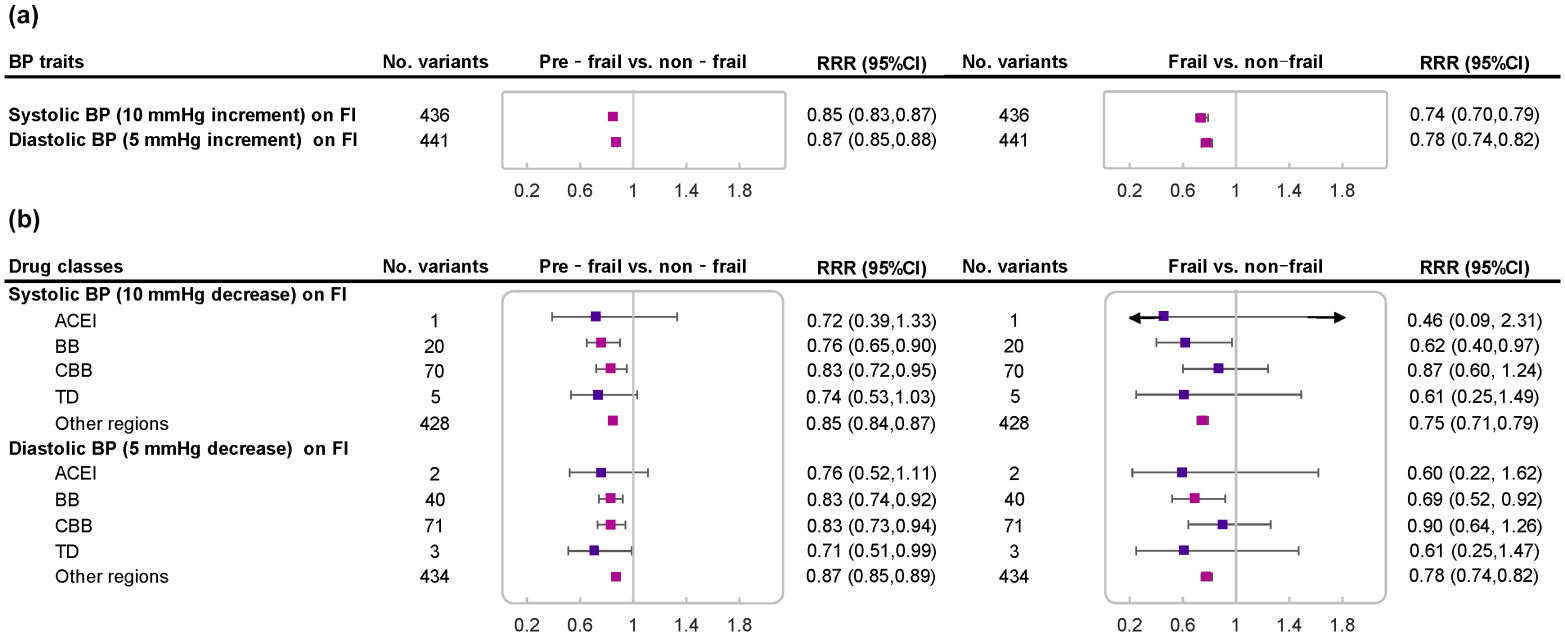
Estimated causal effects of lower systolic BP (10 mmHg decrease) and diastolic BP (5 mmHg decrease) through antihypertensive drugs on frailty. RRRs (95% CIs) were obtained from one‐sample Mendelian randomization analysis. Associations reflect the estimated causal effect on frailty risk per 10 mm Hg decrease in systolic BP or per 5 mm Hg decrease in diastolic BP. ACEi, angiotensin‐converting enzyme inhibitors; BB, β‐blockers; BP, blood pressure; CCB, calcium channel blockers; CI, confidence interval.

We first selected BP‐lowering variants in genes encoding drug targets as proxies for the effects of antihypertensive medications, as outlined in Figure 1, and examined their effects on frailty in MR analyses. A genetically predicted 10‐mm Hg reduction in systolic BP through variants in genes encoding targets of BBs (RRR, 0.76; 95% CI, 0.65–0.90; *p* = 0.001) and CCBs (0.83; 0.72–0.95; *p* = 0.007), was significantly associated with lower risk of pre‐frailty (Figure 2). Although not statistically significant, the effect direction of systolic BP through ACEi variants (0.72; 0.39–1.33; *p* = 0.296) or thiazides variants (0.74; 0.53–1.03; *p* = 0.072) on pre‐frailty was also protective. In analyses for diastolic BP, we also found a 5‐mm Hg lower diastolic BP through BB variants to be associated with significantly lower risks of pre‐frailty (0.83; 0.74–0.92; *p* = 0.001) and frailty (0.69; 0.52–0.92; *p* = 0.012), which was the strongest effect among the four drugs. CCB variants showed a protective effect on pre‐frailty risk (0.83; 0.73–0.94; *p* = 0.004). However, ACEI variants (0.76; 0.52–1.11; *p* = 0.151) or thiazides variants (0.71; 0.51–0.99; *p* = 0.044) demonstrated a consistent null association with pre‐frailty.

In addition, we examined the relationship between genetically determined BP and the risk of frailty. Figure 2 shows that each genetically predicted, 10‐mm Hg lower systolic BP was associated with a lower risk reduction of pre‐frailty (0.85; 0.83–0.87; *p* < 0.001) and frailty (0.74; 0.70–0.79; *p* < 0.001), implying that the effects of antihypertensive medication targets on frailty were likely to be mediated by their effects on BP. There were similar associations between genetically predicted 5‐mm Hg lower diastolic BP and frailty status (pre‐frailty, 0.87; 0.85–0.88; *p* < 0.001; frailty, 0.78; 0.74–0.82; *p* < 0.001). Using all BP‐related SNPs outside these regions across the genome also demonstrated strong support for causal relevance of lower BP on risk of frailty.

The associations were broadly consistent when excluding younger participants <60 years old, with the BP‐lowering effects on frailty instrumented by SNPs in the region of the CCB targets remarkably enhanced (0.48; 0.29–0.78; *p* = 0.003 for systolic BP; 0.51; 0.32–0.38; *p* = 0.004 for diastolic BP) (Table 2). However, smaller effects were observed for the modified FI excluding eight items related to cardiometabolic deficits: among antihypertensive medication targets, only BB variants were associated with a 12% lower risk of frailty (0.88; 0.80–0.98; *p* = 0.023) (Table 2, Table S1).

**TABLE 2:** Causal estimates for the FI effect of lifelong lowering of BP indicated by the constructed IVs.

IV set	Pre‐frail versus non‐frail		Frail versus non‐frail	
	RRR (95% CI)	*p*	RRR (95% CI)	*p*
Frailty in participants 60 years (*n* = 134,421)				
Systolic BP	0.82 (0.79, 0.84)	<0.001**	0.73 (0.67, 0.78)	<0.001**
ACEi	1.00 (0.41, 2.44)	0.992	0.38 (0.04, 3.42)	0.389
BB	0.73 (0.57, 0.93)	0.010**	0.68 (0.38, 1.24)	0.208
CCB	0.76 (0.63, 0.93)	0.008**	0.48 (0.29, 0.78)	0.003**
Thiazides	0.90 (0.56, 1.47)	0.682	0.55 (0.17, 1.83)	0.330
Diastolic BP	0.84 (0.82, 0.87)	<0.001**	0.76 (0.71, 0.81)	<0.001**
ACEi	0.93 (0.54, 1.59)	0.781	0.65 (0.17, 2.48)	0.527
BB	0.78 (0.67, 0.91)	0.002**	0.70 (0.47, 1.03)	0.208
CCB	0.73 (0.61, 0.88)	0.001**	0.51 (0.32, 0.80)	0.004**
Thiazides	0.92 (0.57, 1.45)	0.734	0.56 (0.17, 1.85)	0.345
Modified FI excluding cardiometabolic deficit items (*n* = 298,618)				
Systolic BP	0.87 (0.85, 0.89)	<0.001**	0.82 (0.79, 0.86)	<0.001**
ACEi	1.10 (0.60, 2.00)	0.764	0.42 (0.11, 1.55)	0.194
BB	0.84 (0.72, 0.99)	0.039*	0.80 (0.56, 1.14)	0.221
CCB	0.88 (0.77, 1.00)	0.055	0.84 (0.63, 1.12)	0.239
Thiazides	0.71 (0.51, 0.99)	0.041*	0.52 (0.25, 1.05)	0.069
Diastolic BP	0.88 (0.87, 0.90)	<0.001**	0.84 (0.81, 0.88)	<0.001**
ACEi	1.01 (0.70, 1.45)	0.964	0.65 (0.29, 1.44)	0.288
BB	0.88 (0.80, 0.98)	0.023**	0.83 (0.66, 1.05)	0.120
CCB	0.87 (0.77, 0.99)	0.028*	0.87 (0.67, 1.14)	0.120
Thiazides	0.73 (0.53, 1.01)	0.060	0.46 (0.23, 0.92)	0.029*

Abbreviations: ACEi, angiotensin‐converting enzyme inhibitors; BB, β‐blockers; CCB, calcium channel blockers; FI, frailty index; RRR, relative risk ratios.

** Statistical significance level after the correction for multiple testing.

* Suggestive significance level.

**FIGURE 3:**
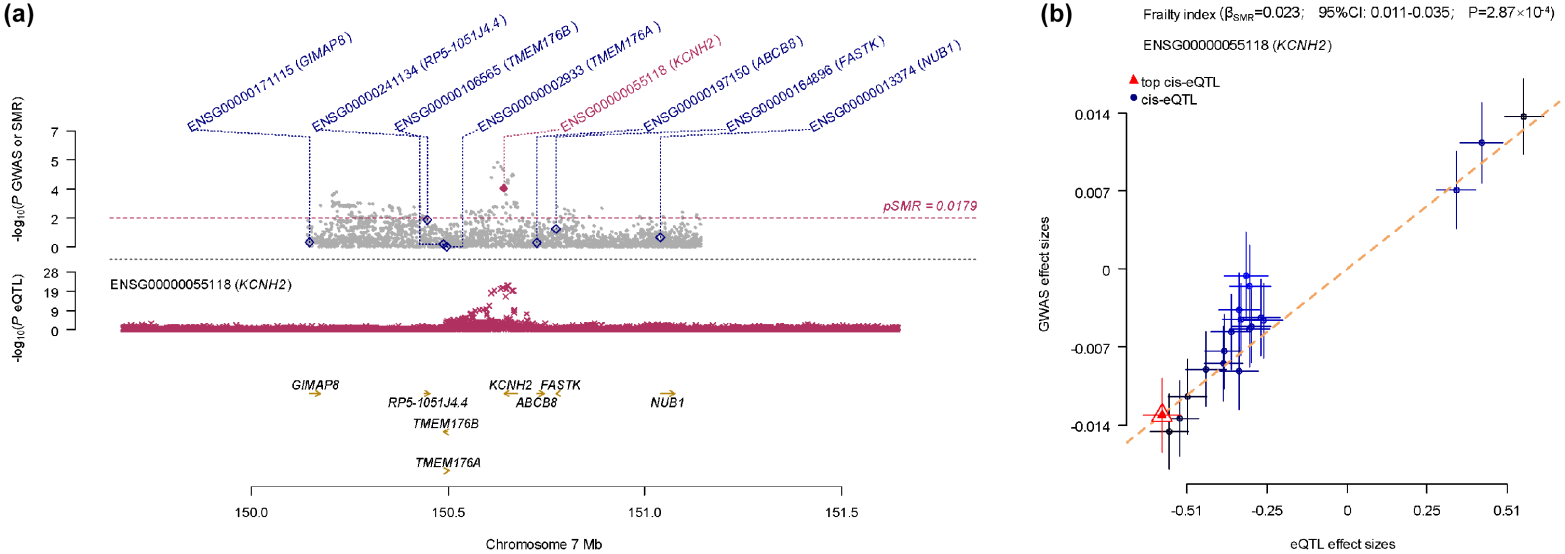
SMR effects of KCNH2 expression in artery aorta on frailty. (a) SMR locus plot of KCNH2 expression in the cis‐acting KCNH2 region. (b) SMR effect plot in the KCNH2 region with effect sizes from GWAS against those from eQTL study. Result of other genes are listed in Table S8. One unit refers to 1 SD of the expression level change of KCNH2. RRR, relative risk ratios.

We further investigated whether the expression of antihypertensive medication‐related genes may have a causal relevance for frailty. As shown in Figure 3 and Table S7, decreased expression level of a BB‐related gene, KCNH2, was associated with lower FI in the aorta (β −0.02; *p* = 2.87 × 10^−4^) and the tibial artery (β −0.05; *p* = 6.23 × 10^−4^). There was no evidence of the causal associations between antihypertensive medication targets and the risk of frailty in any other tissues.

### 3.3 Sensitivity analyses

For one‐sample MR, the F statistic from the first‐stage regression of the measured systolic BP (*F* = 1001) and diastolic BP (*F* = 1067) on the GRS supported that our GRS was a strong instrument, indicating low risk of weak instrument bias (Tables S3 and S4). First, estimates using the unweighted GRS also yielded similar estimates to those in main analyses (Table S8). Second, when more stringent pairwise LD thresholds (0.1 and 0.01) were used for selecting independent genetic variants to construct the IVs for antihypertensive medication targets, the corresponding estimates remained concordant with the overall results (Tables S9 and S10). Third, including those having missing data on less than 10 frailty items obtained similar estimates (Table S11). Fourth, we observed that the results remained unchanged when adding participants from other ethnic/genetic groups (Table S12). Fifth, systolic BP (0.94; 0.89–0.99; *p* = 0.023) and diastolic BP (0.95; 0.90–0.99; *p* = 0.021) was associated with frailty, while no causal effect of lifelong lowering of BP was observed through any antihypertensive drug classes on frailty status (Table S13). For SMR analysis, HEIDI test suggested that all observed associations were not due to pleiotropy (*p* > 0.01) (Table S7).

## 4 DISCUSSION

Using an MR approach in the UK Biobank, we profiled the BP‐lowering effects of antihypertensive medications on frailty measured by FI—analogue to overall health profile—accumulated by mid‐life and late middle‐age, and demonstrated reliable evidence that the on‐target effects of BB and CBB may result in substantial differences to the frailty risk of such users. The SMR analysis provided support for a causal role for the expression level of an BB‐related gene, KCNH2, on FI, with this effect likely to be localized in artery. Collectively, our findings may help with understanding of the drug effect and mechanisms when further extensively used in clinical practice.

Frailty has been common in individuals with hypertension (Liu et al., 2020; Vetrano et al., 2018). A recent systematic review and meta‐analysis have reported that the pooled prevalence of hypertension in frail individuals was 72% and the pooled prevalence of frailty in individuals with hypertension was 14% (Vetrano et al., 2018). However, there are relatively few studies on the relationship between antihypertensive medications treatments and frailty. In observational studies, adherence to antihypertensive medications treatment and adherence to a healthy lifestyle were associated with a lower risk of all‐cause mortality among adults with hypertension (Lu et al., 2022). Results from large‐scale trials have suggested that BP lowering with antihypertensive medications have protective effects on cardiovascular, renal, and cognitive outcomes (Hughes et al., 2020; Xie et al., 2016). It is worth noting that a meta‐analysis of 147 randomized trials have highlighted the importance of lowering BP in everyone over a certain age, indicating that antihypertensive medications could be offered to people with all levels of BP (Law et al., 2009). To address the key gap in the current literature evidence, we used MR approaches to support the causal relevance to lower risk of frailty, which was indicated by a 49‐item FI. Additionally, population‐based studies have suggested that the association of BP with the Fried frailty phenotype varied depending on characteristics such as age, physical and cognitive function, and the use of antihypertensive medications (Kabayama et al., 2020; Rouch et al., 2022). Consistent with observational studies, we observed that the causal effects of systolic BP and diastolic BP with the Fried frailty phenotype were significant (Kabayama et al., 2020; Rouch et al., 2022). However, the BP‐lowing effect of antihypertensive medications was not causally linked with frailty. Although both frailty indicators reflect a higher risk for negative outcomes, the frailty phenotype is a physically mediated pre‐disability syndrome which is defined narrowly (Cesari et al., 2013). The findings of this study using 49‐item FI focused on the accumulation of health defects for the whole organism and provide novel evidence to the effect of BBs and CCBs for frailty prevention in the general population, which would also promote KCNH2 repurposing as a potential frailty prevention strategy for future trial design.

In drug‐specific models, which to some extent mimic the effects of long‐term exposure of individuals to the modulation of antihypertensive medication targets, BBs and CCBs were associated with lower risks of frailty. Given the analogy of an MR analysis and a randomized clinical trial, our drug‐specific IV findings suggested that both BBs and CCBs reduce frailty risk, albeit to variable extent (Emdin et al., 2017). Nevertheless, these hypotheses require corroboration by randomized trial evidence. Results of previous observational findings along these lines have been inconsistent. Feringa et al. reported that BBs were associated with a lower risk of long‐term mortality in 2420 individuals with peripheral arterial disease (Feringa et al., 2006). A prospective cohort study of 3716 patients with end‐stage renal disease found that the risk of all‐cause mortality and cardiovascular mortality during follow‐up period were 21% and 26% lower among CCB users than nonusers, respectively (Kestenbaum et al., 2002). In contrast, an observational study in 344 patients with end‐stage liver disease failed to observe associations between BB use and physical frailty (Kuo et al., 2018). Therefore, further investigations on this issue are warranted.

Sotalol, a class III antiarrhythmic agent, is known to prolong action‐potential duration of cardiomyocytes by blocking the IKr current (Shinnawi et al., 2019). Previous analyses have suggested a possible role for sotalol in the prophylactic treatment of patients with a different mutation (p.T618I) in the KCNH2 gene, which inhibits steady currents of mutant channels effectively to prevent sudden cardiac death (Anttonen et al., 2007; Sun et al., 2011). In this study, we also found that inhibition of expression of an BB‐related gene, KCNH2, in artery was associated with a lower risk of frailty and better overall health. Previous studies have reported this gene to be associated with short QT syndrome, and higher risk of adverse cardiometabolic outcomes (Giustetto et al., 2011; Sun et al., 2011). However, the findings should be explained with caution, since the goals, target values, and choice of antihypertensive medications treatment for frail older adults are still disputed.

Frailty has become a high‐priority theme in cardiovascular medicine due to the aging and the increasingly complex nature of patients suffering from multiple cardiovascular conditions. This study highlights the importance of BP management during the aging process, especially since the association with the frailty may be not confined to cardiovascular pathologies, which is supported by our finding that genetically predicted BP exposure was also associated with the FI even after excluding cardiometabolic‐deficit items. The mechanisms underlying these observations are unknown but may include inflammation, oxidative stress, and endothelial dysfunction, are common to the development of hypertension and frailty (Buford, 2016). In addition, Atkins et al. reported that individuals aged 60–69 years with more benign cardiovascular risk factor profiles, including BP, have substantially lower incident risk of geriatric conditions and frailty than younger participants, which were consistent with the more pronounced association of lower BP with frailty risk in those aged >60 years in this study (Atkins et al., 2019). We thus inferred that optimizing BP levels may have cumulative and more impactful benefits in later life, substantially reducing the burden of frailty.

The main strength of this study includes being the very first study to mimic the long‐term effect of antihypertensive medication targets on frailty risk. We used large datasets to guarantee sufficient statistical power for the analyses and applied multiple sensitivity analyses to test the efficiency of genetic instruments and the assumptions of MR study. In addition, we used genetic proxies for well‐known antihypertensive medication targets that have been validated previously to examine the causal effects of drug targets and/or genes related to drugs' action rather than the direct effects of such drugs (e.g., BB use) on frailty risk. This MR design also enable us to address uncontrolled confounding and reverse causality that may have affected previous observational studies.

Our study findings should be interpreted in light of their limitations. Firstly, MR examines the lifelong effects of genetically predicted BP, which might mimic but differ from the effect of a clinical intervention for BP lowering on frailty. Thus, our MR results only suggested that the BP‐lowering effect of BBs and CCBs should be tested in clinical trials for prevention of frailty. Secondly, our findings represent the average linear causal effects across the general population, while at least two issues related to antihypertensive medications are still undefined: (1) the BP threshold at which antihypertensive medications should be initiated and (2) the BP goals of the therapeutic intervention. Given the rapid development of novel methods (e.g., nonlinear MR), we will evaluate the shape of causal effect in future researches. Thirdly, the biological characteristics of antihypertensive medications is still uncertain. Since limited antihypertensive medication targets (genes) were considered, we were unable to exclude possibility that this study missed drug targets that are still under study or are difficult to identify using existing genetic tools. For instance, we selected only a single genetic proxy for ACEI that might not offer sufficient statistical power to obtain causal estimates. Since the number of instrumental variables is orders of magnitudes higher in the BB and CCB analysis with correspondingly smaller confidence intervals than for ACEi or thiazides, the effect of BB and CCB on frailty may be due to any reduction in BP. Therefore, the causality should be interpreted with great caution. In the future, larger GWAS datasets might identify more significant variants and might thus offer deeper insights into differential effects between different classes of antihypertensive medications. Fourthly, the selection of the genetic instruments in the one‐sample MR analysis was based on a meta‐GWAS that included other cohorts, which may have potential impact on the MR results. Fifthly, the MR results based on the GRS is unable to be comparable with other MR methods multiple genetic instruments, such as inverse variance weighted, weighted median, or weighted mode methods. Further, two‐sample MR analysis using multiple genetic instruments are warranted. Finally, the adjustment of BP values for those reported taking antihypertensive drugs may not be entirely accurate, which have an influence on the analysis results to some extent. Moreover, in this study, among 76,252 participants who were diagnosed with hypertension, only 28,008 participants reported to be taking antihypertensive medications. This may reflect that a large proportion of hypertensive patients have not received treatment, or be due to underreporting of antihypertensive medications.

## 5 CONCLUSION

This MR study examined the BP‐lowering effects via antihypertensive medications on frailty risk using genetic variants, and found potential evidence that BBs and CCBs might be promising strategies for the prevention of frailty in the general population. We also identified a BB‐related gene, KCNH2, as a key mediator in the artery. Taken together, our results provide guidance for future clinical trials design of antihypertensive medications, and call for further investigation on the preventive strategies of frailty targeting BP control.

## AUTHOR CONTRIBUTIONS

TH and LQ designed the research. ZZ and TH had full access to all the data in this study and took responsibility for the integrity of the data and the accuracy of the data analysis. ZZ wrote the article and performed the data analysis. All authors contributed to the statistical analysis, critically reviewed the article during the writing process, and approved the final version to be published. ZZ and TH are the guarantors for this study.

## FUNDING INFORMATION

The study was supported by grants from the National Natural Science Foundation of China (82173499).

## CONFLICT OF INTEREST STATEMENT

All authors declare no support from companies for the submitted work; no relationships with companies that might have an interest in the submitted work in the previous 3 years; no spouses, partners, or children have financial relationships that may be relevant to the submitted work; and no nonfinancial interests that may be relevant to the submitted work.

## Supporting information


Appendix S1

## Data Availability

This research was conducted using the UK Biobank Resource under Application Number 44430. Details of how to access the UK Biobank data and details of the data release schedule are available from www.ukbiobank.ac.uk/.
