# Supplementary material for: Genetically determined blood pressure, antihypertensive drug classes, and frailty: A Mendelian randomization study
Source: Aging Cell. 2024 May 9;23(7):e14173. doi: 10.1111/acel.14173 (PMC11258474; doi:10.1111/acel.14173)
Supplement: Supplementary file 1 — Appendix S1 [file ACEL-23-e14173-s001.docx]

**Contents**

**Supplementary Table 1.** Questionnaire items from the baseline UK Biobank assessment used to compose the FI.

**Supplementary Table 2.** Genetic regions used to identify instruments for each antihypertensive drug.

**Supplementary Table 3.** SNPs related to systolic BP to creat genetic risk score in one-sample Mendelian randomization analysis (n=436).

**Supplementary Table 4.** SNPs related to diastolic BP to creat genetic risk score in one-sample Mendelian randomization analysis (n=441).

**Supplementary Table 5.** SNPs related to systolic BP in genes that are the target of antihypertensive drugs in one-sample Mendelian randomization analysis.

**Supplementary Table 6.** SNPs related to diastolic BP in genes that are the target of antihypertensive drugs in one-sample Mendelian randomization analysis.

**Supplementary Table 7.** SMR estimates of antihypertension drug target genes on FI.

**Supplementary Table 8.** Causal associations between BP and frailty status using unweighted genetic risk score as IVs.

**Supplementary Table 9.** Causal effect of life-long lowering of BP through different antihypertensive drug classes on frailty status indicated by the constructed IVs (r2<0.1).

**Supplementary Table 10.** Causal effect of life-long lowering of BP through different antihypertensive drug classes on frailty status indicated by the constructed IVs (r2<0.01).

**Supplementary Table 11.** Causal associations between BP and frailty status among participants with missing data on less than 10 FI items.

**Supplementary Table 12.** Causal associations between BP and frailty status among participants from multiple ethnic/genetic groups.

**Supplementary Table 13.** Causal associations between BP and frailty status indicated by Fried frailty phenotype.

**Supplementary Figure 1.** Study design schematic for initial exclusion criteria and genetic data quality control.

**Supplementary Figure 2.** Results of the MR analyses of blood pressure on FI using the Steiger filtered set of SNPs.

**Supplementary Table 1. Questionnaire items from the baseline UK Biobank assessment used to compose the FI.**

| **Type of deficit** | **Item** | **Trait** | **Categories** | **Coding in FI item** |
| --- | --- | --- | --- | --- |
| *Sensory* | 1 | Glaucoma * | no,yes | Categorised 0/1 |
|  | 2 | Cataracts * | no,yes | Categorised 0/1 |
|  | 3 | Hearing difficulty | no, yes, completely deaf | Categorised 0/1 (combined yes/deaf groups as 1) |
| *Cranial* | 4 | Migraine * | no,yes | Categorised 0/1 |
|  | 5 | Dental problems | ulcers, painful gums, bleeding gums, loose teeth, toothache, dentures | Categorised 0/1 for none vs. any |
| *Mental wellbeing* | 6 | Self-rated health | excellent, good, fair, poor | 0 – excellent; 0.25 – good; 0.5 - fair; 1 – poor |
|  | 7 | Fatigue: frequency of tiredness / lethargy in last two weeks | not at all, several days, more than half, nearly every day | 0, 0.25, 0.5, 1, respectively |
|  | 8 | Sleep: experience of sleeplessness/insomnia | never/rarely, sometimes, usually | Categorised 0, 0.5, 1, respectively |
|  | 9 | Depressed feelings: frequency in last two weeks | not at all, several days, more than half, nearly every day | 0 – not at all, 0.5 – several days, 0.75 - more than half, 1 – nearly every day |
|  | 10 | Self-described nervous personality | no, yes | Categorised 0/1 |
|  | 11 | Severe anxiety/ panic attacks * | no, yes | Categorised 0/1 |
|  | 12 | Common to feel loneliness | no, yes | Categorised 0/1 |
|  | 13 | Sense of misery (ever/never) | no, yes | Categorised 0/1 |
| *Infirmity* | 14 | Infirmity: long-standing illness or disability | no, yes | Categorised 0/1 |
|  | 15 | Falls in last year | categorical: no falls, one fall, more than one | 0, 0.5, 1, respectively |
|  | 16 | Fractures/broken bones in last five years | no, yes | Categorised 0/1 |
| *Cardiometabolic* | 17 | Diabetes * | no, yes | Categorised 0/1 |
|  | 18 | Myocardial infarction * | no, yes | Categorised 0/1 |
|  | 19 | Angina * | no, yes | Categorised 0/1 |
|  | 20 | Stroke * | no, yes | Categorised 0/1 |
|  | 21 | High blood pressure * | no, yes | Categorised 0/1 |
|  | 22 | Hypothyroidism * | no, yes | Categorised 0/1 |
|  | 23 | Deep-vein thrombosis * | no, yes | Categorised 0/1 |
|  | 24 | High cholesterol * | no, yes | Categorised 0/1 |
| *Respiratory* | 25 | Breathing: wheeze in last year | no, yes | Categorised 0/1 |
|  | 26 | Pneumonia * | no, yes | Categorised 0/1 |
|  | 27 | Chronic bronchitis/emphysema * | no, yes | Categorised 0/1 |
|  | 28 | Asthma * | no, yes | Categorised 0/1 |
| *Musculoskeletal* | 29 | Rheumatoid arthritis * | no, yes | Categorised 0/1 |
|  | 30 | Osteoarthritis * | no, yes | Categorised 0/1 |
|  | 31 | Gout * | no, yes | Categorised 0/1 |
|  | 32 | Osteoporosis * | no, yes | Categorised 0/1 |
| *Immunological* | 33 | Hayfever, allergic rhinitis or eczema * | no, yes | Categorised 0/1 |
|  | 34 | Psoriasis * | no, yes | Categorised 0/1 |
| *Cancer* | 35 | Any cancer diagnosis * | no, yes | Categorised 0/1 |
|  | 36 | Multiple cancers diagnosed (number reported) | Range from 0 to 6 | 0 - no cancer or single cancer, 1 - multiple cancers |
| *Pain* | 37 | Chest pain | no, yes | Categorised 0/1 |
|  | 38 | Head and/or neck pain | no, yes (combining responses to pain in head and neck/shoulders) | Categorised 0/1 |
|  | 39 | Back pain | no, yes | Categorised 0/1 |
|  | 40 | Stomach/abdominal pain | no, yes | Categorised 0/1 |
|  | 41 | Hip pain | no, yes | Categorised 0/1 |
|  | 42 | Knee pain | no, yes | Categorised 0/1 |
|  | 43 | Whole-body pain | no, yes | Categorised 0/1 |
|  | 44 | Facial pain | no, yes | Categorised 0/1 |
|  | 45 | Sciatica * | no, yes | Categorised 0/1 |
| *Gastrointestinal* | 46 | Gastric reflux * | no, yes | Categorised 0/1 |
|  | 47 | Hiatus hernia * | no, yes | Categorised 0/1 |
|  | 48 | Gall stones * | no, yes | Categorised 0/1 |
|  | 49 | Diverticulitis * | no, yes | Categorised 0/1 |

FI, frailty index.

FI score as previously validated in UK Biobank by Williams et al.

†N= (39-73 year olds; European descent; complete case analysis of all 49 FI components).

* Participants reported medically diagnosed conditions for these items.

**Supplementary Table 2. Genetic regions used to identify instruments for each antihypertensive drug.**

| **Category** | **Gene** | **Probe ID** | **Chromsome** | **Cytogenetic band** | **Position (hg19/GRCh37)** | **Drug** |
| --- | --- | --- | --- | --- | --- | --- |
| ACEI | ACE | ENSG00000159640 | 17 | 17q23.3 | 61554422-61599205 | Benazepril, captopril, enalapril, lisinopril, trandopril, midalapril, fosinopril, ramipril, perindopril |
| ARB | AGTR1 | ENSG00000144891 | 3 | 3q24 | 148415571-148460795 | Losartan, candesartan, irbesartan, telmisartan, risartan, iprosartan, valsartan, olmesartan |
|  | PPARG | ENSG00000132170 | 3 | 3p25.2 | 12328867-12475855 | Telmisartan |
| BB | ADRB1 | ENSG00000043591 | 10 | 10q25.3 | 115803806-115806667 | Propranolol, oxyenolol, indolol, sotalol, atenolol, metoprolol, bisoprolol, nebolol, carvedilol, labetalol, arolol |
|  | ADRB2 | ENSG00000169252 | 5 | 5q32 | 148206156-148208196 | Sotalol, labelol, arolol |
|  | KCNH2 | ENSG00000055118 | 7 | 7q36.1 | 150642049-150675409 | Sotalol |
|  | ADRA1A | ENSG00000120907 | 8 | 8p21.2 | 26605667-26724795 | Carvedilol |
| CCB | CACNA1B | ENSG00000148408 | 9 | 9q34.3 | 140772234-141019076 | Benidipine, cilnidipine, felodipine |
|  | CACNA1C | ENSG00000151067 | 12 | 12p13.33 | 2162153-2807116 | Amlodipine, nifedipine, nitrendipine, nimodipine, Perdipine, felodipine, diltiazem, verapamil, eladipine |
|  | CACNA1D | ENSG00000157388 | 3 | 3p21.1 | 53528638-53847760 | Nifedipine, nitrendipine, nimodipine, Perdipine, felodipine, eladipine |
|  | CACNA2D1 | ENSG00000153956 | 7 | 7q21.11 | 81575760-82073114 | Nitrendipine, Perdipine, felodipine, iladipine |
|  | CACNA2D2 | ENSG00000007402 | 3 | 3p21.31 | 50400044-50541675 | Eladipine |
|  | CACNA1F | ENSG00000102001 | X |  |  | Nimodipine |
|  | CACNA1H | ENSG00000196557 | 16 | 16p13.3 | 1203106-1271771 | Eladipine |
|  | CACNA1I | ENSG00000100346 | 22 | 22q13.1 | 39966758-40085742 | Amlodipine |
|  | CACNA1S | ENSG00000081248 | 1 | 1q32.1 | 201008640-201081694 | Nitrendipine, nimodipine, eladipine |
|  | CACNB1 | ENSG00000067191 | 17 | 17q12 | 37329706-37353956 | Nimodipine |
|  | CACNB2 | ENSG00000165995 | 10 | 10p12.33-p12.31 | 18429353-18832486 | Nifedipine, nitrendipine, nimodipine, Perdipine, felodipine, eladipine |
|  | CACNB3 | ENSG00000167535 | 12 | 12q13.12 | 49207577-49222724 | Nimodipine |
|  | CACNB4 | ENSG00000182389 | 2 | 2q23.3 | 152689285-152955681 | Nimodipine |
|  | CACNG1 | ENSG00000108878 | 17 | 17q24.2 | 65040670-65052913 | Diltiazem |
| Thiazides | SLC12A1 | ENSG00000074803 | 15 | 15q21.1 | 48483861-48596275 | Chlorothiazone, quetiazone, furosemide, bumetanide, tolasemide |
|  | SLC12A2 | ENSG00000064651 | 5 | 5q23.3 | 127419458-127525380 | Quinthiazone, Bumetanide, Tolasemide |
|  | SLC12A3 | ENSG00000070915 | 16 | 16q13 | 56899119-56949762 | Hydrochlorothiazide, Benfluthiazide, Chlorothiazide, Indapamide, Metolazone, Quinthiazone |
|  | KCNMA1 | ENSG00000156113 | 10 | 10q22.3 | 78629359-79397566 | Hydrochlorothiazide, Benfluothiazide |
|  | CA1 | ENSG00000133742 | 8 | 8q21.2 | 86239837-86291243 | Chlorothiazide, Chlorothiazone, Quinthiazone |
|  | CA2 | ENSG00000104267 | 8 | 8q21.2 | 86376081-86393722 | Chlorothiazide, Quinthiazone |

ACEi, angiotensin converting enzyme inhibitor; ARB, angiotensin II receptor blocker ; BB, beta-blocker; CCB, calcium channel blocker.

**Supplementary Table 3. SNPs related to systolic BP to create genetic risk score in one-sample Mendelian randomization analysis (n=436).**

| **SNP** | **Chr** | **Pos** | **EA** | **OA** | **EAF** | **Beta** | **SE** | **P value** | **F statistic** |
| --- | --- | --- | --- | --- | --- | --- | --- | --- | --- |
| rs2493296 | 1 | 3327032 | T | C | 0.1425 | 0.418 | 0.044 | 3.137E-21 | 89.56 |
| rs404100 | 1 | 25366987 | T | C | 0.4513 | 0.194 | 0.03 | 1.683E-10 | 40.78 |
| rs11585169 | 1 | 150572037 | A | T | 0.5773 | 0.18 | 0.031 | 5.343E-09 | 34.00 |
| rs4651224 | 1 | 184585182 | T | C | 0.4474 | 0.199 | 0.031 | 8.999E-11 | 42.12 |
| rs12042924 | 1 | 197297417 | C | T | 0.4716 | 0.181 | 0.03 | 2.624E-09 | 35.57 |
| rs7555285 | 1 | 209970355 | C | G | 0.8011 | 0.229 | 0.038 | 1.054E-09 | 37.22 |
| rs11210029 | 1 | 41865293 | G | A | 0.3678 | 0.203 | 0.031 | 8.919E-11 | 42.06 |
| rs778124 | 1 | 56606206 | A | G | 0.3736 | 0.297 | 0.031 | 1.45E-21 | 90.89 |
| rs61772592 | 1 | 56979681 | G | A | 0.1255 | 0.318 | 0.046 | 2.862E-12 | 48.88 |
| rs12136922 | 1 | 67007389 | A | G | 0.4949 | 0.203 | 0.03 | 2.689E-11 | 44.46 |
| rs34079867 | 1 | 27407850 | T | C | 0.266 | 0.199 | 0.035 | 1.781E-08 | 31.66 |
| rs59980837 | 1 | 115827266 | T | G | 0.0178 | 1.1 | 0.116 | 3.315E-21 | 89.41 |
| rs68085857 | 1 | 217737629 | T | C | 0.234 | 0.274 | 0.036 | 1.678E-14 | 58.91 |
| rs4926499 | 1 | 249155909 | C | G | 0.8263 | 0.297 | 0.044 | 1.333E-11 | 45.82 |
| rs1889785 | 1 | 16348729 | A | G | 0.4552 | 0.178 | 0.03 | 4.351E-09 | 34.36 |
| rs12063372 | 1 | 59621911 | A | G | 0.3846 | 0.199 | 0.032 | 3.858E-10 | 39.12 |
| rs658780 | 1 | 78555928 | G | T | 0.2553 | 0.203 | 0.035 | 5.285E-09 | 34.16 |
| rs10776752 | 1 | 113044328 | T | G | 0.0809 | 0.821 | 0.058 | 4.61E-46 | 203.21 |
| rs2493134 | 1 | 230849359 | C | T | 0.4071 | 0.374 | 0.031 | 1.104E-33 | 146.18 |
| rs1565440 | 1 | 243387788 | A | G | 0.3752 | 0.175 | 0.031 | 1.935E-08 | 31.52 |
| rs10188003 | 2 | 66773469 | T | C | 0.393 | 0.188 | 0.031 | 8.799E-10 | 37.62 |
| rs6731373 | 2 | 68503044 | A | G | 0.3492 | 0.191 | 0.033 | 4.182E-09 | 34.43 |
| rs2580350 | 2 | 121996007 | A | G | 0.5609 | 0.177 | 0.031 | 8.393E-09 | 33.20 |
| rs11694601 | 2 | 174949358 | G | A | 0.4032 | 0.191 | 0.031 | 6.412E-10 | 38.17 |
| rs12694277 | 2 | 213188795 | C | T | 0.7054 | 0.202 | 0.034 | 1.796E-09 | 36.29 |
| rs17760259 | 2 | 19744462 | C | T | 0.4276 | 0.265 | 0.03 | 2.253E-18 | 76.22 |
| rs2384063 | 2 | 25187115 | T | C | 0.7607 | 0.327 | 0.036 | 6.33E-20 | 83.69 |
| rs55944332 | 2 | 145726621 | G | A | 0.2368 | 0.261 | 0.036 | 1.792E-13 | 54.18 |
| rs12693982 | 2 | 204085635 | T | C | 0.4024 | 0.258 | 0.031 | 7.49E-17 | 69.44 |
| rs268263 | 2 | 164954174 | A | T | 0.7498 | 0.594 | 0.035 | 1.77E-63 | 282.96 |
| rs13016772 | 2 | 55779476 | T | C | 0.7651 | 0.252 | 0.036 | 1.227E-12 | 50.47 |
| rs4577304 | 2 | 73403040 | C | T | 0.4767 | 0.177 | 0.03 | 4.987E-09 | 34.23 |
| rs3845811 | 2 | 208521512 | G | C | 0.4339 | 0.294 | 0.031 | 1.876E-21 | 90.65 |
| rs34727427 | 2 | 177016728 | C | T | 0.3168 | 0.235 | 0.032 | 4.017E-13 | 52.74 |
| rs2643826 | 3 | 27562988 | T | C | 0.4505 | 0.447 | 0.031 | 1.741E-48 | 213.68 |
| rs1375564 | 3 | 85656311 | T | C | 0.6395 | 0.258 | 0.032 | 2.844E-16 | 67.03 |
| rs12637573 | 3 | 121682388 | G | A | 0.5282 | 0.173 | 0.03 | 9.949E-09 | 32.85 |
| rs1199330 | 3 | 138101529 | G | A | 0.1176 | 0.265 | 0.047 | 1.654E-08 | 31.89 |
| rs13091418 | 3 | 185329756 | G | C | 0.3341 | 0.223 | 0.033 | 6.148E-12 | 47.25 |
| rs743395 | 3 | 37598382 | T | C | 0.3834 | 0.26 | 0.032 | 2.55E-16 | 67.12 |
| rs4499560 | 3 | 70920485 | T | A | 0.6829 | 0.22 | 0.033 | 1.463E-11 | 45.50 |
| rs9876694 | 3 | 141152017 | T | C | 0.0584 | 0.471 | 0.065 | 4.643E-13 | 52.41 |
| rs1290784 | 3 | 169096900 | T | C | 0.4483 | 0.412 | 0.03 | 2.968E-42 | 185.25 |
| rs68115553 | 3 | 27704702 | G | A | 0.0199 | 0.645 | 0.114 | 1.737E-08 | 31.79 |
| rs6771917 | 3 | 48108442 | C | T | 0.7523 | 0.379 | 0.036 | 1.389E-26 | 114.16 |
| rs9880098 | 3 | 133949366 | A | G | 0.3946 | 0.308 | 0.031 | 1.593E-23 | 100.06 |
| rs6788907 | 3 | 158212823 | A | G | 0.2682 | 0.221 | 0.034 | 7.31E-11 | 42.50 |
| rs9848170 | 3 | 11495983 | C | G | 0.597 | 0.323 | 0.031 | 7.01E-26 | 110.76 |
| rs4408839 | 3 | 153729768 | G | A | 0.2567 | 0.23 | 0.035 | 2.425E-11 | 44.48 |
| rs1052501 | 3 | 41925398 | T | C | 0.8329 | 0.226 | 0.041 | 4.143E-08 | 30.14 |
| rs6445583 | 3 | 53562894 | A | G | 0.7465 | 0.277 | 0.035 | 1.896E-15 | 63.18 |
| rs2111557 | 3 | 169325621 | T | C | 0.4675 | 0.176 | 0.03 | 5.221E-09 | 34.12 |
| rs72719160 | 4 | 144051276 | T | A | 0.3171 | 0.224 | 0.032 | 4.337E-12 | 47.93 |
| rs2610990 | 4 | 18008232 | G | A | 0.7359 | 0.29 | 0.034 | 2.863E-17 | 71.63 |
| rs7439567 | 4 | 138464842 | T | C | 0.4106 | 0.254 | 0.031 | 2.306E-16 | 67.41 |
| rs2353940 | 4 | 145740898 | C | T | 0.2493 | 0.208 | 0.036 | 6.846E-09 | 33.59 |
| rs2498323 | 4 | 3451109 | A | G | 0.098 | 0.317 | 0.052 | 8.515E-10 | 37.62 |
| rs12511987 | 4 | 46595623 | G | T | 0.1774 | 0.233 | 0.04 | 5.393E-09 | 34.07 |
| rs12509595 | 4 | 81182554 | C | T | 0.2923 | 0.837 | 0.033 | 2.55E-138 | 627.55 |
| rs17010957 | 4 | 86719165 | C | T | 0.1463 | 0.534 | 0.043 | 1.781E-35 | 154.22 |
| rs73855810 | 4 | 148383424 | A | G | 0.1406 | 0.273 | 0.043 | 3.043E-10 | 39.63 |
| rs55924432 | 4 | 26812737 | T | C | 0.401 | 0.265 | 0.032 | 5.695E-17 | 69.94 |
| rs1493132 | 4 | 108861082 | C | T | 0.3397 | 0.177 | 0.032 | 2.728E-08 | 30.84 |
| rs4834792 | 4 | 120555696 | A | T | 0.4796 | 0.197 | 0.03 | 7.241E-11 | 42.40 |
| rs34535756 | 4 | 2246927 | T | C | 0.0394 | 0.478 | 0.079 | 1.181E-09 | 36.98 |
| rs11097909 | 4 | 106911321 | C | T | 0.8528 | 0.363 | 0.043 | 3.353E-17 | 71.19 |
| rs12656497 | 5 | 32831939 | C | T | 0.5966 | 0.638 | 0.031 | 7.14E-96 | 432.15 |
| rs12657950 | 5 | 61940569 | T | C | 0.0744 | 0.455 | 0.059 | 1.269E-14 | 59.47 |
| rs1422279 | 5 | 122470209 | T | C | 0.3864 | 0.331 | 0.031 | 1.052E-26 | 114.75 |
| rs10045307 | 5 | 127411454 | G | C | 0.227 | 0.202 | 0.036 | 2.207E-08 | 31.26 |
| rs702395 | 5 | 140086677 | T | C | 0.4369 | 0.232 | 0.031 | 3.239E-14 | 57.76 |
| rs1957563 | 5 | 157474590 | T | C | 0.265 | 0.363 | 0.034 | 2.317E-26 | 112.60 |
| rs13358657 | 5 | 157938070 | G | A | 0.1332 | 0.388 | 0.045 | 2.95E-18 | 76.02 |
| rs246973 | 5 | 68007803 | T | C | 0.2882 | 0.248 | 0.034 | 1.453E-13 | 54.76 |
| rs10069690 | 5 | 1279790 | T | C | 0.2582 | 0.31 | 0.037 | 4.473E-17 | 70.49 |
| rs10941043 | 5 | 33194751 | G | T | 0.2902 | 0.259 | 0.033 | 6.415E-15 | 60.62 |
| rs7703560 | 5 | 67678506 | G | A | 0.2998 | 0.225 | 0.033 | 1.512E-11 | 45.49 |
| rs1664781 | 5 | 53276301 | A | G | 0.6925 | 0.264 | 0.033 | 5.692E-16 | 65.73 |
| rs28650790 | 5 | 55861464 | T | C | 0.1891 | 0.229 | 0.039 | 3.304E-09 | 34.92 |
| rs1871190 | 5 | 97953719 | T | G | 0.3349 | 0.195 | 0.032 | 1.656E-09 | 36.37 |
| rs6892983 | 5 | 127845030 | A | C | 0.4022 | 0.343 | 0.031 | 7.114E-29 | 124.61 |
| rs2913920 | 5 | 141726983 | T | C | 0.765 | 0.242 | 0.036 | 1.623E-11 | 45.37 |
| rs1984195 | 6 | 79657391 | A | G | 0.4887 | 0.241 | 0.03 | 1.768E-15 | 63.21 |
| rs9486916 | 6 | 109013930 | T | C | 0.1979 | 0.266 | 0.039 | 5.419E-12 | 47.63 |
| rs79782817 | 6 | 25882678 | T | G | 0.1027 | 0.532 | 0.05 | 1.426E-26 | 113.83 |
| rs1293969 | 6 | 151959945 | C | T | 0.2516 | 0.199 | 0.035 | 1.034E-08 | 32.82 |
| rs12661036 | 6 | 163737476 | C | T | 0.225 | 0.21 | 0.037 | 1.82E-08 | 31.65 |
| rs2815063 | 6 | 39262535 | A | C | 0.1315 | 0.276 | 0.046 | 1.763E-09 | 36.18 |
| rs10782230 | 6 | 126228512 | A | G | 0.4845 | 0.211 | 0.03 | 2.912E-12 | 48.63 |
| rs9401913 | 6 | 127159982 | A | G | 0.4387 | 0.52 | 0.031 | 3.656E-65 | 290.90 |
| rs8180684 | 6 | 143200936 | T | C | 0.2896 | 0.213 | 0.034 | 1.8E-10 | 40.58 |
| rs12528975 | 6 | 30988017 | A | G | 0.0489 | 0.475 | 0.075 | 1.983E-10 | 40.45 |
| rs9361836 | 6 | 82235408 | T | C | 0.3172 | 0.22 | 0.032 | 1.248E-11 | 45.94 |
| rs961764 | 6 | 117522156 | G | C | 0.5746 | 0.191 | 0.031 | 3.745E-10 | 39.18 |
| rs1575290 | 6 | 7715689 | T | C | 0.4733 | 0.197 | 0.03 | 5.586E-11 | 42.97 |
| rs9368222 | 6 | 20686996 | A | C | 0.2688 | 0.228 | 0.034 | 1.837E-11 | 45.27 |
| rs7763558 | 6 | 43349215 | A | G | 0.3241 | 0.336 | 0.032 | 1.17E-25 | 109.76 |
| rs78648104 | 6 | 50683009 | C | T | 0.0925 | 0.429 | 0.054 | 2.365E-15 | 62.79 |
| rs6921291 | 6 | 97066242 | T | C | 0.1907 | 0.358 | 0.039 | 1.575E-20 | 86.22 |
| rs2392929 | 7 | 106414069 | G | T | 0.2027 | 0.751 | 0.038 | 1.958E-87 | 392.33 |
| rs73049928 | 7 | 4669949 | G | A | 0.1939 | 0.238 | 0.039 | 1.197E-09 | 36.92 |
| rs3807925 | 7 | 18543250 | G | A | 0.3504 | 0.186 | 0.032 | 5.389E-09 | 33.96 |
| rs12668436 | 7 | 47548893 | C | T | 0.2459 | 0.215 | 0.035 | 7.883E-10 | 37.77 |
| rs35680304 | 7 | 130973495 | T | C | 0.5929 | 0.269 | 0.031 | 3.762E-18 | 75.52 |
| rs28688791 | 7 | 19039605 | C | T | 0.1982 | 0.322 | 0.038 | 2.335E-17 | 71.89 |
| rs73727605 | 7 | 149474622 | A | G | 0.0663 | 0.362 | 0.062 | 6.604E-09 | 33.69 |
| rs3918226 | 7 | 150690176 | T | C | 0.0811 | 0.664 | 0.058 | 8.461E-31 | 133.35 |
| rs10224210 | 7 | 151413194 | C | T | 0.2789 | 0.383 | 0.034 | 1.604E-29 | 126.96 |
| rs3735533 | 7 | 27245893 | C | T | 0.9257 | 0.91 | 0.058 | 5.291E-56 | 248.73 |
| rs6961048 | 7 | 27328187 | G | C | 0.104 | 0.53 | 0.05 | 1.43E-26 | 113.89 |
| rs848445 | 7 | 77572461 | C | T | 0.7149 | 0.203 | 0.034 | 2.284E-09 | 35.68 |
| rs75672964 | 7 | 131321010 | T | C | 0.0418 | 0.589 | 0.084 | 2.348E-12 | 49.20 |
| rs1906672 | 8 | 38130025 | A | G | 0.2319 | 0.297 | 0.036 | 1.204E-16 | 68.64 |
| rs4734868 | 8 | 92793786 | G | A | 0.3303 | 0.184 | 0.032 | 9.215E-09 | 33.00 |
| rs77375686 | 8 | 26043622 | G | A | 0.1117 | 0.347 | 0.049 | 8.377E-13 | 51.10 |
| rs4873492 | 8 | 51947549 | T | C | 0.1724 | 0.343 | 0.04 | 1.614E-17 | 72.48 |
| rs34917849 | 8 | 95278307 | C | G | 0.1268 | 0.312 | 0.045 | 5.966E-12 | 47.35 |
| rs13253358 | 8 | 68920135 | T | C | 0.2979 | 0.213 | 0.033 | 1.128E-10 | 41.54 |
| rs79069610 | 8 | 105921209 | C | T | 0.05 | 0.401 | 0.073 | 3.678E-08 | 30.35 |
| rs7012866 | 8 | 135616959 | G | T | 0.5009 | 0.233 | 0.03 | 1.211E-14 | 59.66 |
| rs4961293 | 8 | 141812374 | T | C | 0.4513 | 0.227 | 0.03 | 7.354E-14 | 56.03 |
| rs1410222 | 9 | 77239540 | T | C | 0.8166 | 0.217 | 0.039 | 2.165E-08 | 31.37 |
| rs10980408 | 9 | 113249071 | C | T | 0.0359 | 0.761 | 0.083 | 3.828E-20 | 84.59 |
| rs60191654 | 9 | 753648 | G | A | 0.1882 | 0.238 | 0.039 | 5.875E-10 | 38.28 |
| rs927315 | 9 | 4117713 | T | C | 0.4713 | 0.169 | 0.03 | 2.438E-08 | 31.07 |
| rs4245599 | 10 | 60365755 | G | A | 0.5416 | 0.179 | 0.031 | 4.035E-09 | 34.60 |
| rs111866816 | 10 | 94441507 | T | C | 0.0709 | 0.357 | 0.06 | 2.288E-09 | 35.74 |
| rs12264186 | 10 | 32289986 | T | C | 0.1871 | 0.214 | 0.039 | 3.584E-08 | 30.44 |
| rs2177843 | 10 | 75409877 | T | C | 0.1505 | 0.439 | 0.043 | 2.797E-24 | 103.46 |
| rs1006545 | 10 | 102553647 | T | G | 0.8872 | 0.685 | 0.048 | 3.497E-46 | 203.42 |
| rs740746 | 10 | 115792787 | A | G | 0.7318 | 0.456 | 0.034 | 1.421E-40 | 177.54 |
| rs12255372 | 10 | 114808902 | T | G | 0.2883 | 0.236 | 0.034 | 1.938E-12 | 49.54 |
| rs11592107 | 10 | 122968964 | A | G | 0.3096 | 0.302 | 0.033 | 1.547E-20 | 86.05 |
| rs1623474 | 10 | 18471794 | T | C | 0.3303 | 0.383 | 0.032 | 7.661E-33 | 142.14 |
| rs3802517 | 10 | 28233469 | A | T | 0.4618 | 0.253 | 0.03 | 4.648E-17 | 70.48 |
| rs117464403 | 10 | 107158054 | A | G | 0.0183 | 0.864 | 0.12 | 5.796E-13 | 51.93 |
| rs60444686 | 10 | 115717311 | A | G | 0.0406 | 0.591 | 0.078 | 4.424E-14 | 57.02 |
| rs7093894 | 10 | 124234880 | A | C | 0.1512 | 0.236 | 0.043 | 3.161E-08 | 30.55 |
| rs1133400 | 10 | 134459388 | G | A | 0.214 | 0.298 | 0.038 | 2.528E-15 | 62.60 |
| rs871004 | 11 | 28512458 | A | G | 0.3481 | 0.234 | 0.032 | 1.648E-13 | 54.30 |
| rs2904315 | 11 | 48109948 | G | A | 0.6869 | 0.208 | 0.033 | 1.577E-10 | 41.00 |
| rs604723 | 11 | 100610546 | C | T | 0.7244 | 0.655 | 0.034 | 2.546E-83 | 373.32 |
| rs74048190 | 11 | 2114221 | C | T | 0.0478 | 0.44 | 0.076 | 6.074E-09 | 33.85 |
| rs7107356 | 11 | 47676170 | G | A | 0.5041 | 0.46 | 0.03 | 1.63E-52 | 233.35 |
| rs10750441 | 11 | 130469044 | T | C | 0.6621 | 0.175 | 0.032 | 3.745E-08 | 30.23 |
| rs4980379 | 11 | 1888614 | T | C | 0.3719 | 0.576 | 0.032 | 2.467E-72 | 324.45 |
| rs2957688 | 11 | 10364963 | A | G | 0.4707 | 0.347 | 0.03 | 2.739E-30 | 130.44 |
| rs17762 | 11 | 22492454 | A | G | 0.0777 | 0.412 | 0.057 | 5.599E-13 | 51.99 |
| rs2014408 | 11 | 16365282 | T | C | 0.2087 | 0.517 | 0.037 | 1.259E-43 | 192.04 |
| rs177551 | 11 | 16930289 | A | C | 0.1344 | 0.373 | 0.044 | 3.468E-17 | 71.22 |
| rs11222084 | 11 | 130273230 | T | A | 0.3621 | 0.336 | 0.032 | 1.803E-26 | 113.26 |
| rs10501410 | 11 | 72088806 | A | G | 0.0692 | 0.412 | 0.061 | 1.102E-11 | 46.11 |
| rs78998485 | 12 | 434755 | G | C | 0.2557 | 0.245 | 0.035 | 1.479E-12 | 50.10 |
| rs7134440 | 12 | 53450097 | T | C | 0.0822 | 0.479 | 0.056 | 1.579E-17 | 72.58 |
| rs5742643 | 12 | 102837863 | C | T | 0.7513 | 0.223 | 0.035 | 1.525E-10 | 40.94 |
| rs117206641 | 12 | 133086888 | T | C | 0.1108 | 0.315 | 0.05 | 2.664E-10 | 39.95 |
| rs2129869 | 12 | 26457650 | T | A | 0.2222 | 0.264 | 0.036 | 2.443E-13 | 53.60 |
| rs61917655 | 12 | 48210787 | T | C | 0.1014 | 0.343 | 0.051 | 2.68E-11 | 44.45 |
| rs3819532 | 12 | 2436837 | C | T | 0.6087 | 0.188 | 0.031 | 9.436E-10 | 37.55 |
| rs7963801 | 12 | 79685226 | C | T | 0.5779 | 0.236 | 0.031 | 2.873E-14 | 57.68 |
| rs1169078 | 12 | 122416254 | G | C | 0.3121 | 0.197 | 0.033 | 1.677E-09 | 36.33 |
| rs6490019 | 12 | 115920472 | G | A | 0.6204 | 0.29 | 0.031 | 6.612E-21 | 87.90 |
| rs7306710 | 12 | 66376091 | C | T | 0.519 | 0.243 | 0.03 | 1.025E-15 | 64.26 |
| rs17245822 | 13 | 73131694 | C | A | 0.3733 | 0.19 | 0.031 | 1.152E-09 | 37.05 |
| rs7331680 | 13 | 115000650 | T | G | 0.1491 | 0.41 | 0.042 | 3.352E-22 | 93.99 |
| rs75961402 | 13 | 56398286 | A | G | 0.1534 | 0.266 | 0.042 | 1.945E-10 | 40.47 |
| rs483071 | 13 | 22294117 | T | C | 0.6248 | 0.271 | 0.031 | 5.093E-18 | 74.91 |
| rs7491248 | 13 | 47180671 | A | G | 0.2239 | 0.216 | 0.036 | 2.375E-09 | 35.70 |
| rs78474310 | 13 | 73826901 | G | A | 0.0448 | 0.47 | 0.073 | 1.512E-10 | 40.98 |
| rs9549627 | 13 | 113652369 | A | G | 0.1175 | 0.285 | 0.05 | 1.249E-08 | 32.40 |
| rs57786342 | 14 | 69260028 | A | G | 0.2059 | 0.232 | 0.037 | 5.626E-10 | 38.38 |
| rs7154723 | 14 | 98590629 | A | G | 0.385 | 0.253 | 0.031 | 2.721E-16 | 67.04 |
| rs8904 | 14 | 35871217 | A | G | 0.3678 | 0.306 | 0.031 | 1.713E-22 | 95.03 |
| rs3815460 | 14 | 73422259 | G | C | 0.1024 | 0.285 | 0.05 | 1.208E-08 | 32.49 |
| rs7493678 | 14 | 39400917 | T | A | 0.3486 | 0.189 | 0.032 | 2.308E-09 | 35.77 |
| rs35413927 | 14 | 53420358 | G | A | 0.3054 | 0.3 | 0.033 | 5.25E-20 | 83.77 |
| rs11159091 | 14 | 75074316 | A | G | 0.4615 | 0.198 | 0.03 | 6.792E-11 | 42.62 |
| rs12885878 | 14 | 104007555 | G | A | 0.7663 | 0.229 | 0.037 | 4.323E-10 | 38.97 |
| rs17562391 | 14 | 100133250 | T | C | 0.4186 | 0.197 | 0.031 | 1.349E-10 | 41.32 |
| rs4932373 | 15 | 91429287 | C | A | 0.3258 | 0.635 | 0.033 | 2.49E-83 | 374.80 |
| rs12906962 | 15 | 95312071 | C | T | 0.324 | 0.265 | 0.033 | 3.277E-16 | 66.64 |
| rs8030856 | 15 | 40314967 | G | C | 0.3953 | 0.176 | 0.031 | 1.212E-08 | 32.38 |
| rs2589218 | 15 | 96785017 | C | T | 0.2703 | 0.226 | 0.034 | 2.543E-11 | 44.37 |
| rs28866311 | 15 | 41442195 | G | T | 0.4737 | 0.276 | 0.03 | 5.453E-20 | 83.64 |
| rs28429256 | 15 | 66931617 | A | G | 0.3342 | 0.215 | 0.033 | 3.888E-11 | 43.76 |
| rs2627313 | 15 | 81006712 | T | C | 0.4454 | 0.321 | 0.03 | 3.552E-26 | 112.09 |
| rs4775769 | 15 | 48939888 | G | T | 0.9055 | 0.416 | 0.052 | 7.757E-16 | 64.81 |
| rs4784541 | 16 | 51704452 | C | T | 0.5252 | 0.202 | 0.031 | 4.927E-11 | 43.08 |
| rs1049212 | 16 | 4932929 | G | A | 0.5693 | 0.299 | 0.03 | 4.59E-23 | 98.02 |
| rs146550789 | 16 | 66781040 | C | T | 0.0417 | 0.482 | 0.078 | 5.638E-10 | 38.45 |
| rs12596630 | 16 | 2065666 | T | C | 0.0903 | 0.428 | 0.055 | 5.011E-15 | 61.17 |
| rs62047964 | 16 | 70729954 | T | C | 0.0622 | 0.512 | 0.069 | 9.294E-14 | 55.60 |
| rs1012089 | 16 | 74171973 | G | C | 0.5248 | 0.192 | 0.03 | 1.95E-10 | 40.42 |
| rs3950627 | 16 | 86436343 | A | C | 0.531 | 0.185 | 0.031 | 1.824E-09 | 36.12 |
| rs4888408 | 16 | 75432824 | A | G | 0.5855 | 0.365 | 0.031 | 1.415E-32 | 141.59 |
| rs8079811 | 17 | 1371473 | G | C | 0.6521 | 0.21 | 0.033 | 1.026E-10 | 41.79 |
| rs4925159 | 17 | 18185510 | A | G | 0.4246 | 0.217 | 0.031 | 9.656E-13 | 50.81 |
| rs17608766 | 17 | 45013271 | C | T | 0.1445 | 0.69 | 0.043 | 2.479E-57 | 254.16 |
| rs1551355 | 17 | 30032420 | T | C | 0.2334 | 0.21 | 0.036 | 3.886E-09 | 34.73 |
| rs9897429 | 17 | 47518378 | A | G | 0.52 | 0.265 | 0.032 | 1.189E-16 | 68.75 |
| rs1000423 | 17 | 59475642 | T | C | 0.7316 | 0.414 | 0.035 | 6.501E-33 | 143.03 |
| rs56288724 | 17 | 60767135 | G | A | 0.4169 | 0.218 | 0.031 | 2.011E-12 | 49.36 |
| rs2760748 | 17 | 2001604 | A | T | 0.0981 | 0.363 | 0.051 | 1.054E-12 | 50.75 |
| rs113086489 | 17 | 7171356 | T | C | 0.5525 | 0.325 | 0.031 | 3.803E-26 | 112.00 |
| rs7211535 | 17 | 19922364 | G | A | 0.5236 | 0.178 | 0.03 | 4.612E-09 | 34.25 |
| rs6504213 | 17 | 62381714 | C | T | 0.5818 | 0.298 | 0.031 | 1.248E-21 | 91.35 |
| rs56407827 | 18 | 42179819 | T | C | 0.2687 | 0.36 | 0.034 | 2.781E-26 | 112.30 |
| rs11874246 | 18 | 42596789 | T | C | 0.2963 | 0.286 | 0.033 | 3.225E-18 | 75.82 |
| rs1154214 | 18 | 24546824 | G | T | 0.6037 | 0.203 | 0.031 | 3.274E-11 | 44.05 |
| rs141958336 | 19 | 2165383 | A | G | 0.043 | 0.781 | 0.078 | 1.356E-23 | 100.18 |
| rs2291516 | 19 | 11508177 | A | G | 0.103 | 0.371 | 0.051 | 2.165E-13 | 53.91 |
| rs571689 | 19 | 49207554 | T | C | 0.5196 | 0.228 | 0.03 | 6.766E-14 | 56.25 |
| rs1848994 | 19 | 22111366 | A | G | 0.2828 | 0.201 | 0.033 | 1.787E-09 | 36.29 |
| rs28572357 | 19 | 31867447 | C | A | 0.3977 | 0.273 | 0.031 | 6.34E-19 | 78.74 |
| rs7255933 | 19 | 45766729 | A | G | 0.2574 | 0.231 | 0.035 | 2.441E-11 | 44.68 |
| rs6108787 | 20 | 10967214 | G | T | 0.4704 | 0.427 | 0.03 | 5.381E-46 | 202.97 |
| rs2801008 | 20 | 51788718 | G | T | 0.3183 | 0.188 | 0.032 | 7.369E-09 | 33.53 |
| rs6054139 | 20 | 6327810 | A | G | 0.606 | 0.209 | 0.031 | 8.228E-12 | 46.83 |
| rs8125763 | 20 | 17883531 | A | C | 0.4717 | 0.176 | 0.03 | 4.843E-09 | 34.23 |
| rs6026578 | 20 | 57463472 | G | C | 0.6269 | 0.185 | 0.032 | 4.594E-09 | 34.27 |
| rs79384779 | 20 | 31214944 | T | C | 0.1512 | 0.318 | 0.043 | 1.077E-13 | 55.17 |
| rs6026744 | 20 | 57742388 | T | A | 0.1229 | 0.713 | 0.046 | 6.998E-54 | 239.28 |
| rs6031431 | 20 | 42795152 | G | A | 0.4624 | 0.262 | 0.03 | 7.049E-18 | 74.11 |
| rs2833834 | 21 | 33814378 | A | C | 0.2765 | 0.218 | 0.034 | 1.217E-10 | 41.48 |
| rs12627651 | 21 | 44760603 | A | G | 0.2872 | 0.35 | 0.034 | 1.024E-24 | 105.23 |
| rs2776037 | 21 | 16317933 | C | T | 0.5849 | 0.185 | 0.031 | 2.146E-09 | 35.88 |
| rs1882961 | 21 | 16556367 | T | C | 0.3087 | 0.244 | 0.033 | 6.687E-14 | 56.16 |
| rs7278003 | 21 | 44966069 | C | T | 0.5622 | 0.188 | 0.03 | 6.628E-10 | 38.08 |
| rs112854918 | 22 | 30588910 | G | C | 0.0255 | 0.558 | 0.1 | 2.774E-08 | 30.86 |
| rs2238787 | 22 | 19976406 | A | G | 0.292 | 0.255 | 0.033 | 1.451E-14 | 59.09 |
| rs8142376 | 22 | 32001037 | T | C | 0.491 | 0.168 | 0.03 | 2.195E-08 | 31.21 |
| rs7796 | 1 | 1684169 | C | G | 0.4886 | 0.339 | 0.031 | 4.997E-27 | 116.21 |
| rs488834 | 1 | 10767902 | C | T | 0.7645 | 0.38 | 0.037 | 2.354E-25 | 108.33 |
| rs75461554 | 1 | 15810172 | C | T | 0.2007 | 0.302 | 0.038 | 1.178E-15 | 64.00 |
| rs1209384 | 1 | 43765089 | A | G | 0.6122 | 0.256 | 0.031 | 2.848E-16 | 66.79 |
| rs12731646 | 1 | 169090660 | C | T | 0.409 | 0.189 | 0.031 | 7.212E-10 | 37.90 |
| rs2724377 | 1 | 207974818 | A | G | 0.4697 | 0.194 | 0.03 | 1.286E-10 | 41.45 |
| rs72742507 | 1 | 221265336 | C | T | 0.2999 | 0.205 | 0.033 | 3.803E-10 | 39.18 |
| rs76719272 | 1 | 156129796 | C | T | 0.1312 | 0.274 | 0.046 | 2.97E-09 | 35.27 |
| rs263532 | 1 | 2164116 | T | C | 0.4245 | 0.18 | 0.031 | 4.716E-09 | 34.30 |
| rs7514579 | 1 | 94051350 | A | C | 0.2288 | 0.224 | 0.036 | 5.452E-10 | 38.61 |
| rs6699618 | 1 | 11881441 | C | G | 0.1599 | 0.912 | 0.041 | 1.68E-109 | 494.25 |
| rs10914124 | 1 | 180865798 | T | C | 0.3833 | 0.234 | 0.031 | 6.321E-14 | 56.25 |
| rs1408945 | 1 | 42364877 | G | T | 0.4243 | 0.32 | 0.03 | 8.331E-26 | 110.53 |
| rs786923 | 1 | 89242954 | C | T | 0.6239 | 0.308 | 0.031 | 2.825E-23 | 98.84 |
| rs11120093 | 1 | 207211326 | C | T | 0.4082 | 0.179 | 0.031 | 5.129E-09 | 34.07 |
| rs13420463 | 2 | 37517566 | A | G | 0.2266 | 0.314 | 0.036 | 2.715E-18 | 76.22 |
| rs115262049 | 2 | 43196694 | A | T | 0.0868 | 0.589 | 0.055 | 1.294E-26 | 113.97 |
| rs2249105 | 2 | 65287896 | A | G | 0.3679 | 0.293 | 0.031 | 7.635E-21 | 87.45 |
| rs2161967 | 2 | 218680529 | T | G | 0.5721 | 0.284 | 0.031 | 2.868E-20 | 85.34 |
| rs139354822 | 2 | 242344695 | T | C | 0.0296 | 0.612 | 0.098 | 3.505E-10 | 39.34 |
| rs1275985 | 2 | 26911745 | C | T | 0.6133 | 0.541 | 0.031 | 4.73E-69 | 308.64 |
| rs4952609 | 2 | 40555733 | A | G | 0.2561 | 0.212 | 0.035 | 9.604E-10 | 37.47 |
| rs17257081 | 2 | 135630498 | A | G | 0.1935 | 0.227 | 0.039 | 6.351E-09 | 33.65 |
| rs13412750 | 2 | 191634958 | G | A | 0.2708 | 0.289 | 0.034 | 2.325E-17 | 71.78 |
| rs1044822 | 2 | 230629138 | C | T | 0.1482 | 0.248 | 0.042 | 5.156E-09 | 34.21 |
| rs12464602 | 2 | 43397614 | G | A | 0.6208 | 0.244 | 0.032 | 1.016E-14 | 59.85 |
| rs6732123 | 2 | 69534650 | G | C | 0.4174 | 0.174 | 0.031 | 1.517E-08 | 32.01 |
| rs10207726 | 2 | 112744260 | C | T | 0.296 | 0.214 | 0.033 | 8.061E-11 | 42.13 |
| rs62170470 | 2 | 146989797 | T | C | 0.3983 | 0.197 | 0.032 | 7.685E-10 | 37.74 |
| rs72847885 | 2 | 86326717 | A | G | 0.337 | 0.241 | 0.032 | 3.079E-14 | 57.58 |
| rs6737318 | 2 | 114083120 | A | G | 0.2218 | 0.235 | 0.036 | 1.129E-10 | 41.61 |
| rs55732192 | 2 | 162278233 | G | T | 0.0947 | 0.336 | 0.052 | 1.153E-10 | 41.54 |
| rs1882212 | 2 | 182981968 | A | G | 0.2207 | 0.275 | 0.036 | 3.335E-14 | 57.52 |
| rs3828282 | 2 | 218779144 | C | G | 0.5721 | 0.186 | 0.032 | 5.294E-09 | 34.10 |
| rs10804330 | 2 | 227185749 | T | C | 0.4332 | 0.235 | 0.031 | 1.623E-14 | 59.03 |
| rs6788984 | 3 | 41107173 | A | G | 0.1437 | 0.3 | 0.043 | 3.806E-12 | 48.19 |
| rs3772219 | 3 | 56771251 | A | C | 0.3176 | 0.273 | 0.032 | 3.099E-17 | 71.15 |
| rs4955575 | 3 | 169534538 | A | C | 0.2539 | 0.216 | 0.035 | 5.631E-10 | 38.45 |
| rs9869437 | 3 | 196228360 | C | A | 0.3523 | 0.2 | 0.032 | 3.223E-10 | 39.59 |
| rs189267552 | 3 | 20073193 | T | A | 0.0132 | 0.866 | 0.139 | 4.55E-10 | 38.85 |
| rs7618284 | 3 | 66422246 | G | C | 0.3394 | 0.189 | 0.033 | 1.101E-08 | 32.64 |
| rs3980686 | 3 | 168697602 | G | T | 0.1075 | 0.5 | 0.049 | 1.027E-24 | 105.33 |
| rs262986 | 3 | 183435713 | G | A | 0.4704 | 0.237 | 0.031 | 7.667E-15 | 60.43 |
| rs7615099 | 3 | 53143901 | A | G | 0.3325 | 0.189 | 0.032 | 3.903E-09 | 34.70 |
| rs79539362 | 3 | 154680449 | T | C | 0.1008 | 0.4 | 0.05 | 2.087E-15 | 63.08 |
| rs11925504 | 3 | 14943965 | G | A | 0.5721 | 0.29 | 0.031 | 1.776E-21 | 90.47 |
| rs6438857 | 3 | 124557643 | T | C | 0.4226 | 0.274 | 0.031 | 3.132E-19 | 80.47 |
| rs60909079 | 4 | 83830244 | G | C | 0.2492 | 0.211 | 0.035 | 1.73E-09 | 36.27 |
| rs62309747 | 4 | 48713862 | G | A | 0.4734 | 0.224 | 0.03 | 1.593E-13 | 54.49 |
| rs1814951 | 4 | 111408718 | G | A | 0.8785 | 0.323 | 0.047 | 3.909E-12 | 48.07 |
| rs12643599 | 4 | 156639846 | A | G | 0.3605 | 0.313 | 0.031 | 1.232E-23 | 100.26 |
| rs2291434 | 4 | 38387244 | G | T | 0.5335 | 0.262 | 0.03 | 5.104E-18 | 74.88 |
| rs7683728 | 4 | 156402654 | C | T | 0.5312 | 0.365 | 0.03 | 2.431E-33 | 144.47 |
| rs1290933 | 4 | 2668217 | C | A | 0.6919 | 0.285 | 0.033 | 3.172E-18 | 75.80 |
| rs10028284 | 4 | 89752913 | A | T | 0.1816 | 0.294 | 0.04 | 1.691E-13 | 54.46 |
| rs13107325 | 4 | 103188709 | C | T | 0.0739 | 0.909 | 0.059 | 4.219E-53 | 235.56 |
| rs869396 | 4 | 169688000 | C | A | 0.4659 | 0.212 | 0.031 | 4.124E-12 | 48.09 |
| rs60991988 | 4 | 54801228 | T | G | 0.1069 | 0.379 | 0.05 | 2.82E-14 | 57.89 |
| rs13107261 | 4 | 63768826 | G | A | 0.3687 | 0.178 | 0.031 | 1.567E-08 | 32.06 |
| rs17035181 | 4 | 157678511 | T | G | 0.1448 | 0.307 | 0.043 | 7.614E-13 | 51.34 |
| rs11241313 | 5 | 114428167 | C | T | 0.3112 | 0.207 | 0.033 | 2.226E-10 | 40.36 |
| rs7722243 | 5 | 50818437 | G | A | 0.5043 | 0.205 | 0.03 | 1.21E-11 | 45.99 |
| rs3860770 | 5 | 173301427 | G | A | 0.2916 | 0.266 | 0.033 | 1.201E-15 | 63.95 |
| rs9327297 | 5 | 122835051 | C | G | 0.3324 | 0.275 | 0.032 | 8.067E-18 | 74.15 |
| rs7725413 | 5 | 15695987 | C | T | 0.7699 | 0.199 | 0.036 | 3.072E-08 | 30.57 |
| rs6870654 | 5 | 63831964 | T | C | 0.2546 | 0.214 | 0.035 | 7.581E-10 | 37.89 |
| rs73103937 | 5 | 66280577 | T | C | 0.268 | 0.205 | 0.034 | 2.355E-09 | 35.55 |
| rs76443575 | 5 | 96211594 | G | C | 0.0359 | 0.523 | 0.082 | 1.401E-10 | 41.13 |
| rs11960210 | 5 | 157817634 | T | C | 0.3755 | 0.473 | 0.031 | 1.253E-51 | 228.08 |
| rs12153395 | 5 | 179411477 | G | A | 0.1147 | 0.33 | 0.049 | 1.069E-11 | 46.19 |
| rs4957026 | 5 | 361148 | A | G | 0.6601 | 0.198 | 0.032 | 8.119E-10 | 37.65 |
| rs9349379 | 6 | 12903957 | A | G | 0.407 | 0.266 | 0.031 | 1.307E-17 | 72.91 |
| rs13204703 | 6 | 140692862 | T | C | 0.2489 | 0.197 | 0.035 | 1.943E-08 | 31.58 |
| rs2745599 | 6 | 1613686 | A | G | 0.448 | 0.216 | 0.032 | 8.956E-12 | 46.60 |
| rs1630736 | 6 | 12295987 | C | T | 0.465 | 0.171 | 0.031 | 3.516E-08 | 30.48 |
| rs9285476 | 6 | 134159976 | C | G | 0.2929 | 0.184 | 0.033 | 3.073E-08 | 30.66 |
| rs7765526 | 6 | 147713764 | A | G | 0.5367 | 0.201 | 0.031 | 5.882E-11 | 42.87 |
| rs17080102 | 6 | 151004770 | G | C | 0.0694 | 0.809 | 0.059 | 3.522E-42 | 185.26 |
| rs509833 | 6 | 159711515 | A | G | 0.8614 | 0.329 | 0.044 | 7.079E-14 | 55.91 |
| rs7744902 | 6 | 166176722 | G | A | 0.0766 | 0.409 | 0.059 | 5.638E-12 | 47.52 |
| rs67617547 | 7 | 90297177 | C | G | 0.3303 | 0.18 | 0.032 | 2.393E-08 | 31.21 |
| rs34072724 | 7 | 130432469 | G | A | 0.4889 | 0.242 | 0.03 | 1.373E-15 | 63.89 |
| rs10282122 | 7 | 2529623 | C | T | 0.6684 | 0.302 | 0.033 | 2.456E-20 | 85.29 |
| rs112509803 | 7 | 24735004 | G | C | 0.1138 | 0.264 | 0.048 | 3.179E-08 | 30.65 |
| rs11977526 | 7 | 46008110 | G | A | 0.4009 | 0.321 | 0.031 | 6.622E-25 | 106.05 |
| rs42032 | 7 | 92237426 | G | A | 0.2641 | 0.323 | 0.035 | 7.393E-21 | 87.71 |
| rs1870735 | 7 | 155744303 | C | G | 0.5469 | 0.206 | 0.031 | 3.605E-11 | 43.87 |
| rs7821832 | 8 | 25889446 | T | G | 0.2553 | 0.422 | 0.035 | 6.674E-34 | 147.19 |
| rs4440615 | 8 | 141057641 | G | A | 0.6321 | 0.22 | 0.031 | 1.874E-12 | 49.77 |
| rs9918876 | 8 | 77681097 | C | A | 0.1037 | 0.298 | 0.05 | 2.284E-09 | 35.69 |
| rs148401029 | 8 | 81386066 | C | A | 0.0352 | 0.462 | 0.085 | 4.968E-08 | 29.72 |
| rs2470004 | 8 | 120358445 | C | T | 0.8175 | 0.345 | 0.039 | 1.282E-18 | 77.64 |
| rs1821002 | 8 | 10640065 | C | G | 0.5892 | 0.379 | 0.031 | 5.192E-35 | 152.73 |
| rs2354862 | 8 | 64501744 | A | C | 0.3593 | 0.251 | 0.032 | 2.423E-15 | 62.54 |
| rs62512914 | 8 | 82828857 | A | G | 0.4152 | 0.207 | 0.031 | 1.418E-11 | 45.58 |
| rs1786345 | 8 | 101674751 | A | C | 0.4338 | 0.207 | 0.031 | 1.344E-11 | 45.60 |
| rs4260863 | 8 | 129386613 | C | G | 0.3837 | 0.191 | 0.031 | 1.171E-09 | 37.04 |
| rs7463212 | 8 | 143991858 | T | A | 0.5445 | 0.275 | 0.031 | 1.806E-19 | 81.47 |
| rs2126474 | 8 | 76878957 | G | T | 0.4125 | 0.26 | 0.031 | 1.871E-17 | 72.25 |
| rs35783704 | 8 | 105966258 | G | A | 0.1042 | 0.462 | 0.051 | 8.815E-20 | 83.00 |
| rs9886665 | 9 | 22942770 | T | C | 0.7329 | 0.205 | 0.034 | 2.472E-09 | 35.65 |
| rs3104552 | 9 | 128153616 | T | C | 0.4393 | 0.245 | 0.03 | 6.314E-16 | 65.33 |
| rs1332813 | 9 | 9350706 | T | C | 0.6486 | 0.22 | 0.031 | 2.317E-12 | 49.22 |
| rs76452347 | 9 | 35906471 | C | T | 0.205 | 0.297 | 0.04 | 7.129E-14 | 56.12 |
| rs7026176 | 9 | 116670743 | G | T | 0.5118 | 0.187 | 0.03 | 4.007E-10 | 39.07 |
| rs4553000 | 9 | 34223553 | C | T | 0.5141 | 0.204 | 0.03 | 1.094E-11 | 46.01 |
| rs7045409 | 9 | 95201540 | T | A | 0.3669 | 0.186 | 0.031 | 2.545E-09 | 35.39 |
| rs34025993 | 9 | 123516572 | A | G | 0.586 | 0.223 | 0.031 | 4.707E-13 | 52.42 |
| rs6271 | 9 | 136522274 | C | T | 0.0735 | 0.555 | 0.061 | 1.183E-19 | 82.42 |
| rs11145807 | 9 | 139520789 | A | G | 0.5943 | 0.214 | 0.032 | 3.537E-11 | 43.96 |
| rs4284362 | 10 | 45377839 | C | A | 0.7181 | 0.226 | 0.034 | 2.61E-11 | 44.55 |
| rs10749572 | 10 | 82136664 | G | T | 0.5444 | 0.203 | 0.03 | 1.88E-11 | 45.18 |
| rs2689690 | 10 | 95899706 | C | T | 0.3678 | 0.27 | 0.032 | 1.146E-17 | 73.11 |
| rs11252324 | 10 | 4124568 | G | T | 0.0771 | 0.416 | 0.057 | 3.613E-13 | 52.81 |
| rs12258967 | 10 | 18727959 | C | G | 0.2953 | 0.633 | 0.034 | 1.083E-78 | 352.48 |
| rs34130368 | 10 | 48411796 | G | T | 0.117 | 0.302 | 0.05 | 1.279E-09 | 36.83 |
| rs57946343 | 10 | 63499951 | T | C | 0.1473 | 0.716 | 0.043 | 2.102E-63 | 282.49 |
| rs11191580 | 10 | 104906211 | T | C | 0.0824 | 1.1 | 0.055 | 7.737E-89 | 399.64 |
| rs7912283 | 10 | 133773019 | G | A | 0.6468 | 0.214 | 0.032 | 2.938E-11 | 44.33 |
| rs2236295 | 10 | 64564892 | G | T | 0.3978 | 0.303 | 0.031 | 1.045E-22 | 96.03 |
| rs57866767 | 10 | 96023077 | T | C | 0.4322 | 0.45 | 0.03 | 1.138E-49 | 219.22 |
| rs67885470 | 11 | 99998431 | C | T | 0.2094 | 0.209 | 0.038 | 4.124E-08 | 30.16 |
| rs7926110 | 11 | 107086143 | T | G | 0.3267 | 0.26 | 0.032 | 5.711E-16 | 65.76 |
| rs1544861 | 11 | 10679441 | T | C | 0.6605 | 0.197 | 0.032 | 5.658E-10 | 38.34 |
| rs11604357 | 11 | 45351729 | C | A | 0.1622 | 0.277 | 0.041 | 1.601E-11 | 45.42 |
| rs66864335 | 11 | 65390803 | G | A | 0.2211 | 0.396 | 0.037 | 1.794E-27 | 117.89 |
| rs7395791 | 11 | 69262916 | G | A | 0.4419 | 0.216 | 0.031 | 2.193E-12 | 49.27 |
| rs2289124 | 11 | 89224477 | G | A | 0.1673 | 0.308 | 0.042 | 1.135E-13 | 55.08 |
| rs1382472 | 11 | 27273967 | G | A | 0.4041 | 0.192 | 0.031 | 4.466E-10 | 38.99 |
| rs2276153 | 11 | 58407740 | C | G | 0.243 | 0.33 | 0.035 | 6.555E-21 | 88.18 |
| rs72931748 | 11 | 69825414 | A | G | 0.0985 | 0.397 | 0.053 | 6.4E-14 | 56.24 |
| rs573455 | 11 | 117267884 | A | G | 0.539 | 0.199 | 0.03 | 4.772E-11 | 43.31 |
| rs74538877 | 11 | 67976593 | G | C | 0.0557 | 0.386 | 0.071 | 4.966E-08 | 29.73 |
| rs17249754 | 12 | 90060586 | G | A | 0.1683 | 0.845 | 0.04 | 1.25E-97 | 439.23 |
| rs10777213 | 12 | 90349999 | G | A | 0.5244 | 0.179 | 0.03 | 2.452E-09 | 35.68 |
| rs1010064 | 12 | 20000315 | A | C | 0.1837 | 0.357 | 0.039 | 3.02E-20 | 85.14 |
| rs7134677 | 12 | 54441498 | C | T | 0.2978 | 0.385 | 0.033 | 4.456E-31 | 134.55 |
| rs7980644 | 12 | 79959658 | A | G | 0.8334 | 0.264 | 0.04 | 6.297E-11 | 42.73 |
| rs2024385 | 12 | 12888438 | T | A | 0.424 | 0.264 | 0.031 | 5.876E-18 | 74.55 |
| rs73075659 | 12 | 20373541 | A | G | 0.3346 | 0.396 | 0.032 | 5.521E-35 | 152.34 |
| rs12426261 | 12 | 50573037 | A | G | 0.6208 | 0.378 | 0.031 | 2.314E-34 | 149.25 |
| rs4143175 | 12 | 67782397 | T | C | 0.7591 | 0.219 | 0.035 | 5.104E-10 | 38.60 |
| rs7310615 | 12 | 111865049 | C | G | 0.5184 | 0.585 | 0.031 | 1.318E-81 | 365.48 |
| rs1896326 | 12 | 115342956 | G | A | 0.2291 | 0.28 | 0.037 | 4.405E-14 | 56.84 |
| rs35444 | 12 | 115552437 | A | G | 0.3862 | 0.437 | 0.031 | 3.467E-45 | 198.54 |
| rs9507885 | 13 | 27951090 | C | T | 0.0953 | 0.321 | 0.054 | 3.233E-09 | 35.03 |
| rs9508495 | 13 | 30146201 | C | T | 0.7565 | 0.356 | 0.035 | 6.343E-24 | 101.54 |
| rs9526707 | 13 | 51489186 | G | A | 0.3216 | 0.204 | 0.032 | 2.768E-10 | 39.85 |
| rs6562778 | 13 | 74223828 | A | G | 0.5411 | 0.178 | 0.03 | 4.955E-09 | 34.28 |
| rs365990 | 14 | 23861811 | A | G | 0.3658 | 0.225 | 0.031 | 5.953E-13 | 52.01 |
| rs72683923 | 14 | 50735947 | T | C | 0.0212 | 0.959 | 0.11 | 3.08E-18 | 75.82 |
| rs12883810 | 14 | 68032235 | C | T | 0.1462 | 0.238 | 0.043 | 2.7E-08 | 30.97 |
| rs75016974 | 14 | 100197940 | C | T | 0.1423 | 0.251 | 0.044 | 1.047E-08 | 32.77 |
| rs8003103 | 14 | 71451265 | G | A | 0.3447 | 0.176 | 0.032 | 3.599E-08 | 30.27 |
| rs2652812 | 15 | 63406170 | C | T | 0.7544 | 0.252 | 0.035 | 1.027E-12 | 50.80 |
| rs11636952 | 15 | 75114322 | T | C | 0.6859 | 0.531 | 0.033 | 4.224E-59 | 262.38 |
| rs1994158 | 15 | 86064327 | A | G | 0.1807 | 0.251 | 0.039 | 1.233E-10 | 41.31 |
| rs4606697 | 15 | 100087596 | G | A | 0.1041 | 0.32 | 0.052 | 9.711E-10 | 37.34 |
| rs3098186 | 15 | 50810621 | C | T | 0.5156 | 0.242 | 0.03 | 1.411E-15 | 63.89 |
| rs6540119 | 16 | 87984477 | A | T | 0.666 | 0.202 | 0.032 | 3.929E-10 | 39.20 |
| rs35098810 | 16 | 60635748 | A | C | 0.2317 | 0.197 | 0.036 | 3.198E-08 | 30.53 |
| rs2283500 | 16 | 4138378 | A | C | 0.1102 | 0.311 | 0.048 | 1.077E-10 | 41.70 |
| rs908951 | 16 | 89697625 | C | T | 0.4378 | 0.226 | 0.032 | 7.135E-13 | 51.52 |
| rs11641374 | 16 | 1347717 | C | A | 0.5995 | 0.194 | 0.031 | 3.26E-10 | 39.54 |
| rs7186298 | 16 | 21088031 | C | T | 0.4295 | 0.232 | 0.03 | 1.882E-14 | 58.76 |
| rs12926550 | 16 | 81510155 | G | A | 0.3156 | 0.255 | 0.032 | 3.426E-15 | 61.85 |
| rs77924615 | 16 | 20392332 | G | A | 0.1986 | 0.408 | 0.039 | 1.123E-25 | 109.50 |
| rs8044992 | 16 | 24811207 | T | C | 0.2877 | 0.214 | 0.033 | 1.068E-10 | 41.72 |
| rs34941092 | 16 | 50550137 | G | A | 0.1498 | 0.323 | 0.043 | 3.234E-14 | 57.58 |
| rs79930761 | 17 | 7815712 | C | T | 0.0872 | 0.469 | 0.056 | 4.899E-17 | 70.33 |
| rs9302885 | 17 | 76799898 | A | G | 0.5548 | 0.224 | 0.03 | 1.034E-13 | 55.11 |
| rs9899540 | 17 | 30777924 | A | T | 0.6001 | 0.201 | 0.032 | 1.865E-10 | 40.50 |
| rs7213273 | 17 | 43155914 | G | A | 0.655 | 0.4 | 0.032 | 6.24E-37 | 161.25 |
| rs62076622 | 17 | 61090958 | A | G | 0.1987 | 0.236 | 0.038 | 3.785E-10 | 39.29 |
| rs4511593 | 17 | 7455536 | C | T | 0.6528 | 0.288 | 0.032 | 1.279E-19 | 82.08 |
| rs3764400 | 17 | 46123932 | T | C | 0.1365 | 0.375 | 0.045 | 3.689E-17 | 70.94 |
| rs11655604 | 17 | 79365861 | C | T | 0.3579 | 0.203 | 0.033 | 1.086E-09 | 37.27 |
| rs1436138 | 17 | 75316880 | A | G | 0.3633 | 0.312 | 0.032 | 4.727E-23 | 98.04 |
| rs34413141 | 18 | 777282 | T | A | 0.1822 | 0.353 | 0.039 | 2.469E-19 | 80.73 |
| rs10048404 | 18 | 54578482 | C | T | 0.3701 | 0.261 | 0.032 | 1.907E-16 | 67.63 |
| rs665445 | 18 | 51842682 | C | A | 0.2794 | 0.191 | 0.033 | 1.152E-08 | 32.67 |
| rs62082230 | 18 | 22676071 | T | A | 0.2773 | 0.188 | 0.035 | 4.689E-08 | 29.82 |
| rs1437649 | 18 | 48132646 | G | A | 0.2345 | 0.219 | 0.036 | 8.573E-10 | 37.60 |
| rs10460108 | 18 | 73034151 | A | G | 0.5199 | 0.214 | 0.03 | 1.12E-12 | 50.59 |
| rs34518929 | 19 | 18455444 | G | A | 0.2623 | 0.22 | 0.035 | 1.793E-10 | 40.66 |
| rs1433121 | 19 | 32591878 | C | T | 0.6906 | 0.228 | 0.033 | 2.655E-12 | 48.91 |
| rs12610654 | 19 | 5006598 | A | G | 0.3436 | 0.232 | 0.032 | 4.407E-13 | 52.34 |
| rs12978472 | 19 | 7257990 | C | G | 0.1241 | 0.845 | 0.049 | 1.228E-66 | 297.39 |
| rs10420519 | 19 | 45298461 | G | T | 0.0347 | 0.492 | 0.089 | 2.856E-08 | 30.78 |
| rs8113613 | 19 | 10350649 | C | T | 0.1813 | 0.233 | 0.04 | 4.587E-09 | 34.39 |
| rs73046792 | 19 | 49605705 | G | A | 0.1588 | 0.355 | 0.043 | 7.234E-17 | 69.60 |
| rs2423514 | 20 | 10693337 | A | G | 0.4589 | 0.301 | 0.03 | 1.767E-23 | 99.40 |
| rs6078093 | 20 | 11168669 | G | A | 0.428 | 0.185 | 0.03 | 1.195E-09 | 36.99 |
| rs6058088 | 20 | 30139886 | T | G | 0.1561 | 0.283 | 0.042 | 1.144E-11 | 46.12 |
| rs6029756 | 20 | 40266681 | G | A | 0.3225 | 0.271 | 0.033 | 1.884E-16 | 67.54 |
| rs2598 | 20 | 47241618 | A | G | 0.467 | 0.168 | 0.03 | 2.87E-08 | 30.74 |
| rs6090907 | 20 | 47410231 | G | A | 0.147 | 0.385 | 0.043 | 1.286E-19 | 82.23 |
| rs17812022 | 20 | 19007099 | C | T | 0.0958 | 0.361 | 0.053 | 5.645E-12 | 47.36 |
| rs6062324 | 20 | 62446351 | G | A | 0.2364 | 0.329 | 0.036 | 1.184E-19 | 82.34 |
| rs34487963 | 21 | 44838330 | C | A | 0.0185 | 0.882 | 0.124 | 1.35E-12 | 50.26 |
| rs28578714 | 22 | 50727921 | T | C | 0.3938 | 0.207 | 0.033 | 2.528E-10 | 39.92 |
| rs12321 | 22 | 29453193 | G | C | 0.4328 | 0.229 | 0.03 | 3.814E-14 | 57.22 |
| rs148140538 | 22 | 50228044 | C | T | 0.0808 | 0.325 | 0.056 | 7.391E-09 | 33.48 |

SNP, single nucleotide polymorphism; Chr, chromosome; Pos, position (hg19); EA, effect allele; OA, other allele; EAF, effect allele frequency; SE: standard error.

Supplementary Table 4. SNPs related to diastolic BP to create genetic risk score in one-sample Mendelian randomization analysis (n=441).

| **SNP** | **Chr** | **Pos** | **EA** | **OA** | **EAF** | **Beta** | **SE** | **P value** | **F statistic** |
| --- | --- | --- | --- | --- | --- | --- | --- | --- | --- |
| rs12728150 | 1 | 27268737 | G | A | 0.081 | 0.2045 | 0.0318 | 1.28E-10 | 41.36 |
| rs72704264 | 1 | 145713305 | C | G | 0.2172 | 0.117 | 0.0212 | 3.603E-08 | 30.46 |
| rs7524019 | 1 | 167367193 | T | C | 0.492 | 0.1036 | 0.0174 | 2.6E-09 | 35.45 |
| rs602521 | 1 | 227311066 | A | G | 0.2656 | 0.1351 | 0.0195 | 3.9719E-12 | 48.00 |
| rs1889785 | 1 | 16348729 | A | G | 0.4551 | 0.1255 | 0.0174 | 5.6131E-13 | 52.02 |
| rs710249 | 1 | 43869235 | C | G | 0.4264 | 0.1501 | 0.0174 | 6.4284E-18 | 74.42 |
| rs10493408 | 1 | 66992054 | A | C | 0.1331 | 0.1584 | 0.0255 | 5.092E-10 | 38.59 |
| rs150816167 | 1 | 179571862 | C | T | 0.0451 | 0.2873 | 0.0446 | 1.17E-10 | 41.50 |
| rs4926499 | 1 | 249155909 | C | G | 0.826 | 0.1694 | 0.0248 | 9.3735E-12 | 46.66 |
| rs10776752 | 1 | 113044328 | T | G | 0.0805 | 0.4573 | 0.033 | 1.2471E-43 | 192.03 |
| rs68085857 | 1 | 217737629 | T | C | 0.234 | 0.191 | 0.0205 | 9.8243E-21 | 86.81 |
| rs12088448 | 1 | 218546170 | C | A | 0.356 | 0.1544 | 0.0182 | 2.5322E-17 | 71.97 |
| rs3943093 | 1 | 243458502 | T | C | 0.3234 | 0.2477 | 0.0184 | 3.9446E-41 | 181.22 |
| rs2493296 | 1 | 3327032 | T | C | 0.1419 | 0.2496 | 0.0254 | 7.4508E-23 | 96.57 |
| rs4926901 | 1 | 48025824 | A | G | 0.3548 | 0.0984 | 0.018 | 4.819E-08 | 29.88 |
| rs2169137 | 1 | 204497913 | C | G | 0.7287 | 0.1588 | 0.0194 | 3.1667E-16 | 67.00 |
| rs964941 | 1 | 228193857 | A | G | 0.5183 | 0.1726 | 0.0174 | 3.3098E-23 | 98.40 |
| rs6686889 | 1 | 25030470 | T | C | 0.2533 | 0.1918 | 0.0199 | 6.9454E-22 | 92.89 |
| rs57748895 | 1 | 115826169 | T | A | 0.0179 | 0.6627 | 0.0666 | 2.4929E-23 | 99.01 |
| rs2493136 | 1 | 230851536 | T | C | 0.4075 | 0.2345 | 0.0176 | 1.9249E-40 | 177.53 |
| rs2586970 | 2 | 55829967 | G | A | 0.5639 | 0.1493 | 0.0175 | 1.5632E-17 | 72.79 |
| rs1876490 | 2 | 73052351 | A | G | 0.7167 | 0.1364 | 0.0192 | 1.1569E-12 | 50.47 |
| rs12990959 | 2 | 148572160 | C | T | 0.3125 | 0.1271 | 0.0187 | 1.1069E-11 | 46.20 |
| rs2444769 | 2 | 158494100 | A | C | 0.7949 | 0.158 | 0.0219 | 4.8462E-13 | 52.05 |
| rs4507125 | 2 | 239864732 | C | A | 0.2136 | 0.1244 | 0.0211 | 3.603E-09 | 34.76 |
| rs1373780 | 2 | 19501029 | C | G | 0.1845 | 0.1246 | 0.0224 | 2.582E-08 | 30.94 |
| rs824523 | 2 | 19707855 | A | C | 0.3344 | 0.1226 | 0.0183 | 2.2568E-11 | 44.88 |
| rs62155750 | 2 | 96491456 | G | A | 0.3074 | 0.2177 | 0.0196 | 8.2661E-29 | 123.37 |
| rs7569128 | 2 | 191446691 | A | C | 0.8185 | 0.1981 | 0.0225 | 1.2241E-18 | 77.52 |
| rs4675682 | 2 | 208402750 | C | T | 0.4622 | 0.1409 | 0.0173 | 4.4885E-16 | 66.33 |
| rs56809883 | 2 | 37886495 | T | G | 0.2595 | 0.1102 | 0.0199 | 3.033E-08 | 30.67 |
| rs55944332 | 2 | 145726621 | G | A | 0.2368 | 0.2365 | 0.0204 | 3.2696E-31 | 134.40 |
| rs7572130 | 2 | 164460947 | G | A | 0.1042 | 0.1796 | 0.0287 | 4.12E-10 | 39.16 |
| rs6546810 | 2 | 73389716 | C | T | 0.3525 | 0.12 | 0.0181 | 3.1623E-11 | 43.95 |
| rs1446468 | 2 | 164963486 | C | T | 0.546 | 0.253 | 0.0174 | 1.207E-47 | 211.42 |
| rs1263671 | 2 | 207996447 | C | T | 0.1632 | 0.1394 | 0.0238 | 4.6911E-09 | 34.31 |
| rs11923667 | 3 | 101268080 | A | T | 0.4071 | 0.1175 | 0.0177 | 3.0981E-11 | 44.07 |
| rs6777317 | 3 | 197070959 | A | G | 0.2899 | 0.1249 | 0.0195 | 1.505E-10 | 41.03 |
| rs2643826 | 3 | 27562988 | T | C | 0.4508 | 0.1857 | 0.0175 | 2.8327E-26 | 112.60 |
| rs6800730 | 3 | 48174210 | G | A | 0.6702 | 0.2476 | 0.0185 | 8.0039E-41 | 179.13 |
| rs78151625 | 3 | 158316726 | C | T | 0.1658 | 0.1869 | 0.0233 | 1.039E-15 | 64.34 |
| rs347585 | 3 | 11286220 | T | C | 0.7014 | 0.1506 | 0.0189 | 1.5671E-15 | 63.49 |
| rs62234672 | 3 | 16592069 | A | C | 0.1752 | 0.1248 | 0.0229 | 4.923E-08 | 29.70 |
| rs114714860 | 3 | 41882905 | C | G | 0.1683 | 0.33 | 0.0236 | 1.42E-44 | 195.53 |
| rs9841978 | 3 | 53730735 | A | G | 0.3251 | 0.1766 | 0.0185 | 1.1151E-21 | 91.13 |
| rs11130602 | 3 | 57947168 | A | G | 0.4427 | 0.1467 | 0.0175 | 4.2658E-17 | 70.27 |
| rs3774702 | 3 | 63856870 | A | G | 0.1768 | 0.147 | 0.0228 | 1.175E-10 | 41.57 |
| rs11923343 | 3 | 85668570 | G | A | 0.6396 | 0.1138 | 0.0181 | 3.101E-10 | 39.53 |
| rs4077158 | 3 | 133942941 | C | T | 0.5286 | 0.1832 | 0.0173 | 3.0889E-26 | 112.14 |
| rs6763931 | 3 | 141102833 | A | G | 0.4438 | 0.1383 | 0.0173 | 1.4812E-15 | 63.91 |
| rs6779368 | 3 | 185298868 | G | A | 0.3423 | 0.1791 | 0.0184 | 2.2798E-22 | 94.74 |
| rs3864004 | 3 | 41240177 | A | G | 0.4685 | 0.1004 | 0.0173 | 6.278E-09 | 33.68 |
| rs12152463 | 3 | 122100447 | T | C | 0.4251 | 0.1006 | 0.0174 | 8.0149E-09 | 33.43 |
| rs12509595 | 4 | 81182554 | C | T | 0.2924 | 0.4972 | 0.0192 | 1.581E-148 | 670.59 |
| rs72719149 | 4 | 144043336 | C | T | 0.3164 | 0.1279 | 0.0186 | 6.3416E-12 | 47.28 |
| rs62301873 | 4 | 40603821 | G | A | 0.1061 | 0.1734 | 0.0284 | 1.063E-09 | 37.28 |
| rs990619 | 4 | 156507678 | G | C | 0.5234 | 0.1592 | 0.0173 | 2.9027E-20 | 84.68 |
| rs28667801 | 4 | 26785356 | T | A | 0.407 | 0.1622 | 0.018 | 1.9028E-19 | 81.20 |
| rs61789369 | 4 | 2265295 | G | A | 0.0435 | 0.3039 | 0.0436 | 3.0648E-12 | 48.58 |
| rs72976750 | 4 | 86725684 | C | T | 0.1396 | 0.1718 | 0.0251 | 7.3672E-12 | 46.85 |
| rs7694000 | 4 | 95324968 | T | A | 0.4613 | 0.0965 | 0.0175 | 3.467E-08 | 30.41 |
| rs66887589 | 4 | 120509279 | C | T | 0.4779 | 0.161 | 0.0174 | 1.834E-20 | 85.62 |
| rs9286351 | 4 | 138441530 | G | A | 0.4188 | 0.1412 | 0.0177 | 1.6051E-15 | 63.64 |
| rs13124515 | 4 | 145403713 | C | T | 0.6869 | 0.1052 | 0.0187 | 1.984E-08 | 31.65 |
| rs13355146 | 5 | 92023661 | T | C | 0.3832 | 0.1224 | 0.0178 | 6.3915E-12 | 47.28 |
| rs2921604 | 5 | 14867948 | C | T | 0.4633 | 0.096 | 0.0176 | 4.4561E-08 | 29.75 |
| rs10941043 | 5 | 33194751 | G | T | 0.2906 | 0.1269 | 0.019 | 2.5241E-11 | 44.61 |
| rs3776299 | 5 | 142507651 | A | G | 0.4559 | 0.1266 | 0.0175 | 5.0641E-13 | 52.33 |
| rs13358657 | 5 | 157938070 | G | A | 0.1332 | 0.224 | 0.0255 | 1.6959E-18 | 77.16 |
| rs12656497 | 5 | 32831939 | C | T | 0.5968 | 0.3063 | 0.0176 | 1.474E-67 | 302.88 |
| rs1871190 | 5 | 97953719 | T | G | 0.3344 | 0.1078 | 0.0186 | 6.63E-09 | 33.59 |
| rs17677603 | 5 | 127857493 | G | A | 0.3837 | 0.2 | 0.0178 | 3.9003E-29 | 126.25 |
| rs1212061 | 5 | 141723403 | C | G | 0.7324 | 0.1281 | 0.0197 | 7.9086E-11 | 42.28 |
| rs3117736 | 5 | 157462999 | T | C | 0.2661 | 0.2374 | 0.0196 | 9.7051E-34 | 146.71 |
| rs10069690 | 5 | 1279790 | T | C | 0.2581 | 0.1615 | 0.021 | 1.4181E-14 | 59.14 |
| rs10054208 | 5 | 55688992 | T | C | 0.3617 | 0.1187 | 0.0185 | 1.494E-10 | 41.17 |
| rs12515541 | 5 | 57095011 | T | G | 0.6072 | 0.1156 | 0.0177 | 6.2287E-11 | 42.65 |
| rs10062049 | 5 | 61553881 | T | C | 0.1359 | 0.2208 | 0.0255 | 4.4957E-18 | 74.98 |
| rs4704514 | 5 | 77820081 | T | C | 0.2833 | 0.1087 | 0.0193 | 1.713E-08 | 31.72 |
| rs1582931 | 5 | 122657199 | A | G | 0.4748 | 0.2161 | 0.0175 | 4.5051E-35 | 152.49 |
| rs1114347 | 6 | 51834297 | G | A | 0.4823 | 0.1792 | 0.0173 | 3.3197E-25 | 107.30 |
| rs509067 | 6 | 117462040 | C | T | 0.5863 | 0.1436 | 0.0175 | 2.6479E-16 | 67.33 |
| rs440454 | 6 | 31927342 | G | A | 0.684 | 0.2602 | 0.0192 | 7.5232E-42 | 183.66 |
| rs76785130 | 6 | 121813835 | G | A | 0.0199 | 0.4285 | 0.0662 | 9.3648E-11 | 41.90 |
| rs13215166 | 6 | 127164360 | G | A | 0.4415 | 0.3094 | 0.0174 | 1.791E-70 | 316.19 |
| rs35261542 | 6 | 20675792 | A | C | 0.2679 | 0.1196 | 0.0195 | 9.288E-10 | 37.62 |
| rs9467545 | 6 | 25638464 | T | A | 0.1572 | 0.2545 | 0.0237 | 7.3875E-27 | 115.31 |
| rs115447786 | 6 | 34354073 | T | C | 0.0427 | 0.2904 | 0.0455 | 1.747E-10 | 40.74 |
| rs3798293 | 6 | 97033370 | G | A | 0.2165 | 0.1328 | 0.021 | 2.695E-10 | 39.99 |
| rs1799945 | 6 | 26091179 | G | C | 0.1497 | 0.3888 | 0.0244 | 3.8761E-57 | 253.91 |
| rs6905288 | 6 | 43758873 | A | G | 0.5681 | 0.1759 | 0.0179 | 7.7911E-23 | 96.57 |
| rs881858 | 6 | 43806609 | A | G | 0.694 | 0.1553 | 0.0191 | 4.6537E-16 | 66.11 |
| rs1984195 | 6 | 79657391 | A | G | 0.4883 | 0.1736 | 0.0173 | 1.4269E-23 | 100.69 |
| rs16875357 | 6 | 85652904 | G | T | 0.2431 | 0.1205 | 0.0203 | 2.695E-09 | 35.24 |
| rs72613227 | 6 | 106320771 | T | A | 0.1269 | 0.1884 | 0.0285 | 3.8699E-11 | 43.70 |
| rs9791312 | 6 | 143142313 | C | A | 0.3452 | 0.1225 | 0.0184 | 2.8927E-11 | 44.32 |
| rs9406076 | 6 | 8023804 | T | C | 0.3278 | 0.101 | 0.0185 | 4.649E-08 | 29.81 |
| rs6934891 | 6 | 22139729 | A | G | 0.4255 | 0.1275 | 0.0177 | 5.2144E-13 | 51.89 |
| rs2397060 | 6 | 51611470 | C | T | 0.1405 | 0.161 | 0.0251 | 1.461E-10 | 41.14 |
| rs10279432 | 7 | 7279010 | A | C | 0.6234 | 0.1178 | 0.018 | 5.7346E-11 | 42.83 |
| rs17432462 | 7 | 18548613 | C | T | 0.3766 | 0.1036 | 0.0179 | 7.309E-09 | 33.50 |
| rs1044608 | 7 | 150502016 | G | C | 0.0767 | 0.2018 | 0.0339 | 2.763E-09 | 35.44 |
| rs6464165 | 7 | 151413124 | C | T | 0.2809 | 0.217 | 0.0195 | 7.3401E-29 | 123.84 |
| rs310597 | 7 | 150873046 | G | A | 0.3695 | 0.1152 | 0.0181 | 2.179E-10 | 40.51 |
| rs7805035 | 7 | 25965890 | A | T | 0.4119 | 0.134 | 0.0176 | 2.4033E-14 | 57.97 |
| rs3735533 | 7 | 27245893 | C | T | 0.9258 | 0.487 | 0.0331 | 6.3227E-49 | 216.47 |
| rs6961048 | 7 | 27328187 | G | C | 0.1038 | 0.2729 | 0.0286 | 1.2779E-21 | 91.05 |
| rs2854746 | 7 | 45960645 | C | G | 0.3997 | 0.113 | 0.018 | 3.257E-10 | 39.41 |
| rs1449596 | 7 | 96395096 | G | C | 0.6455 | 0.1085 | 0.0181 | 1.918E-09 | 35.93 |
| rs13237249 | 7 | 131151783 | T | C | 0.398 | 0.1366 | 0.0177 | 1.0249E-14 | 59.56 |
| rs75511781 | 7 | 131323710 | G | A | 0.0425 | 0.3721 | 0.047 | 2.4519E-15 | 62.68 |
| rs4507656 | 7 | 22156538 | G | C | 0.3066 | 0.1487 | 0.0199 | 8.6896E-14 | 55.84 |
| rs3918226 | 7 | 150690176 | T | C | 0.0813 | 0.6117 | 0.0329 | 5.3064E-77 | 345.69 |
| rs17321041 | 8 | 26445194 | T | C | 0.0633 | 0.2313 | 0.0363 | 1.781E-10 | 40.60 |
| rs2515424 | 8 | 6372965 | T | C | 0.4336 | 0.1278 | 0.0174 | 2.1822E-13 | 53.95 |
| rs73276406 | 8 | 96021760 | C | G | 0.1457 | 0.1564 | 0.0246 | 1.99E-10 | 40.42 |
| rs1906672 | 8 | 38130025 | A | G | 0.2324 | 0.1402 | 0.0205 | 8.4742E-12 | 46.77 |
| rs9918907 | 8 | 124816862 | G | A | 0.2162 | 0.1188 | 0.021 | 1.585E-08 | 32.00 |
| rs62503324 | 8 | 23400615 | T | C | 0.2397 | 0.2033 | 0.0204 | 2.1101E-23 | 99.31 |
| rs6983239 | 8 | 72507296 | T | G | 0.2188 | 0.1159 | 0.0211 | 3.706E-08 | 30.17 |
| rs4909314 | 8 | 135623798 | A | T | 0.3948 | 0.1339 | 0.0177 | 3.4119E-14 | 57.23 |
| rs951914 | 8 | 25878995 | C | G | 0.7126 | 0.1904 | 0.0193 | 5.0583E-23 | 97.32 |
| rs17832905 | 8 | 26038759 | A | C | 0.0717 | 0.1923 | 0.0346 | 2.805E-08 | 30.89 |
| rs4873492 | 8 | 51947549 | T | C | 0.1725 | 0.1401 | 0.0231 | 1.282E-09 | 36.78 |
| rs7012891 | 8 | 126514676 | C | T | 0.2367 | 0.1391 | 0.0205 | 1.1951E-11 | 46.04 |
| rs4615669 | 9 | 21818674 | G | A | 0.4403 | 0.114 | 0.0174 | 6.0954E-11 | 42.93 |
| rs4743021 | 9 | 109414561 | C | T | 0.3147 | 0.108 | 0.0194 | 2.413E-08 | 30.99 |
| rs10759697 | 9 | 117172307 | A | G | 0.4906 | 0.1308 | 0.0173 | 3.9346E-14 | 57.16 |
| rs12337056 | 9 | 628670 | T | C | 0.1761 | 0.1364 | 0.0228 | 2.175E-09 | 35.79 |
| rs10980408 | 9 | 113249071 | C | T | 0.0358 | 0.3745 | 0.0477 | 4.1668E-15 | 61.64 |
| rs2484294 | 10 | 115792062 | A | G | 0.7327 | 0.3165 | 0.0196 | 1.1711E-58 | 260.76 |
| rs11592107 | 10 | 122968964 | A | G | 0.3094 | 0.1203 | 0.0187 | 1.234E-10 | 41.39 |
| rs10490923 | 10 | 124214251 | A | G | 0.1257 | 0.1533 | 0.0262 | 5.021E-09 | 34.24 |
| rs1133400 | 10 | 134459388 | G | A | 0.2148 | 0.1318 | 0.0215 | 8.3041E-10 | 37.58 |
| rs1623474 | 10 | 18471794 | T | C | 0.33 | 0.2234 | 0.0184 | 6.2374E-34 | 147.41 |
| rs3006583 | 10 | 31280845 | C | T | 0.1886 | 0.1303 | 0.0222 | 4.657E-09 | 34.45 |
| rs3802517 | 10 | 28233469 | A | T | 0.4615 | 0.1286 | 0.0173 | 9.2897E-14 | 55.26 |
| rs35506078 | 10 | 65210552 | C | T | 0.3366 | 0.1348 | 0.0183 | 1.536E-13 | 54.26 |
| rs1006545 | 10 | 102553647 | T | G | 0.8875 | 0.3633 | 0.0275 | 7.9634E-40 | 174.53 |
| rs4756779 | 11 | 14225304 | G | A | 0.1758 | 0.1456 | 0.0227 | 1.477E-10 | 41.14 |
| rs10500932 | 11 | 22501446 | A | G | 0.0743 | 0.2784 | 0.0333 | 5.7916E-17 | 69.90 |
| rs7115331 | 11 | 76218590 | G | T | 0.2857 | 0.1266 | 0.0192 | 3.9147E-11 | 43.48 |
| rs604723 | 11 | 100610546 | C | T | 0.7247 | 0.3848 | 0.0194 | 2.3238E-87 | 393.43 |
| rs7938342 | 11 | 1887806 | A | T | 0.5877 | 0.2672 | 0.0181 | 1.924E-49 | 217.93 |
| rs10838702 | 11 | 47410888 | T | G | 0.3875 | 0.2375 | 0.0178 | 1.265E-40 | 178.03 |
| rs11231693 | 11 | 63862612 | A | G | 0.0574 | 0.2223 | 0.0378 | 4.021E-09 | 34.59 |
| rs145422110 | 11 | 1830431 | T | C | 0.0162 | 0.5356 | 0.0745 | 6.4091E-13 | 51.69 |
| rs12363520 | 11 | 10692172 | A | T | 0.229 | 0.1672 | 0.0213 | 4.2355E-15 | 61.62 |
| rs7926335 | 11 | 16917869 | T | C | 0.2699 | 0.1804 | 0.0195 | 2.046E-20 | 85.59 |
| rs7106104 | 11 | 111635655 | C | T | 0.281 | 0.1186 | 0.0193 | 7.7209E-10 | 37.76 |
| rs12574332 | 11 | 122521123 | T | C | 0.1227 | 0.2072 | 0.0266 | 6.1405E-15 | 60.68 |
| rs4936099 | 11 | 130280725 | A | C | 0.5989 | 0.1745 | 0.0178 | 1.1631E-22 | 96.11 |
| rs10832586 | 11 | 16304089 | C | A | 0.2016 | 0.3083 | 0.0216 | 2.5328E-46 | 203.72 |
| rs504217 | 11 | 72006086 | T | C | 0.0736 | 0.2745 | 0.0335 | 2.5061E-16 | 67.14 |
| rs9368 | 12 | 56988342 | A | C | 0.3871 | 0.1121 | 0.0178 | 3.245E-10 | 39.66 |
| rs1732664 | 12 | 79747487 | C | T | 0.6783 | 0.1083 | 0.0194 | 2.581E-08 | 31.16 |
| rs4306343 | 12 | 20190630 | T | A | 0.7212 | 0.317 | 0.0193 | 8.2168E-61 | 269.78 |
| rs520592 | 12 | 69939355 | G | T | 0.1413 | 0.1727 | 0.0248 | 3.514E-12 | 48.49 |
| rs2681485 | 12 | 90025622 | A | G | 0.5976 | 0.2945 | 0.0176 | 1.3131E-62 | 279.99 |
| rs61917655 | 12 | 48210787 | T | C | 0.1011 | 0.2246 | 0.0297 | 3.7162E-14 | 57.19 |
| rs11108209 | 12 | 96109855 | C | T | 0.0932 | 0.1901 | 0.03 | 2.4E-10 | 40.15 |
| rs116063464 | 12 | 109860182 | A | G | 0.0601 | 0.2017 | 0.0369 | 4.679E-08 | 29.88 |
| rs1790123 | 12 | 123659542 | T | C | 0.8032 | 0.1991 | 0.0218 | 6.8707E-20 | 83.41 |
| rs682681 | 13 | 22294062 | C | T | 0.6665 | 0.1454 | 0.0185 | 4.473E-15 | 61.77 |
| rs56256111 | 13 | 41478963 | A | G | 0.1442 | 0.1926 | 0.0263 | 2.599E-13 | 53.63 |
| rs7992292 | 13 | 41968013 | A | G | 0.824 | 0.1367 | 0.0231 | 3.187E-09 | 35.02 |
| rs7321688 | 13 | 115000365 | A | C | 0.2325 | 0.1507 | 0.0205 | 1.9852E-13 | 54.04 |
| rs7324697 | 13 | 58259492 | A | C | 0.325 | 0.1047 | 0.0185 | 1.592E-08 | 32.03 |
| rs1215469 | 13 | 80707408 | C | A | 0.7705 | 0.1383 | 0.0211 | 5.2336E-11 | 42.96 |
| rs12866098 | 13 | 73119617 | A | G | 0.3423 | 0.1033 | 0.0186 | 2.727E-08 | 30.84 |
| rs61948065 | 13 | 25255052 | C | A | 0.1212 | 0.1737 | 0.027 | 1.165E-10 | 41.39 |
| rs3861113 | 13 | 72364382 | A | C | 0.0825 | 0.2126 | 0.0322 | 3.9491E-11 | 43.59 |
| rs36169093 | 13 | 114442187 | A | G | 0.5037 | 0.1231 | 0.0179 | 5.5629E-12 | 47.29 |
| rs227426 | 14 | 70456664 | T | G | 0.5619 | 0.1119 | 0.0175 | 1.745E-10 | 40.89 |
| rs35413927 | 14 | 53420358 | G | A | 0.3049 | 0.1274 | 0.0189 | 1.7681E-11 | 45.44 |
| rs17880989 | 14 | 23313633 | A | G | 0.0259 | 0.4014 | 0.0591 | 1.114E-11 | 46.13 |
| rs2239268 | 14 | 72469591 | A | G | 0.7005 | 0.1097 | 0.019 | 7.3979E-09 | 33.34 |
| rs28429256 | 15 | 66931617 | A | G | 0.3344 | 0.1636 | 0.0188 | 2.8327E-18 | 75.73 |
| rs2627313 | 15 | 81006712 | T | C | 0.4457 | 0.151 | 0.0175 | 5.8546E-18 | 74.45 |
| rs2589218 | 15 | 96785017 | C | T | 0.2698 | 0.1207 | 0.0196 | 6.896E-10 | 37.92 |
| rs11070245 | 15 | 40317792 | G | T | 0.5321 | 0.1287 | 0.0174 | 1.5711E-13 | 54.71 |
| rs4932373 | 15 | 91429287 | C | A | 0.3257 | 0.3664 | 0.0189 | 7.709E-84 | 375.83 |
| rs12906962 | 15 | 95312071 | C | T | 0.3233 | 0.2378 | 0.0188 | 8.7257E-37 | 160.00 |
| rs3743111 | 15 | 71587373 | A | G | 0.613 | 0.1517 | 0.0178 | 1.6181E-17 | 72.63 |
| rs3743369 | 15 | 92707569 | A | G | 0.6278 | 0.104 | 0.0179 | 6.819E-09 | 33.76 |
| rs12596630 | 16 | 2065666 | T | C | 0.0905 | 0.2606 | 0.0314 | 1.0299E-16 | 68.88 |
| rs12929303 | 16 | 81602264 | A | G | 0.5325 | 0.1572 | 0.0174 | 1.5751E-19 | 81.62 |
| rs1049212 | 16 | 4932929 | G | A | 0.5694 | 0.1788 | 0.0175 | 1.2981E-24 | 104.39 |
| rs17696749 | 16 | 75403062 | G | C | 0.5861 | 0.1255 | 0.0176 | 9.4319E-13 | 50.85 |
| rs11859505 | 16 | 74195719 | G | A | 0.5805 | 0.1037 | 0.0181 | 9.756E-09 | 32.82 |
| rs917522 | 16 | 4097222 | T | C | 0.885 | 0.1665 | 0.0273 | 1.038E-09 | 37.20 |
| rs80095680 | 16 | 30902353 | G | A | 0.2633 | 0.1566 | 0.0198 | 2.8113E-15 | 62.55 |
| rs45474499 | 16 | 66914492 | T | C | 0.0473 | 0.3562 | 0.0415 | 8.4996E-18 | 73.67 |
| rs9900637 | 17 | 3951975 | A | C | 0.4961 | 0.0992 | 0.0176 | 1.726E-08 | 31.77 |
| rs138420351 | 17 | 7700063 | T | C | 0.016 | 0.5568 | 0.0854 | 7.1105E-11 | 42.51 |
| rs74439044 | 17 | 7781019 | C | T | 0.0983 | 0.3496 | 0.0294 | 1.382E-32 | 141.40 |
| rs3785837 | 17 | 59468942 | A | G | 0.7635 | 0.1453 | 0.0213 | 9.5675E-12 | 46.53 |
| rs9893005 | 17 | 16225506 | G | C | 0.4647 | 0.1205 | 0.0176 | 7.8959E-12 | 46.88 |
| rs9889262 | 17 | 47398070 | A | T | 0.3666 | 0.2283 | 0.018 | 7.1138E-37 | 160.87 |
| rs76954792 | 17 | 30033514 | T | C | 0.2322 | 0.1213 | 0.0208 | 5.059E-09 | 34.01 |
| rs1867624 | 17 | 62387091 | T | C | 0.6147 | 0.1412 | 0.0178 | 2.0831E-15 | 62.93 |
| rs11661473 | 18 | 42177123 | A | G | 0.2683 | 0.2007 | 0.0196 | 1.5438E-24 | 104.85 |
| rs4890499 | 18 | 42585761 | A | G | 0.2548 | 0.1131 | 0.0199 | 1.328E-08 | 32.30 |
| rs4891258 | 18 | 72995537 | G | A | 0.3174 | 0.1159 | 0.0187 | 5.717E-10 | 38.41 |
| rs10164193 | 18 | 31161426 | G | T | 0.0777 | 0.2196 | 0.0327 | 1.869E-11 | 45.10 |
| rs73036520 | 19 | 45749484 | C | G | 0.2543 | 0.1557 | 0.0202 | 1.34E-14 | 59.41 |
| rs2548459 | 19 | 49209339 | C | T | 0.5195 | 0.132 | 0.0176 | 5.9457E-14 | 56.25 |
| rs7259285 | 19 | 17153149 | A | G | 0.4466 | 0.105 | 0.0176 | 2.646E-09 | 35.59 |
| rs3761077 | 19 | 19325963 | T | G | 0.1106 | 0.1726 | 0.0283 | 1.044E-09 | 37.20 |
| rs7257694 | 19 | 30314666 | T | C | 0.4003 | 0.1837 | 0.0178 | 6.2791E-25 | 106.51 |
| rs1390754 | 19 | 31866105 | T | C | 0.3992 | 0.1258 | 0.0177 | 1.1891E-12 | 50.51 |
| rs318712 | 19 | 11478847 | C | T | 0.0759 | 0.2389 | 0.0334 | 9.1664E-13 | 51.16 |
| rs2291516 | 19 | 11508177 | A | G | 0.1026 | 0.2422 | 0.0292 | 9.881E-17 | 68.80 |
| rs6031431 | 20 | 42795152 | G | A | 0.4622 | 0.1153 | 0.0175 | 4.9408E-11 | 43.41 |
| rs1327235 | 20 | 10969030 | G | A | 0.4714 | 0.3018 | 0.0173 | 4.7643E-68 | 304.33 |
| rs6026739 | 20 | 57739469 | T | A | 0.1226 | 0.5032 | 0.0266 | 1.489E-79 | 357.86 |
| rs6062477 | 20 | 62287600 | T | C | 0.5756 | 0.1191 | 0.0177 | 1.8072E-11 | 45.28 |
| rs35213536 | 20 | 62694319 | T | G | 0.2467 | 0.2044 | 0.0205 | 2.5421E-23 | 99.42 |
| rs7278003 | 21 | 44966069 | C | T | 0.5615 | 0.1293 | 0.0176 | 1.7799E-13 | 53.97 |
| rs12627514 | 21 | 44759440 | G | C | 0.2897 | 0.2164 | 0.0196 | 1.9861E-28 | 121.90 |
| rs2070527 | 21 | 40067495 | C | A | 0.7508 | 0.1473 | 0.0202 | 3.0832E-13 | 53.17 |
| rs1882961 | 21 | 16556367 | T | C | 0.3088 | 0.1272 | 0.0188 | 1.398E-11 | 45.78 |
| rs135023 | 22 | 32442776 | G | A | 0.5784 | 0.1036 | 0.0175 | 3.529E-09 | 35.05 |
| rs5992929 | 22 | 18451977 | T | C | 0.2834 | 0.1684 | 0.0193 | 3.0711E-18 | 76.13 |
| rs786921 | 1 | 89286673 | G | A | 0.5957 | 0.1145 | 0.0176 | 8.6278E-11 | 42.32 |
| rs17396055 | 1 | 94730954 | G | A | 0.3324 | 0.115 | 0.0184 | 4.134E-10 | 39.06 |
| rs882624 | 1 | 201735913 | C | T | 0.3325 | 0.1571 | 0.0185 | 2.334E-17 | 72.11 |
| rs1819663 | 1 | 154025891 | A | G | 0.4929 | 0.1147 | 0.0174 | 4.6249E-11 | 43.45 |
| rs488834 | 1 | 10767902 | C | T | 0.7641 | 0.1931 | 0.0208 | 1.9409E-20 | 86.19 |
| rs12405515 | 1 | 172357441 | G | T | 0.5702 | 0.1698 | 0.0174 | 1.916E-22 | 95.23 |
| rs34645159 | 1 | 1724366 | G | A | 0.5013 | 0.133 | 0.0174 | 2.0721E-14 | 58.43 |
| rs2146315 | 1 | 42050366 | C | T | 0.2318 | 0.1197 | 0.0205 | 5.026E-09 | 34.09 |
| rs1502358 | 1 | 217324932 | G | A | 0.6813 | 0.1127 | 0.0185 | 1.13E-09 | 37.11 |
| rs55857306 | 1 | 11895795 | G | A | 0.1602 | 0.5224 | 0.0235 | 5.047E-109 | 494.16 |
| rs4926923 | 1 | 48109225 | T | C | 0.0883 | 0.1918 | 0.0308 | 4.749E-10 | 38.78 |
| rs34517439 | 1 | 78450517 | C | A | 0.1199 | 0.2514 | 0.0279 | 2.024E-19 | 81.19 |
| rs2160236 | 2 | 40557276 | G | C | 0.3792 | 0.1421 | 0.0181 | 4.3092E-15 | 61.64 |
| rs2421200 | 2 | 61711815 | G | T | 0.4882 | 0.1097 | 0.0173 | 2.587E-10 | 40.21 |
| rs311564 | 2 | 86293498 | G | A | 0.3461 | 0.133 | 0.0183 | 4.2345E-13 | 52.82 |
| rs62158170 | 2 | 114082175 | A | G | 0.2166 | 0.1645 | 0.0211 | 6.6329E-15 | 60.78 |
| rs6715901 | 2 | 179650954 | G | A | 0.4962 | 0.1377 | 0.0174 | 2.7599E-15 | 62.63 |
| rs10804330 | 2 | 227185749 | T | C | 0.4329 | 0.1331 | 0.0176 | 4.6015E-14 | 57.19 |
| rs112393817 | 2 | 9807226 | C | G | 0.2174 | 0.116 | 0.0211 | 3.799E-08 | 30.22 |
| rs4952668 | 2 | 43386568 | G | A | 0.6237 | 0.192 | 0.018 | 1.129E-26 | 113.78 |
| rs1035673 | 2 | 218675533 | T | C | 0.6032 | 0.1625 | 0.0176 | 2.995E-20 | 85.25 |
| rs1039897 | 2 | 220337196 | G | A | 0.6503 | 0.1085 | 0.0183 | 3.264E-09 | 35.15 |
| rs1275985 | 2 | 26911745 | C | T | 0.6133 | 0.2943 | 0.0177 | 2.7682E-62 | 276.46 |
| rs4954192 | 2 | 135632981 | C | T | 0.3872 | 0.1225 | 0.0179 | 8.1452E-12 | 46.83 |
| rs7576060 | 2 | 188073163 | C | T | 0.35 | 0.1017 | 0.0181 | 2.052E-08 | 31.57 |
| rs11692619 | 2 | 205084439 | C | T | 0.3607 | 0.1281 | 0.0184 | 3.3144E-12 | 48.47 |
| rs13004222 | 2 | 219560492 | C | G | 0.051 | 0.2944 | 0.0393 | 7.1138E-14 | 56.12 |
| rs1044822 | 2 | 230629138 | C | T | 0.1488 | 0.1334 | 0.0243 | 4.135E-08 | 30.14 |
| rs76326501 | 2 | 43167878 | A | C | 0.0911 | 0.3618 | 0.0305 | 2.1742E-32 | 140.71 |
| rs28377357 | 2 | 112769721 | G | A | 0.2938 | 0.1243 | 0.019 | 6.027E-11 | 42.80 |
| rs6735275 | 2 | 174013819 | T | C | 0.2709 | 0.1225 | 0.0194 | 2.619E-10 | 39.87 |
| rs12693302 | 2 | 183211443 | G | A | 0.6518 | 0.2378 | 0.0181 | 2.1548E-39 | 172.61 |
| rs1687295 | 3 | 14889756 | T | C | 0.7296 | 0.2061 | 0.0194 | 2.9923E-26 | 112.86 |
| rs7427249 | 3 | 37572489 | G | A | 0.58 | 0.1098 | 0.0176 | 4.336E-10 | 38.92 |
| rs6795735 | 3 | 64705365 | C | T | 0.4109 | 0.1438 | 0.0176 | 3.0542E-16 | 66.76 |
| rs9289557 | 3 | 138071604 | C | T | 0.2604 | 0.119 | 0.0207 | 8.681E-09 | 33.05 |
| rs16853198 | 3 | 168840179 | A | G | 0.0762 | 0.3386 | 0.0327 | 4.4371E-25 | 107.22 |
| rs147501096 | 3 | 186180253 | G | C | 0.072 | 0.1955 | 0.0341 | 9.936E-09 | 32.87 |
| rs4244200 | 3 | 196226059 | G | C | 0.2799 | 0.1215 | 0.0193 | 3.233E-10 | 39.63 |
| rs28675079 | 3 | 111500002 | G | A | 0.1867 | 0.1444 | 0.0222 | 8.3426E-11 | 42.31 |
| rs4141663 | 3 | 124551967 | C | T | 0.4216 | 0.1496 | 0.0175 | 1.407E-17 | 73.08 |
| rs1527797 | 3 | 153860652 | C | T | 0.7395 | 0.1409 | 0.0197 | 8.6796E-13 | 51.16 |
| rs7623706 | 3 | 74712754 | A | G | 0.4349 | 0.0975 | 0.0176 | 2.835E-08 | 30.69 |
| rs7611674 | 3 | 179169230 | T | G | 0.1962 | 0.1576 | 0.0223 | 1.6742E-12 | 49.95 |
| rs3772219 | 3 | 56771251 | A | C | 0.3193 | 0.1754 | 0.0185 | 2.9383E-21 | 89.89 |
| rs78809139 | 3 | 154674943 | G | A | 0.1014 | 0.2281 | 0.0288 | 2.5823E-15 | 62.73 |
| rs1528293 | 3 | 169154511 | A | T | 0.5079 | 0.2764 | 0.0173 | 1.4771E-57 | 255.26 |
| rs11721984 | 4 | 38343935 | C | T | 0.4532 | 0.1409 | 0.0177 | 1.8858E-15 | 63.37 |
| rs13107325 | 4 | 103188709 | C | T | 0.0742 | 0.6747 | 0.0339 | 3.7214E-88 | 396.12 |
| rs13139571 | 4 | 156645513 | C | A | 0.2366 | 0.2408 | 0.0203 | 2.2919E-32 | 140.71 |
| rs1425486 | 4 | 157683685 | C | T | 0.3207 | 0.1331 | 0.0187 | 1.1069E-12 | 50.66 |
| rs16896276 | 4 | 18015156 | T | A | 0.2625 | 0.1309 | 0.0198 | 3.8151E-11 | 43.71 |
| rs11945489 | 4 | 56463775 | C | T | 0.2909 | 0.1392 | 0.0192 | 3.993E-13 | 52.56 |
| rs12503341 | 4 | 106925311 | G | A | 0.0394 | 0.2993 | 0.0462 | 9.4298E-11 | 41.97 |
| rs13152154 | 4 | 77417756 | C | T | 0.7293 | 0.1186 | 0.0195 | 1.226E-09 | 36.99 |
| rs13118687 | 4 | 111406496 | G | A | 0.4702 | 0.1496 | 0.0175 | 1.3662E-17 | 73.08 |
| rs11745207 | 5 | 131856033 | C | G | 0.2579 | 0.1132 | 0.0199 | 1.288E-08 | 32.36 |
| rs11960210 | 5 | 157817634 | T | C | 0.3751 | 0.2474 | 0.018 | 3.3628E-43 | 188.91 |
| rs78909293 | 5 | 148335250 | T | C | 0.0449 | 0.321 | 0.0429 | 7.3063E-14 | 55.99 |
| rs114503346 | 5 | 172192350 | C | T | 0.0461 | 0.2678 | 0.0426 | 3.095E-10 | 39.52 |
| rs9326869 | 5 | 112349070 | T | C | 0.7513 | 0.1096 | 0.02 | 3.986E-08 | 30.03 |
| rs335170 | 5 | 122482243 | A | C | 0.5923 | 0.113 | 0.0177 | 1.607E-10 | 40.76 |
| rs4645335 | 5 | 3704761 | A | G | 0.664 | 0.1142 | 0.0185 | 7.0361E-10 | 38.11 |
| rs6875967 | 5 | 50878292 | A | G | 0.6479 | 0.1344 | 0.0181 | 1.214E-13 | 55.14 |
| rs62378003 | 5 | 89514206 | C | T | 0.1141 | 0.1714 | 0.028 | 8.9039E-10 | 37.47 |
| rs55770741 | 5 | 96220087 | C | T | 0.5613 | 0.1281 | 0.0175 | 2.2019E-13 | 53.58 |
| rs1467049 | 5 | 42440062 | T | G | 0.1949 | 0.1244 | 0.0219 | 1.35E-08 | 32.27 |
| rs6556384 | 5 | 158418952 | C | A | 0.8105 | 0.152 | 0.0221 | 5.9075E-12 | 47.30 |
| rs55993676 | 5 | 173303392 | G | T | 0.2916 | 0.2097 | 0.0191 | 3.8186E-28 | 120.54 |
| rs2569882 | 6 | 1620147 | T | C | 0.4342 | 0.1199 | 0.0182 | 4.2796E-11 | 43.40 |
| rs62413546 | 6 | 56012664 | C | T | 0.0847 | 0.1877 | 0.032 | 4.583E-09 | 34.41 |
| rs504691 | 6 | 72206620 | C | A | 0.4002 | 0.1177 | 0.0177 | 3.1383E-11 | 44.22 |
| rs11153730 | 6 | 118667522 | T | C | 0.4906 | 0.1551 | 0.0173 | 2.568E-19 | 80.38 |
| rs636202 | 6 | 139843583 | T | C | 0.5185 | 0.1023 | 0.0174 | 4.399E-09 | 34.57 |
| rs9365555 | 6 | 163757127 | A | G | 0.3259 | 0.1254 | 0.0187 | 1.9638E-11 | 44.97 |
| rs11961593 | 6 | 166164137 | C | T | 0.0685 | 0.3158 | 0.0349 | 1.4931E-19 | 81.88 |
| rs2744133 | 6 | 22392260 | A | G | 0.2749 | 0.1435 | 0.0193 | 1.17E-13 | 55.28 |
| rs62434124 | 6 | 150999751 | C | T | 0.0711 | 0.4853 | 0.0338 | 7.8343E-47 | 206.15 |
| rs1322639 | 6 | 169587103 | G | A | 0.7766 | 0.1584 | 0.0209 | 3.8726E-14 | 57.44 |
| rs1265157 | 6 | 31142265 | C | G | 0.3521 | 0.1444 | 0.0187 | 1.176E-14 | 59.63 |
| rs9399137 | 6 | 135419018 | T | C | 0.2619 | 0.1148 | 0.0197 | 5.831E-09 | 33.96 |
| rs9478282 | 6 | 152398669 | C | T | 0.1116 | 0.1994 | 0.0279 | 8.7036E-13 | 51.08 |
| rs5012479 | 6 | 109630096 | T | G | 0.5205 | 0.0956 | 0.0174 | 3.657E-08 | 30.19 |
| rs11153590 | 6 | 116322381 | G | A | 0.3831 | 0.1145 | 0.0178 | 1.125E-10 | 41.38 |
| rs1178979 | 7 | 72856430 | T | C | 0.1953 | 0.1504 | 0.0221 | 9.9586E-12 | 46.31 |
| rs3807101 | 7 | 80393418 | C | T | 0.123 | 0.1743 | 0.0265 | 4.5698E-11 | 43.26 |
| rs1534338 | 7 | 156315173 | G | A | 0.6029 | 0.1144 | 0.0178 | 1.236E-10 | 41.31 |
| rs73033340 | 7 | 1195692 | A | G | 0.0362 | 0.5312 | 0.0525 | 5.0606E-24 | 102.38 |
| rs342977 | 7 | 35459888 | G | A | 0.7715 | 0.1577 | 0.0205 | 1.6742E-14 | 59.18 |
| rs17454517 | 7 | 50915776 | A | G | 0.5064 | 0.1216 | 0.0174 | 2.6522E-12 | 48.84 |
| rs11556924 | 7 | 129663496 | C | T | 0.3827 | 0.181 | 0.0181 | 1.8311E-23 | 100.00 |
| rs2906152 | 7 | 2523003 | G | A | 0.6304 | 0.1873 | 0.0181 | 5.5475E-25 | 107.08 |
| rs7788746 | 7 | 99612405 | G | T | 0.6691 | 0.1644 | 0.0183 | 3.193E-19 | 80.71 |
| rs4556017 | 7 | 100632790 | C | T | 0.8524 | 0.1601 | 0.0247 | 9.665E-11 | 42.01 |
| rs2191046 | 7 | 107834075 | T | G | 0.2646 | 0.1184 | 0.0197 | 1.775E-09 | 36.12 |
| rs7800558 | 7 | 140242661 | T | C | 0.4219 | 0.096 | 0.0175 | 4.459E-08 | 30.09 |
| rs11778153 | 8 | 64503942 | T | C | 0.3569 | 0.1192 | 0.0182 | 5.8425E-11 | 42.90 |
| rs1693560 | 8 | 101680292 | A | G | 0.4603 | 0.1525 | 0.0175 | 3.4104E-18 | 75.94 |
| rs142449193 | 8 | 102750597 | C | T | 0.046 | 0.2573 | 0.0426 | 1.506E-09 | 36.48 |
| rs2957468 | 8 | 106325360 | A | G | 0.6646 | 0.1377 | 0.0185 | 8.4314E-14 | 55.40 |
| rs722783 | 8 | 120442287 | G | A | 0.2216 | 0.2093 | 0.0208 | 9.0261E-24 | 101.25 |
| rs35091929 | 8 | 10693492 | T | C | 0.6032 | 0.1828 | 0.0177 | 6.461E-25 | 106.66 |
| rs148401029 | 8 | 81386066 | C | A | 0.0352 | 0.3122 | 0.0486 | 1.321E-10 | 41.27 |
| rs4739832 | 8 | 82812019 | A | C | 0.4167 | 0.1323 | 0.0176 | 5.8117E-14 | 56.51 |
| rs10087280 | 8 | 49391836 | A | G | 0.1683 | 0.1381 | 0.0232 | 2.538E-09 | 35.43 |
| rs4074812 | 8 | 141883529 | G | A | 0.5535 | 0.1336 | 0.0175 | 2.0701E-14 | 58.28 |
| rs3802230 | 8 | 143992864 | C | A | 0.5446 | 0.1605 | 0.0174 | 2.7517E-20 | 85.08 |
| rs2133386 | 9 | 128173838 | C | A | 0.4327 | 0.1322 | 0.0176 | 5.2144E-14 | 56.42 |
| rs6271 | 9 | 136522274 | C | T | 0.0737 | 0.4313 | 0.0352 | 1.7179E-34 | 150.13 |
| rs10491713 | 9 | 2506236 | G | T | 0.1977 | 0.1219 | 0.0217 | 2.046E-08 | 31.56 |
| rs1243876 | 9 | 35693104 | C | T | 0.7012 | 0.1063 | 0.019 | 2.142E-08 | 31.30 |
| rs76452347 | 9 | 35906471 | C | T | 0.2053 | 0.2246 | 0.0229 | 9.3713E-23 | 96.19 |
| rs507666 | 9 | 136149399 | G | A | 0.1872 | 0.2854 | 0.0223 | 2.2667E-37 | 163.79 |
| rs11145807 | 9 | 139520789 | A | G | 0.5942 | 0.155 | 0.0184 | 4.1039E-17 | 70.96 |
| rs1332812 | 9 | 9350986 | T | A | 0.6469 | 0.1145 | 0.0181 | 2.708E-10 | 40.02 |
| rs11141731 | 9 | 89888472 | C | T | 0.228 | 0.1258 | 0.0207 | 1.308E-09 | 36.93 |
| rs2236295 | 10 | 64564892 | G | T | 0.3992 | 0.207 | 0.0177 | 1.4191E-31 | 136.77 |
| rs12247028 | 10 | 75410052 | G | A | 0.6322 | 0.1396 | 0.0188 | 1.1841E-13 | 55.14 |
| rs6602177 | 10 | 17167141 | C | T | 0.7073 | 0.1203 | 0.0207 | 6.5209E-09 | 33.77 |
| rs12258967 | 10 | 18727959 | C | G | 0.2958 | 0.354 | 0.0193 | 3.2719E-75 | 336.43 |
| rs2067831 | 10 | 105643223 | G | C | 0.2723 | 0.128 | 0.0195 | 5.0769E-11 | 43.09 |
| rs4284362 | 10 | 45377839 | C | A | 0.7181 | 0.1586 | 0.0194 | 3.2412E-16 | 66.83 |
| rs11187838 | 10 | 96038686 | G | A | 0.4321 | 0.2781 | 0.0174 | 2.5539E-57 | 255.45 |
| rs11191580 | 10 | 104906211 | T | C | 0.0821 | 0.5071 | 0.0316 | 6.6329E-58 | 257.52 |
| rs11252324 | 10 | 4124568 | G | T | 0.077 | 0.2339 | 0.0328 | 1.029E-12 | 50.85 |
| rs34130368 | 10 | 48411796 | G | T | 0.1172 | 0.2027 | 0.0284 | 8.772E-13 | 50.94 |
| rs9419374 | 10 | 133729749 | A | G | 0.646 | 0.1164 | 0.0185 | 3.442E-10 | 39.59 |
| rs1265842 | 10 | 28924901 | T | C | 0.5166 | 0.1113 | 0.0174 | 1.703E-10 | 40.92 |
| rs2487926 | 10 | 30300787 | A | G | 0.4295 | 0.0972 | 0.0176 | 3.313E-08 | 30.50 |
| rs72831343 | 10 | 63515681 | T | G | 0.1419 | 0.4936 | 0.0248 | 4.7687E-88 | 396.14 |
| rs72842207 | 10 | 121433675 | C | T | 0.2149 | 0.2112 | 0.0211 | 1.1E-23 | 100.19 |
| rs11245631 | 11 | 50279561 | C | T | 0.1955 | 0.1474 | 0.0223 | 3.9949E-11 | 43.69 |
| rs11021221 | 11 | 95308854 | T | A | 0.1668 | 0.1877 | 0.0233 | 6.9311E-16 | 64.90 |
| rs79889784 | 11 | 1702117 | G | T | 0.0176 | 0.3941 | 0.0717 | 3.862E-08 | 30.21 |
| rs962369 | 11 | 27734420 | T | C | 0.3013 | 0.1684 | 0.0189 | 6.0228E-19 | 79.39 |
| rs7933758 | 11 | 31000774 | C | T | 0.3047 | 0.1138 | 0.0191 | 2.58E-09 | 35.50 |
| rs4930295 | 11 | 65390554 | C | G | 0.2216 | 0.2418 | 0.0209 | 5.5424E-31 | 133.85 |
| rs61909958 | 11 | 96151677 | C | G | 0.1881 | 0.1275 | 0.0228 | 2.213E-08 | 31.27 |
| rs66682451 | 11 | 107097540 | A | G | 0.2747 | 0.1348 | 0.0194 | 3.4364E-12 | 48.28 |
| rs12790943 | 11 | 120058623 | C | T | 0.4213 | 0.1002 | 0.0175 | 1.135E-08 | 32.78 |
| rs751984 | 11 | 61278246 | T | C | 0.1174 | 0.3937 | 0.0275 | 1.3778E-46 | 204.96 |
| rs7137828 | 12 | 111932800 | C | T | 0.5183 | 0.5027 | 0.0176 | 4.797E-180 | 815.82 |
| rs61912333 | 12 | 19554817 | C | G | 0.5037 | 0.1191 | 0.0176 | 1.1311E-11 | 45.79 |
| rs6487076 | 12 | 20470857 | A | G | 0.223 | 0.174 | 0.0209 | 8.6856E-17 | 69.31 |
| rs11112548 | 12 | 105871914 | A | T | 0.0444 | 0.2742 | 0.0443 | 5.802E-10 | 38.31 |
| rs6580970 | 12 | 54434277 | C | T | 0.2987 | 0.1661 | 0.0191 | 4.0281E-18 | 75.63 |
| rs75507123 | 12 | 5417856 | G | T | 0.1274 | 0.1434 | 0.0261 | 3.942E-08 | 30.19 |
| rs1948151 | 12 | 26459071 | G | A | 0.2777 | 0.1355 | 0.0193 | 2.1189E-12 | 49.29 |
| rs1669907 | 12 | 42777933 | T | G | 0.6968 | 0.1158 | 0.0191 | 1.356E-09 | 36.76 |
| rs7967705 | 12 | 50511408 | T | C | 0.6196 | 0.2694 | 0.0178 | 1.5399E-51 | 229.06 |
| rs7959649 | 12 | 67783108 | T | C | 0.7576 | 0.1166 | 0.0202 | 8.1429E-09 | 33.32 |
| rs710698 | 12 | 70369918 | A | G | 0.4135 | 0.1059 | 0.0176 | 1.885E-09 | 36.20 |
| rs35443 | 12 | 115552878 | G | C | 0.3858 | 0.2661 | 0.0178 | 1.1959E-50 | 223.49 |
| rs2271139 | 12 | 124839540 | C | A | 0.286 | 0.1247 | 0.0192 | 8.2262E-11 | 42.18 |
| rs544012 | 13 | 110878639 | T | G | 0.7339 | 0.1143 | 0.0201 | 1.375E-08 | 32.34 |
| rs9526707 | 13 | 51489186 | G | A | 0.3222 | 0.1217 | 0.0186 | 6.5902E-11 | 42.81 |
| rs55684003 | 13 | 97988689 | A | G | 0.3041 | 0.122 | 0.0189 | 1.014E-10 | 41.67 |
| rs9508495 | 13 | 30146201 | C | T | 0.7569 | 0.1944 | 0.0204 | 1.3409E-21 | 90.81 |
| rs4424827 | 14 | 35110857 | C | T | 0.5669 | 0.0981 | 0.0175 | 2.111E-08 | 31.42 |
| rs7350752 | 14 | 21841154 | G | A | 0.1241 | 0.1504 | 0.0268 | 1.966E-08 | 31.49 |
| rs1950500 | 14 | 24830850 | T | C | 0.7081 | 0.1396 | 0.019 | 2.2029E-13 | 53.98 |
| rs7155504 | 14 | 36158828 | T | C | 0.0876 | 0.2286 | 0.0317 | 5.1618E-13 | 52.00 |
| rs194742 | 14 | 69287483 | T | C | 0.831 | 0.1281 | 0.0232 | 3.173E-08 | 30.49 |
| rs72683923 | 14 | 50735947 | T | C | 0.0212 | 0.5325 | 0.0635 | 5.0234E-17 | 70.32 |
| rs4903064 | 14 | 73279420 | T | C | 0.2355 | 0.1543 | 0.0206 | 7.8433E-14 | 56.10 |
| rs8014182 | 14 | 103859962 | C | T | 0.1319 | 0.1942 | 0.0257 | 3.9382E-14 | 57.10 |
| rs11636952 | 15 | 75114322 | T | C | 0.6869 | 0.3997 | 0.0189 | 5.21E-99 | 447.24 |
| rs2925345 | 15 | 41311799 | T | C | 0.5324 | 0.189 | 0.0174 | 1.5951E-27 | 117.98 |
| rs10873612 | 15 | 26105602 | C | T | 0.5961 | 0.1096 | 0.0179 | 9.5061E-10 | 37.49 |
| rs2469141 | 15 | 66967398 | T | C | 0.1628 | 0.1351 | 0.0238 | 1.391E-08 | 32.22 |
| rs57708073 | 15 | 79066653 | A | G | 0.2608 | 0.1907 | 0.0214 | 4.7261E-19 | 79.41 |
| rs9932220 | 16 | 51758116 | G | A | 0.2177 | 0.1591 | 0.021 | 3.7558E-14 | 57.40 |
| rs908951 | 16 | 89697625 | C | T | 0.437 | 0.1983 | 0.0181 | 7.7322E-28 | 120.03 |
| rs9937801 | 16 | 21088130 | T | C | 0.4308 | 0.1554 | 0.0174 | 4.8095E-19 | 79.76 |
| rs7192407 | 16 | 49783926 | T | C | 0.528 | 0.1019 | 0.0174 | 4.534E-09 | 34.30 |
| rs62030049 | 16 | 50572709 | A | G | 0.2404 | 0.1336 | 0.0209 | 1.549E-10 | 40.86 |
| rs12149254 | 16 | 71458851 | G | A | 0.1697 | 0.1324 | 0.0232 | 1.142E-08 | 32.57 |
| rs79286081 | 16 | 86555837 | G | A | 0.1021 | 0.1631 | 0.0299 | 4.83E-08 | 29.76 |
| rs77924615 | 16 | 20392332 | G | A | 0.1982 | 0.3163 | 0.0224 | 3.7239E-45 | 199.39 |
| rs12919839 | 16 | 56859216 | C | T | 0.2841 | 0.1098 | 0.0192 | 1.044E-08 | 32.70 |
| rs28544928 | 16 | 69329268 | T | G | 0.2535 | 0.1543 | 0.0199 | 9.1306E-15 | 60.12 |
| rs4362428 | 17 | 2090341 | C | A | 0.4087 | 0.1127 | 0.0176 | 1.452E-10 | 41.00 |
| rs79724577 | 17 | 43463493 | A | C | 0.1832 | 0.1362 | 0.023 | 3.471E-09 | 35.07 |
| rs3916033 | 17 | 44889703 | C | T | 0.5565 | 0.1233 | 0.0185 | 2.4149E-11 | 44.42 |
| rs4295 | 17 | 61556298 | C | G | 0.6202 | 0.1802 | 0.018 | 1.7061E-23 | 100.22 |
| rs7217916 | 17 | 76769434 | A | G | 0.6146 | 0.1111 | 0.0179 | 5.633E-10 | 38.52 |
| rs28661492 | 17 | 30609932 | C | T | 0.2022 | 0.1359 | 0.0222 | 9.5609E-10 | 37.47 |
| rs11077961 | 17 | 81012749 | A | G | 0.3676 | 0.1073 | 0.0186 | 8.5491E-09 | 33.28 |
| rs2239917 | 17 | 43165887 | T | C | 0.5748 | 0.1731 | 0.0176 | 9.685E-23 | 96.73 |
| rs8078510 | 17 | 47045862 | G | A | 0.2693 | 0.1277 | 0.0197 | 9.8424E-11 | 42.02 |
| rs1436138 | 17 | 75316880 | A | G | 0.3633 | 0.1991 | 0.0182 | 7.3266E-28 | 119.67 |
| rs58693787 | 18 | 48141710 | A | G | 0.2458 | 0.1584 | 0.0202 | 3.8159E-15 | 61.49 |
| rs11665020 | 18 | 10879503 | G | C | 0.322 | 0.1423 | 0.0187 | 2.7772E-14 | 57.91 |
| rs10048404 | 18 | 54578482 | C | T | 0.3694 | 0.1096 | 0.0183 | 2.005E-09 | 35.87 |
| rs7235890 | 18 | 55732115 | G | T | 0.8956 | 0.1692 | 0.0288 | 4.121E-09 | 34.52 |
| rs34413141 | 18 | 777282 | T | A | 0.1821 | 0.1808 | 0.0227 | 1.4911E-15 | 63.44 |
| rs1903752 | 18 | 7129327 | C | T | 0.5386 | 0.0987 | 0.0178 | 3.198E-08 | 30.75 |
| rs11664194 | 18 | 20021031 | T | A | 0.4603 | 0.1078 | 0.0176 | 8.69E-10 | 37.52 |
| rs12609484 | 19 | 4970593 | G | T | 0.3163 | 0.1398 | 0.0188 | 1.1641E-13 | 55.30 |
| rs73046792 | 19 | 49605705 | G | A | 0.1592 | 0.1518 | 0.0245 | 5.8721E-10 | 38.39 |
| rs12978472 | 19 | 7257990 | C | G | 0.1247 | 0.4779 | 0.0281 | 8.4586E-65 | 289.24 |
| rs1433121 | 19 | 32591878 | C | T | 0.6906 | 0.1352 | 0.0188 | 6.9072E-13 | 51.72 |
| rs6108168 | 20 | 8626271 | C | A | 0.2546 | 0.1901 | 0.0199 | 1.1031E-21 | 91.26 |
| rs672272 | 20 | 10488159 | C | T | 0.6041 | 0.1851 | 0.0178 | 2.772E-25 | 108.14 |
| rs6058261 | 20 | 30235470 | C | A | 0.2736 | 0.1201 | 0.0195 | 6.83E-10 | 37.93 |
| rs34587839 | 20 | 32300671 | G | A | 0.1535 | 0.1669 | 0.0244 | 8.2224E-12 | 46.79 |
| rs4814837 | 20 | 19241680 | C | T | 0.3424 | 0.1003 | 0.0184 | 4.617E-08 | 29.71 |
| rs6078393 | 20 | 11908101 | T | G | 0.4106 | 0.1205 | 0.0176 | 7.6577E-12 | 46.88 |
| rs234616 | 20 | 57494858 | G | A | 0.3112 | 0.1152 | 0.0188 | 8.7941E-10 | 37.55 |
| rs7265695 | 20 | 40043096 | T | C | 0.1965 | 0.1967 | 0.0219 | 2.482E-19 | 80.67 |
| rs2598 | 20 | 47241618 | A | G | 0.4674 | 0.1387 | 0.0175 | 1.9351E-15 | 62.82 |
| rs79044887 | 20 | 47427831 | C | G | 0.1476 | 0.2427 | 0.0245 | 4.0059E-23 | 98.13 |
| rs34487963 | 21 | 44838330 | C | A | 0.0185 | 0.5734 | 0.0712 | 8.179E-16 | 64.86 |
| rs12321 | 22 | 29453193 | G | C | 0.4333 | 0.1492 | 0.0175 | 1.4381E-17 | 72.69 |
| rs926335 | 22 | 28046423 | C | T | 0.4764 | 0.1215 | 0.0175 | 3.3721E-12 | 48.20 |

SNP, single nucleotide polymorphism; Chr, chromosome; Pos, position (hg19); EA, effect allele; OA, other allele; EAF, effect allele frequency; SE: standard error.

**Supplementary Table 5. SNPs related to systolic BP in genes that are the target of antihypertensive drugs in one-sample Mendelian randomization analysis.**

| **Drug** | **SNP** | **Chr** | **Pos** | **EA** | **OA** | **EAF** | **Beta** | **SE** | **P value** |
| --- | --- | --- | --- | --- | --- | --- | --- | --- | --- |
| ACEi | rs8077276 | 17 | 61547562 | A | G | 0.6201 | 0.295 | 0.03 | 5.145E-21 |
| BB | rs740956 | 7 | 150598440 | C | T | 0.4331 | 0.2 | 0.03 | 5.211E-11 |
| BB | rs3800787 | 7 | 150713636 | C | G | 0.377 | 0.189 | 0.03 | 7.644E-09 |
| BB | rs2853792 | 7 | 150699877 | A | G | 0.6074 | 0.201 | 0.03 | 3.044E-10 |
| BB | rs891511 | 7 | 150704843 | A | G | 0.3335 | 0.351 | 0.03 | 6.132E-26 |
| BB | rs855715 | 10 | 115823524 | T | G | 0.1202 | 0.439 | 0.05 | 7.878E-20 |
| BB | rs79850079 | 10 | 115790006 | A | G | 0.0317 | 0.58 | 0.09 | 1.448E-10 |
| BB | rs68122733 | 10 | 115831533 | G | A | 0.1718 | 0.334 | 0.04 | 1.688E-16 |
| BB | rs180898 | 10 | 115765397 | C | A | 0.0661 | 0.518 | 0.07 | 1.647E-15 |
| BB | rs12540183 | 7 | 150664141 | C | T | 0.3843 | 0.195 | 0.03 | 4.17E-10 |
| BB | rs117564322 | 7 | 150684021 | G | A | 0.031 | 0.739 | 0.09 | 1.9E-15 |
| BB | rs3918226 | 7 | 150690176 | C | T | 0.0811 | 0.664 | 0.06 | 8.461E-31 |
| BB | rs740746 | 10 | 115792787 | G | A | 0.7318 | 0.456 | 0.03 | 1.421E-40 |
| BB | rs2429511 | 10 | 115801253 | T | C | 0.4799 | 0.373 | 0.03 | 7.386E-35 |
| BB | rs143854972 | 10 | 115843445 | G | A | 0.0578 | 0.431 | 0.07 | 8.894E-11 |
| BB | rs11196553 | 10 | 115710997 | C | T | 0.045 | 0.625 | 0.07 | 2.894E-17 |
| BB | rs460718 | 10 | 115721364 | A | G | 0.6734 | 0.276 | 0.03 | 1.358E-17 |
| BB | rs2782980 | 10 | 115781527 | T | C | 0.7159 | 0.425 | 0.03 | 3.575E-36 |
| BB | rs17875473 | 10 | 115800294 | C | T | 0.0871 | 0.328 | 0.06 | 2.658E-09 |
| BB | rs11196597 | 10 | 115788094 | G | A | 0.133 | 0.286 | 0.05 | 4.227E-10 |
| BB | rs11196625 | 10 | 115843990 | G | A | 0.1928 | 0.227 | 0.04 | 8.286E-09 |
| CCB | rs113210396 | 3 | 53612327 | T | G | 0.0451 | 0.434 | 0.08 | 1.76E-08 |
| CCB | rs62250937 | 3 | 53870318 | C | T | 0.0893 | 0.324 | 0.06 | 7.279E-09 |
| CCB | rs312487 | 3 | 53545622 | C | T | 0.5217 | 0.219 | 0.03 | 9.647E-13 |
| CCB | rs1757213 | 10 | 18537594 | G | A | 0.888 | 0.308 | 0.05 | 1.152E-09 |
| CCB | rs72786098 | 10 | 18729855 | A | G | 0.0322 | 0.503 | 0.09 | 1.18E-08 |
| CCB | rs17610275 | 10 | 18621630 | G | T | 0.0734 | 0.387 | 0.06 | 2.87E-10 |
| CCB | rs10828689 | 10 | 18721957 | C | G | 0.4438 | 0.363 | 0.03 | 6.942E-33 |
| CCB | rs10828749 | 10 | 18756881 | A | G | 0.412 | 0.366 | 0.03 | 2.268E-32 |
| CCB | rs75699707 | 10 | 18359294 | A | G | 0.0264 | 0.576 | 0.1 | 2.103E-08 |
| CCB | rs4748444 | 10 | 18494482 | C | T | 0.3363 | 0.194 | 0.03 | 3.126E-09 |
| CCB | rs10828662 | 10 | 18703097 | T | C | 0.5586 | 0.288 | 0.03 | 2.541E-21 |
| CCB | rs982003 | 10 | 18707296 | T | C | 0.7568 | 0.241 | 0.04 | 6.209E-12 |
| CCB | rs7076247 | 10 | 18759629 | C | T | 0.6114 | 0.256 | 0.03 | 1.334E-16 |
| CCB | rs10828399 | 10 | 18553968 | A | G | 0.5218 | 0.195 | 0.03 | 1.102E-10 |
| CCB | rs11013938 | 10 | 18669271 | C | G | 0.2554 | 0.327 | 0.04 | 1.166E-20 |
| CCB | rs12258967 | 10 | 18727959 | G | C | 0.2953 | 0.633 | 0.03 | 1.083E-78 |
| CCB | rs4748438 | 10 | 18343319 | A | G | 0.4603 | 0.174 | 0.03 | 1.114E-08 |
| CCB | rs7917532 | 10 | 18373902 | C | T | 0.5347 | 0.232 | 0.03 | 2.011E-14 |
| CCB | rs11014170 | 10 | 18710991 | A | G | 0.0206 | 0.67 | 0.12 | 5.613E-09 |
| CCB | rs67214975 | 10 | 18727251 | A | C | 0.4563 | 0.414 | 0.03 | 1.416E-41 |
| CCB | rs10828784 | 10 | 18788273 | G | C | 0.3367 | 0.202 | 0.03 | 4.486E-09 |
| CCB | rs12416052 | 10 | 18789267 | C | T | 0.4053 | 0.199 | 0.03 | 1.591E-10 |
| CCB | rs1779241 | 10 | 18476322 | G | A | 0.9024 | 0.382 | 0.05 | 6.279E-14 |
| CCB | rs10828452 | 10 | 18592450 | T | A | 0.207 | 0.305 | 0.04 | 4.198E-15 |
| CCB | rs10828542 | 10 | 18627285 | G | A | 0.3863 | 0.182 | 0.03 | 5.178E-09 |
| CCB | rs112701401 | 10 | 18644811 | G | C | 0.0305 | 0.503 | 0.09 | 4.915E-08 |
| CCB | rs112133583 | 10 | 18695681 | T | C | 0.0299 | 0.555 | 0.1 | 1.181E-08 |
| CCB | rs72786085 | 10 | 18713206 | C | G | 0.0792 | 0.531 | 0.06 | 4.459E-19 |
| CCB | rs116936375 | 10 | 18737135 | A | G | 0.0405 | 0.574 | 0.08 | 1.395E-12 |
| CCB | rs12778700 | 10 | 18385490 | C | T | 0.3323 | 0.197 | 0.03 | 2.242E-09 |
| CCB | rs1539680 | 10 | 18502889 | C | G | 0.7929 | 0.326 | 0.04 | 3.366E-18 |
| CCB | rs1891392 | 10 | 18336421 | C | T | 0.7129 | 0.228 | 0.03 | 9.02E-12 |
| CCB | rs1779246 | 10 | 18506911 | A | G | 0.7957 | 0.296 | 0.04 | 3.469E-15 |
| CCB | rs1277754 | 10 | 18523329 | G | A | 0.7174 | 0.227 | 0.03 | 2.055E-11 |
| CCB | rs3821843 | 3 | 53558012 | G | A | 0.6808 | 0.337 | 0.03 | 6.558E-24 |
| CCB | rs9311502 | 3 | 53560321 | T | C | 0.2391 | 0.246 | 0.04 | 3.873E-12 |
| CCB | rs114718455 | 3 | 53464055 | A | G | 0.0336 | 0.51 | 0.09 | 1.718E-08 |
| CCB | rs1547950 | 3 | 53568283 | T | C | 0.4623 | 0.215 | 0.03 | 2.327E-12 |
| CCB | rs114987861 | 3 | 53605712 | G | A | 0.0284 | 0.529 | 0.1 | 3.36E-08 |
| CCB | rs3774475 | 3 | 53650483 | T | A | 0.4179 | 0.185 | 0.03 | 1.602E-09 |
| CCB | rs2633731 | 3 | 53738424 | T | C | 0.6038 | 0.196 | 0.03 | 2.207E-10 |
| CCB | rs7340705 | 3 | 53734443 | T | C | 0.3268 | 0.243 | 0.03 | 4.873E-14 |
| CCB | rs11012760 | 10 | 18415289 | C | G | 0.184 | 0.285 | 0.04 | 3.341E-13 |
| CCB | rs35241357 | 10 | 18686265 | A | G | 0.3522 | 0.308 | 0.03 | 2.641E-22 |
| CCB | rs4748476 | 10 | 18792875 | C | T | 0.7771 | 0.217 | 0.04 | 2.894E-09 |
| CCB | rs7478172 | 10 | 18410168 | T | C | 0.1515 | 0.256 | 0.04 | 1.369E-09 |
| CCB | rs17604757 | 10 | 18442940 | A | G | 0.0675 | 0.502 | 0.06 | 1.122E-16 |
| CCB | rs7923191 | 10 | 18727901 | A | G | 0.2082 | 0.369 | 0.04 | 1.095E-22 |
| CCB | rs4748472 | 10 | 18776197 | C | T | 0.6558 | 0.316 | 0.03 | 4.041E-23 |
| CCB | rs12416030 | 10 | 18789075 | T | C | 0.2031 | 0.209 | 0.04 | 4.322E-08 |
| CCB | rs2488152 | 10 | 18355456 | A | G | 0.1045 | 0.377 | 0.05 | 2.543E-14 |
| CCB | rs11012811 | 10 | 18438456 | G | T | 0.31 | 0.31 | 0.03 | 2.308E-21 |
| CCB | rs1888693 | 10 | 18440444 | G | A | 0.3449 | 0.386 | 0.03 | 4.691E-34 |
| CCB | rs61278674 | 10 | 18481737 | A | G | 0.0938 | 0.33 | 0.05 | 1.032E-09 |
| CCB | rs12570727 | 10 | 18425519 | G | A | 0.3972 | 0.349 | 0.03 | 1.438E-29 |
| CCB | rs12358164 | 10 | 18450043 | A | C | 0.3242 | 0.26 | 0.03 | 6.883E-16 |
| CCB | rs4748463 | 10 | 18712250 | G | A | 0.5322 | 0.386 | 0.03 | 1.786E-37 |
| CCB | rs74593582 | 10 | 18374059 | T | C | 0.0154 | 0.758 | 0.13 | 1.914E-08 |
| CCB | rs7908738 | 10 | 18427746 | G | C | 0.1732 | 0.253 | 0.04 | 3.176E-10 |
| CCB | rs16916922 | 10 | 18467744 | A | T | 0.1415 | 0.366 | 0.04 | 2.86E-17 |
| CCB | rs12780039 | 10 | 18678987 | G | C | 0.121 | 0.285 | 0.05 | 1.263E-09 |
| CCB | rs76719841 | 10 | 18380347 | T | C | 0.0411 | 0.482 | 0.08 | 6.344E-10 |
| CCB | rs10741039 | 10 | 18659816 | C | A | 0.5239 | 0.172 | 0.03 | 1.218E-08 |
| CCB | rs11591541 | 10 | 18421314 | A | G | 0.1614 | 0.423 | 0.04 | 1.049E-24 |
| CCB | rs1998822 | 10 | 18755664 | A | G | 0.2766 | 0.196 | 0.03 | 1.145E-08 |
| CCB | rs2239046 | 12 | 2434419 | G | A | 0.6817 | 0.208 | 0.03 | 9.576E-11 |
| CCB | rs150857355 | 12 | 49209340 | G | C | 0.0217 | 0.941 | 0.11 | 5.198E-17 |
| CCB | rs17123362 | 12 | 49255964 | G | A | 0.0586 | 0.407 | 0.07 | 1.407E-09 |
| CCB | rs714277 | 12 | 2514270 | C | T | 0.2834 | 0.199 | 0.03 | 2.378E-09 |
| CCB | rs138650910 | 12 | 49305787 | A | T | 0.035 | 0.599 | 0.09 | 3.531E-12 |
| Thiazides | rs6860245 | 5 | 127367998 | C | G | 0.2435 | 0.211 | 0.04 | 2.098E-09 |
| Thiazides | rs17676396 | 5 | 127616544 | C | T | 0.2948 | 0.21 | 0.03 | 2.217E-10 |
| Thiazides | rs72794392 | 5 | 127490061 | A | T | 0.2107 | 0.241 | 0.04 | 7.031E-11 |
| Thiazides | rs17676242 | 5 | 127589648 | G | A | 0.0915 | 0.416 | 0.05 | 6.622E-15 |
| Thiazides | rs36029774 | 15 | 48693034 | T | C | 0.1042 | 0.376 | 0.05 | 1.231E-13 |

ACEi, angiotensin-converting enzyme inhibitors; BB, β-blockers; CCB, calcium channel blockers; SNP, single nucleotide polymorphism; Chr: chromosome; Pos, position (hg19); EA, effect allele; OA, other allele; EAF, effect allele frequency; SE, standard error.

**Supplementary Table 6. SNPs related to diastolic BP in genes that are the target of** **antihypertensive drugs in one-sample Mendelian randomization analysis.**

| **Drug** | **SNP** | **Chr** | **Pos** | **EA** | **OA** | **EAF** | **Beta** | **SE** | **P value** |
| --- | --- | --- | --- | --- | --- | --- | --- | --- | --- |
| ACEi | rs28656895 | 17 | 61456256 | T | C | 0.2337 | 0.1133 | 0.0206 | 3.944E-08 |
| ACEi | rs4295 | 17 | 61556298 | G | C | 0.6202 | 0.1802 | 0.018 | 1.7061E-23 |
| BB | rs73280613 | 5 | 148301076 | A | G | 0.0557 | 0.2156 | 0.0383 | 1.753E-08 |
| BB | rs41313071 | 7 | 150642752 | A | C | 0.0462 | 0.3053 | 0.0429 | 1.146E-12 |
| BB | rs891511 | 7 | 150704843 | A | G | 0.3322 | 0.2634 | 0.0191 | 2.1101E-43 |
| BB | rs740956 | 7 | 150598440 | C | T | 0.4333 | 0.1507 | 0.0175 | 7.2778E-18 |
| BB | rs34723411 | 7 | 150608794 | A | G | 0.0307 | 0.2888 | 0.053 | 4.9851E-08 |
| BB | rs2888691 | 7 | 150629639 | A | G | 0.1526 | 0.1935 | 0.0244 | 2.3281E-15 |
| BB | rs3807375 | 7 | 150667210 | T | C | 0.3626 | 0.146 | 0.0182 | 8.8308E-16 |
| BB | rs2853792 | 7 | 150699877 | A | G | 0.6067 | 0.154 | 0.0182 | 3.1412E-17 |
| BB | rs3800787 | 7 | 150713636 | C | G | 0.3766 | 0.13 | 0.0187 | 3.1783E-12 |
| BB | rs79684285 | 7 | 150719035 | T | C | 0.0316 | 0.3092 | 0.0517 | 2.21E-09 |
| BB | rs3763486 | 7 | 150723265 | C | T | 0.1828 | 0.155 | 0.023 | 1.5322E-11 |
| BB | rs10234348 | 7 | 150588863 | C | T | 0.2488 | 0.1632 | 0.0201 | 4.4648E-16 |
| BB | rs2373962 | 7 | 150680977 | C | G | 0.6277 | 0.1453 | 0.018 | 7.9653E-16 |
| BB | rs753482 | 7 | 150706383 | A | C | 0.7891 | 0.1929 | 0.0218 | 1.0671E-18 |
| BB | rs79850079 | 10 | 115790006 | A | G | 0.0319 | 0.3244 | 0.0517 | 3.382E-10 |
| BB | rs68122733 | 10 | 115831533 | G | A | 0.1717 | 0.2104 | 0.0232 | 1.344E-19 |
| BB | rs180898 | 10 | 115765397 | C | A | 0.0658 | 0.3143 | 0.0372 | 2.7631E-17 |
| BB | rs17875422 | 10 | 115798092 | G | A | 0.0374 | 0.3514 | 0.0478 | 1.9262E-13 |
| BB | rs855715 | 10 | 115823524 | T | G | 0.1202 | 0.3235 | 0.0276 | 1.0691E-31 |
| BB | rs1800888 | 5 | 148206885 | C | T | 0.0143 | 0.4644 | 0.0764 | 1.22E-09 |
| BB | rs76884999 | 7 | 150732649 | C | T | 0.0256 | 0.3827 | 0.0566 | 1.3539E-11 |
| BB | rs45453396 | 7 | 150556530 | G | A | 0.3445 | 0.1493 | 0.0184 | 5.5335E-16 |
| BB | rs79043825 | 7 | 150618901 | C | A | 0.0395 | 0.2934 | 0.0462 | 2.134E-10 |
| BB | rs138595199 | 7 | 150689016 | G | A | 0.0151 | 0.5699 | 0.0807 | 1.6181E-12 |
| BB | rs3918226 | 7 | 150690176 | C | T | 0.0813 | 0.6117 | 0.0329 | 5.3064E-77 |
| BB | rs12540183 | 7 | 150664141 | C | T | 0.3852 | 0.1723 | 0.0179 | 6.7205E-22 |
| BB | rs117564322 | 7 | 150684021 | G | A | 0.0312 | 0.6563 | 0.0528 | 1.7701E-35 |
| BB | rs2782978 | 10 | 115780121 | C | T | 0.3975 | 0.1058 | 0.018 | 3.893E-09 |
| BB | rs2484294 | 10 | 115792062 | G | A | 0.7327 | 0.3165 | 0.0196 | 1.1711E-58 |
| BB | rs1411407 | 10 | 115747786 | T | C | 0.5243 | 0.1109 | 0.0176 | 3.348E-10 |
| BB | rs11196597 | 10 | 115788094 | G | A | 0.1329 | 0.1817 | 0.0262 | 3.8168E-12 |
| BB | rs2429511 | 10 | 115801253 | T | C | 0.48 | 0.2425 | 0.0174 | 2.3459E-44 |
| BB | rs11196553 | 10 | 115710997 | C | T | 0.0448 | 0.416 | 0.0425 | 1.288E-22 |
| BB | rs180940 | 10 | 115722411 | A | G | 0.6734 | 0.1981 | 0.0185 | 1.0819E-26 |
| BB | rs143854972 | 10 | 115843445 | G | A | 0.058 | 0.2937 | 0.038 | 1.146E-14 |
| BB | rs17875473 | 10 | 115800294 | C | T | 0.0865 | 0.2459 | 0.0317 | 8.6437E-15 |
| BB | rs11196627 | 10 | 115845762 | T | C | 0.393 | 0.1237 | 0.0182 | 1.073E-11 |
| BB | rs17091184 | 10 | 115748088 | G | T | 0.0464 | 0.2324 | 0.0423 | 3.956E-08 |
| BB | rs2782980 | 10 | 115781527 | T | C | 0.7168 | 0.298 | 0.0194 | 3.2501E-53 |
| BB | rs11196625 | 10 | 115843990 | G | A | 0.1936 | 0.1717 | 0.0226 | 2.8307E-14 |
| CCB | rs113210396 | 3 | 53612327 | T | G | 0.0455 | 0.2539 | 0.0439 | 7.0889E-09 |
| CCB | rs62250937 | 3 | 53870318 | C | T | 0.0893 | 0.1861 | 0.032 | 6.353E-09 |
| CCB | rs12778700 | 10 | 18385490 | C | T | 0.333 | 0.1355 | 0.0189 | 6.5826E-13 |
| CCB | rs112133583 | 10 | 18695681 | T | C | 0.0303 | 0.3277 | 0.0552 | 2.879E-09 |
| CCB | rs10828662 | 10 | 18703097 | T | C | 0.5586 | 0.1678 | 0.0174 | 6.298E-22 |
| CCB | rs67214975 | 10 | 18727251 | A | C | 0.4567 | 0.2242 | 0.0176 | 2.6382E-37 |
| CCB | rs7087598 | 10 | 18468505 | T | C | 0.3005 | 0.1163 | 0.0195 | 2.488E-09 |
| CCB | rs4748444 | 10 | 18494482 | C | T | 0.3367 | 0.1284 | 0.0187 | 7.1664E-12 |
| CCB | rs75699707 | 10 | 18359294 | A | G | 0.0266 | 0.3514 | 0.0583 | 1.625E-09 |
| CCB | rs7076486 | 10 | 18363141 | C | G | 0.2958 | 0.1055 | 0.0192 | 3.796E-08 |
| CCB | rs10828399 | 10 | 18553968 | A | G | 0.5207 | 0.1076 | 0.0173 | 5.087E-10 |
| CCB | rs12258967 | 10 | 18727959 | G | C | 0.2958 | 0.354 | 0.0193 | 3.2719E-75 |
| CCB | rs116936375 | 10 | 18737135 | A | G | 0.0403 | 0.3088 | 0.0464 | 2.8197E-11 |
| CCB | rs10828749 | 10 | 18756881 | A | G | 0.412 | 0.2193 | 0.0177 | 3.7454E-35 |
| CCB | rs2497793 | 10 | 18342172 | T | C | 0.7675 | 0.121 | 0.0206 | 4.382E-09 |
| CCB | rs7917532 | 10 | 18373902 | C | T | 0.5343 | 0.1232 | 0.0174 | 1.473E-12 |
| CCB | rs1779240 | 10 | 18476313 | A | G | 0.764 | 0.2145 | 0.0203 | 4.7863E-26 |
| CCB | rs2250996 | 10 | 18517047 | G | A | 0.6861 | 0.1499 | 0.0186 | 8.0779E-16 |
| CCB | rs79666207 | 10 | 18583840 | C | T | 0.0177 | 0.3968 | 0.0694 | 1.097E-08 |
| CCB | rs11013938 | 10 | 18669271 | C | G | 0.2561 | 0.195 | 0.0201 | 2.7791E-22 |
| CCB | rs72786085 | 10 | 18713206 | C | G | 0.0801 | 0.3114 | 0.0338 | 3.2292E-20 |
| CCB | rs10828689 | 10 | 18721957 | C | G | 0.4436 | 0.2374 | 0.0174 | 3.7043E-42 |
| CCB | rs7917206 | 10 | 18727919 | G | C | 0.5964 | 0.1111 | 0.0179 | 4.909E-10 |
| CCB | rs72786098 | 10 | 18729855 | A | G | 0.0325 | 0.3338 | 0.0502 | 3.0451E-11 |
| CCB | rs17610275 | 10 | 18621630 | G | T | 0.0732 | 0.2887 | 0.0351 | 2.0701E-16 |
| CCB | rs982003 | 10 | 18707296 | T | C | 0.7566 | 0.1242 | 0.0201 | 7.131E-10 |
| CCB | rs1539680 | 10 | 18502889 | C | G | 0.7938 | 0.1512 | 0.0215 | 2.03E-12 |
| CCB | rs10828452 | 10 | 18592450 | T | A | 0.2076 | 0.1854 | 0.0222 | 6.5178E-17 |
| CCB | rs10828542 | 10 | 18627285 | G | A | 0.3866 | 0.0996 | 0.0178 | 2.341E-08 |
| CCB | rs11014170 | 10 | 18710991 | A | G | 0.0206 | 0.3648 | 0.0657 | 2.815E-08 |
| CCB | rs2283274 | 12 | 2184466 | C | G | 0.1801 | 0.1333 | 0.0231 | 8.08E-09 |
| CCB | rs3821843 | 3 | 53558012 | G | A | 0.6805 | 0.1706 | 0.0192 | 5.3939E-19 |
| CCB | rs9311502 | 3 | 53560321 | T | C | 0.2392 | 0.1225 | 0.0203 | 1.704E-09 |
| CCB | rs3774475 | 3 | 53650483 | T | A | 0.4184 | 0.1195 | 0.0176 | 1.112E-11 |
| CCB | rs2633731 | 3 | 53738424 | T | C | 0.6034 | 0.1409 | 0.0178 | 2.1028E-15 |
| CCB | rs6776071 | 3 | 53656542 | C | A | 0.3706 | 0.1153 | 0.0181 | 1.719E-10 |
| CCB | rs74857353 | 3 | 53731094 | G | A | 0.0506 | 0.2515 | 0.0416 | 1.553E-09 |
| CCB | rs12494849 | 3 | 50524558 | C | G | 0.1454 | 0.1659 | 0.0248 | 2.3469E-11 |
| CCB | rs1547950 | 3 | 53568283 | T | C | 0.4625 | 0.1132 | 0.0176 | 1.152E-10 |
| CCB | rs11720002 | 3 | 53709642 | T | C | 0.276 | 0.1494 | 0.0193 | 1.1189E-14 |
| CCB | rs2680663 | 3 | 53735299 | A | G | 0.325 | 0.1779 | 0.0185 | 7.3333E-22 |
| CCB | rs75940076 | 3 | 53717956 | G | A | 0.1355 | 0.1767 | 0.0258 | 7.1664E-12 |
| CCB | rs12358149 | 10 | 18413731 | G | A | 0.0336 | 0.2752 | 0.0494 | 2.602E-08 |
| CCB | rs10828263 | 10 | 18421204 | G | A | 0.2361 | 0.1171 | 0.0205 | 1.141E-08 |
| CCB | rs16916914 | 10 | 18457722 | T | C | 0.0366 | 0.3601 | 0.0464 | 8.0427E-15 |
| CCB | rs10741039 | 10 | 18659816 | C | A | 0.5232 | 0.099 | 0.0173 | 1.029E-08 |
| CCB | rs7069923 | 10 | 18730368 | C | T | 0.3373 | 0.1609 | 0.0184 | 1.8802E-18 |
| CCB | rs4628581 | 10 | 18799890 | A | C | 0.4544 | 0.1019 | 0.0174 | 5.018E-09 |
| CCB | rs10764316 | 10 | 18409507 | C | G | 0.2554 | 0.1539 | 0.0199 | 1.1061E-14 |
| CCB | rs56276305 | 10 | 18450970 | G | A | 0.0937 | 0.1697 | 0.0297 | 1.131E-08 |
| CCB | rs2488159 | 10 | 18370991 | G | A | 0.1042 | 0.1745 | 0.0284 | 7.7121E-10 |
| CCB | rs11012829 | 10 | 18444535 | G | A | 0.1591 | 0.1776 | 0.0238 | 7.925E-14 |
| CCB | rs7096168 | 10 | 18693174 | T | A | 0.1996 | 0.1267 | 0.0217 | 5.5E-09 |
| CCB | rs7895467 | 10 | 18762948 | G | A | 0.3328 | 0.14 | 0.0184 | 2.4769E-14 |
| CCB | rs4748475 | 10 | 18790980 | G | A | 0.4937 | 0.1315 | 0.0173 | 3.3389E-14 |
| CCB | rs113390901 | 10 | 18441739 | A | T | 0.0689 | 0.1951 | 0.0347 | 1.871E-08 |
| CCB | rs10828295 | 10 | 18466094 | A | G | 0.2525 | 0.1949 | 0.02 | 2.2782E-22 |
| CCB | rs11012932 | 10 | 18480227 | A | G | 0.1699 | 0.187 | 0.0231 | 6.5826E-16 |
| CCB | rs61278674 | 10 | 18481737 | A | G | 0.0945 | 0.2193 | 0.0309 | 1.188E-12 |
| CCB | rs7908738 | 10 | 18427746 | G | C | 0.1733 | 0.1586 | 0.023 | 5.5667E-12 |
| CCB | rs34606998 | 10 | 18430855 | C | T | 0.2398 | 0.1556 | 0.0204 | 2.6847E-14 |
| CCB | rs4748463 | 10 | 18712250 | G | A | 0.5325 | 0.211 | 0.0173 | 3.2817E-34 |
| CCB | rs4748472 | 10 | 18776197 | C | T | 0.6554 | 0.1639 | 0.0183 | 3.2181E-19 |
| CCB | rs12773254 | 10 | 18891168 | G | C | 0.157 | 0.1413 | 0.0239 | 3.283E-09 |
| CCB | rs76304369 | 10 | 18359213 | C | T | 0.0128 | 0.4608 | 0.0788 | 4.918E-09 |
| CCB | rs10740993 | 10 | 18442482 | C | T | 0.4385 | 0.2185 | 0.0174 | 3.8291E-36 |
| CCB | rs11012877 | 10 | 18466378 | G | A | 0.0926 | 0.288 | 0.0302 | 1.582E-21 |
| CCB | rs7923191 | 10 | 18727901 | A | G | 0.2079 | 0.212 | 0.0216 | 9.8992E-23 |
| CCB | rs12416030 | 10 | 18789075 | T | C | 0.2034 | 0.155 | 0.0219 | 1.3329E-12 |
| CCB | rs55935819 | 12 | 2521579 | G | A | 0.3636 | 0.1271 | 0.0181 | 1.9579E-12 |
| CCB | rs150857355 | 12 | 49209340 | G | C | 0.0217 | 0.4102 | 0.0643 | 1.789E-10 |
| Thiazides | rs17676396 | 5 | 127616544 | C | T | 0.2931 | 0.124 | 0.0191 | 7.7643E-11 |
| Thiazides | rs12919839 | 16 | 56859216 | T | C | 0.2841 | 0.1098 | 0.0192 | 1.044E-08 |
| Thiazides | rs17676242 | 5 | 127589648 | G | A | 0.0921 | 0.1851 | 0.0305 | 1.325E-09 |

ACEi, angiotensin-converting enzyme inhibitors; BB, β-blockers; CCB, calcium channel blockers; SNP, single nucleotide polymorphism; Chr: chromosome; Pos, position (hg19); EA, effect allele; OA, other allele; EAF, effect allele frequency; SE, standard error.

**Supplementary Table 7. SMR estimates of antihypertension drug target genes on FI.**

| **Probe ID** | **Gene** | **Top cis-eQTL** | **Chromosome** | **Position** | **A1** | **A2** | **EAF** | **SMR** | | | **HEIDI test** | | **Tissue** |
| --- | --- | --- | --- | --- | --- | --- | --- | --- | --- | --- | --- | --- | --- |
|  |  |  |  |  |  |  |  | **Beta** | **SE** | **P value** | **P value** | **No. of cis-eQTL** |  |
| **ENSG00000055118** | **KCNH2** | **rs887586** | **7** | **1.51E+08** | **G** | **C** | **0.607362** | **0.022753** | **0.006274** | **2.87E-04^**^** | **5.69E-01** | **20** | **Artery Aorta** |
| **ENSG00000055118** | **KCNH2** | **rs882156** | **7** | **1.51E+08** | **C** | **T** | **0.607362** | **0.051205** | **0.014966** | **6.23E-04^**^** | **3.26E-01** | **20** | **Artery Tibial** |
| ENSG00000074803 | SLC12A1 | rs74012001 | 15 | 48605208 | T | A | 0.171779 | 0.0087535 | 0.0035505 | 1.37E-02**^*^** | 8.67E-02 | 20 | Whole Blood |
| ENSG00000151067 | CACNA1C | rs886898 | 12 | 2481936 | C | T | 0.856851 | 0.0125419 | 0.0054413 | 2.12E-02**^*^** | 5.99E-01 | 10 | Brain Cerebellum |
| ENSG00000151067 | CACNA1C | rs886898 | 12 | 2481936 | C | T | 0.856851 | 0.0120617 | 0.0052595 | 2.18E-02**^*^** | 4.30E-01 | 11 | Brain Cerebellar Hemisphere |
| ENSG00000132170 | PPARG | rs73025230 | 3 | 12379911 | C | A | 0.0961145 | 0.0071775 | 0.0040881 | 7.91E-02 | 2.70E-01 | 20 | Brain Cerebellum |
| ENSG00000132170 | PPARG | rs73025230 | 3 | 12379911 | C | A | 0.0961145 | 0.0057994 | 0.0033063 | 7.94E-02 | 3.09E-01 | 20 | Brain Cerebellar Hemisphere |
| ENSG00000156113 | KCNMA1 | rs2619621 | 10 | 79385070 | T | C | 0.522495 | 0.0266038 | 0.0157886 | 9.20E-02 | 2.41E-01 | 14 | Brain Putamen basal ganglia |
| ENSG00000055118 | KCNH2 | rs3807375 | 7 | 150667210 | T | C | 0.349693 | 0.0206542 | 0.0125677 | 1.00E-01 | 1.05E-01 | 20 | Nerve Tibial |
| ENSG00000169252 | ADRB2 | rs2400645 | 5 | 148120006 | C | T | 0.485685 | 0.0153505 | 0.009462 | 1.05E-01 | 5.84E-01 | 6 | Heart Atrial Appendage |
| ENSG00000156113 | KCNMA1 | rs2619608 | 10 | 79336052 | C | G | 0.48773 | 0.0189957 | 0.0124773 | 1.28E-01 | 3.49E-01 | 20 | Artery Aorta |
| ENSG00000156113 | KCNMA1 | rs816867 | 10 | 79312437 | C | T | 0.51227 | 0.008541 | 0.0058896 | 1.47E-01 | 3.10E-01 | 20 | Whole Blood |
| ENSG00000156113 | KCNMA1 | rs816868 | 10 | 79312114 | T | C | 0.51227 | 0.0121453 | 0.0084228 | 1.49E-01 | 3.72E-01 | 20 | Nerve Tibial |
| ENSG00000156113 | KCNMA1 | rs816868 | 10 | 79312114 | T | C | 0.51227 | 0.0121095 | 0.0084206 | 1.50E-01 | 5.49E-01 | 20 | Adipose Visceral Omentum |
| ENSG00000156113 | KCNMA1 | rs2176283 | 10 | 79287128 | G | T | 0.506135 | 0.0112569 | 0.008215 | 1.71E-01 | 3.26E-01 | 20 | Heart Left Ventricle |
| ENSG00000156113 | KCNMA1 | rs2176283 | 10 | 79287128 | G | T | 0.506135 | 0.0114152 | 0.0083679 | 1.73E-01 | 5.42E-01 | 20 | Heart Atrial Appendage |
| ENSG00000055118 | KCNH2 | rs740955 | 7 | 150598479 | C | T | 0.449898 | 0.0130343 | 0.0095962 | 1.74E-01 | 9.40E-01 | 20 | Adrenal Gland |
| ENSG00000081248 | CACNA1S | rs7513829 | 1 | 201079590 | A | C | 0.393661 | -0.0192056 | 0.0142076 | 1.76E-01 | 9.22E-01 | 20 | Artery Tibial |
| ENSG00000167535 | CACNB3 | rs34014405 | 12 | 49211913 | T | C | 0.179959 | 0.0181985 | 0.0140617 | 1.96E-01 | 3.71E-02 | 20 | Whole Blood |
| ENSG00000167535 | CACNB3 | rs34014405 | 12 | 49211913 | T | C | 0.179959 | -0.0129469 | 0.0100512 | 1.98E-01 | 3.77E-02 | 20 | Brain Putamen basal ganglia |
| ENSG00000167535 | CACNB3 | rs34014405 | 12 | 49211913 | T | C | 0.179959 | -0.0174908 | 0.0136404 | 2.00E-01 | 7.97E-02 | 17 | Brain Caudate basal ganglia |
| ENSG00000167535 | CACNB3 | rs34014405 | 12 | 49211913 | T | C | 0.179959 | -0.0120137 | 0.0093794 | 2.00E-01 | 1.22E-01 | 19 | Brain Cerebellum |
| ENSG00000167535 | CACNB3 | rs12369114 | 12 | 49212806 | T | C | 0.179959 | -0.0127811 | 0.0101057 | 2.06E-01 | 1.11E-01 | 20 | Brain Nucleus accumbens basal ganglia |
| ENSG00000156113 | KCNMA1 | rs2673406 | 10 | 79391584 | A | T | 0.52454 | 0.0240078 | 0.0190115 | 2.07E-01 | 5.00E-01 | 9 | Brain Caudate basal ganglia |
| ENSG00000167535 | CACNB3 | rs12369114 | 12 | 49212806 | T | C | 0.179959 | -0.0125882 | 0.010005 | 2.08E-01 | 2.64E-01 | 20 | Brain Cerebellar Hemisphere |
| ENSG00000151067 | CACNA1C | rs2283275 | 12 | 2184560 | T | C | 0.757669 | -0.0269153 | 0.0215131 | 2.11E-01 | 2.73E-01 | 6 | Artery Tibial |
| ENSG00000144891 | AGTR1 | rs3772633 | 3 | 148418168 | C | T | 0.156442 | -0.0116333 | 0.0095164 | 2.22E-01 | 8.90E-01 | 9 | Heart Atrial Appendage |
| ENSG00000196557 | CACNA1H | rs12926678 | 16 | 1187931 | T | C | 0.406953 | -0.0093902 | 0.0079305 | 2.36E-01 | 5.59E-01 | 13 | Adrenal Gland |
| ENSG00000043591 | ADRB1 | rs4918889 | 10 | 115830718 | G | C | 0.166667 | -0.0140134 | 0.0122638 | 2.53E-01 | 8.01E-01 | 16 | Whole Blood |
| ENSG00000167535 | CACNB3 | rs150857355 | 12 | 49209340 | C | G | 0.0184049 | 0.0135634 | 0.0126351 | 2.83E-01 | 1.38E-01 | 20 | Artery Tibial |
| ENSG00000167535 | CACNB3 | rs150857355 | 12 | 49209340 | C | G | 0.0184049 | 0.0120161 | 0.0112051 | 2.84E-01 | 2.22E-01 | 20 | Nerve Tibial |
| ENSG00000167535 | CACNB3 | rs150857355 | 12 | 49209340 | C | G | 0.0184049 | 0.0149051 | 0.0139087 | 2.84E-01 | 3.47E-01 | 20 | Artery Aorta |
| ENSG00000167535 | CACNB3 | rs150857355 | 12 | 49209340 | C | G | 0.0184049 | 0.0185452 | 0.0173682 | 2.86E-01 | 4.83E-01 | 9 | Adipose Visceral Omentum |
| ENSG00000167535 | CACNB3 | rs150857355 | 12 | 49209340 | C | G | 0.0184049 | 0.0196507 | 0.0184391 | 2.87E-01 | 5.78E-01 | 8 | Heart Atrial Appendage |
| ENSG00000167535 | CACNB3 | rs150857355 | 12 | 49209340 | C | G | 0.0184049 | 0.0192601 | 0.0180978 | 2.87E-01 | 6.54E-01 | 6 | Artery Aorta |
| ENSG00000165995 | CACNB2 | rs10764318 | 10 | 18415963 | A | G | 0.123722 | 0.0196055 | 0.0189347 | 3.00E-01 | 5.16E-01 | 20 | Brain Nucleus accumbens basal ganglia |
| ENSG00000064651 | SLC12A2 | rs10478787 | 5 | 127294105 | A | C | 0.575665 | 0.0082509 | 0.0080894 | 3.08E-01 | 7.01E-02 | 15 | Brain Cerebellar Hemisphere |
| ENSG00000064651 | SLC12A2 | rs10478787 | 5 | 127294105 | A | C | 0.575665 | 0.0095081 | 0.009335 | 3.08E-01 | 5.65E-02 | 14 | Brain Cerebellum |
| ENSG00000055118 | KCNH2 | rs11771808 | 7 | 150675868 | G | A | 0.580777 | -0.0077604 | 0.0082872 | 3.49E-01 | 3.59E-02 | 20 | Brain Cerebellum |
| ENSG00000055118 | KCNH2 | rs11771808 | 7 | 150675868 | G | A | 0.580777 | -0.0083506 | 0.0089598 | 3.51E-01 | 8.00E-02 | 20 | Brain Cerebellar Hemisphere |
| ENSG00000165995 | CACNB2 | rs10828906 | 10 | 18841795 | T | C | 0.754601 | 0.0076152 | 0.0086105 | 3.76E-01 | 3.36E-01 | 20 | Brain Cerebellum |
| ENSG00000159640 | ACE | rs4291 | 17 | 61554194 | A | T | 0.630879 | 0.0070482 | 0.0086482 | 4.15E-01 | 1.30E-01 | 4 | Brain Frontal Cortex BA9 |
| ENSG00000120907 | ADRA1A | rs62494676 | 8 | 26596238 | A | G | 0.195297 | -0.0104119 | 0.0133595 | 4.36E-01 | 5.17E-01 | 20 | Artery Tibial |
| ENSG00000159640 | ACE | rs4292 | 17 | 61554341 | T | C | 0.634969 | 0.0135167 | 0.017819 | 4.48E-01 | 7.95E-02 | 13 | Nerve Tibial |
| ENSG00000064651 | SLC12A2 | rs4276454 | 5 | 127073285 | A | G | 0.399796 | -0.0127712 | 0.0179701 | 4.77E-01 | 5.56E-01 | 20 | Artery Tibial |
| ENSG00000159640 | ACE | rs4308 | 17 | 61559625 | G | A | 0.635992 | 0.0142395 | 0.02211 | 5.20E-01 | 8.81E-02 | 17 | Artery Aorta |
| ENSG00000159640 | ACE | rs4308 | 17 | 61559625 | G | A | 0.635992 | 0.0053656 | 0.0083401 | 5.20E-01 | 1.63E-01 | 12 | Brain Cerebellum |
| ENSG00000159640 | ACE | rs4308 | 17 | 61559625 | G | A | 0.635992 | -0.0038966 | 0.0060638 | 5.20E-01 | NA | NA | Heart Left Ventricle |
| ENSG00000159640 | ACE | rs4308 | 17 | 61559625 | G | A | 0.635992 | -0.0038966 | 0.0060638 | 5.20E-01 | NA | NA | Kidney Cortex |
| ENSG00000153956 | CACNA2D1 | rs59665824 | 7 | 81844929 | C | T | 0.0460123 | 0.0089459 | 0.0158428 | 5.72E-01 | 4.96E-01 | 20 | Artery Aorta |
| ENSG00000132170 | PPARG | rs2920502 | 3 | 12329195 | C | G | 0.317996 | -0.0060265 | 0.0145004 | 6.78E-01 | 3.56E-02 | 18 | Heart Atrial Appendage |
| ENSG00000144891 | AGTR1 | rs275665 | 3 | 148390863 | A | G | 0.154397 | -0.0036134 | 0.0176719 | 8.38E-01 | 9.80E-01 | 20 | Nerve Tibial |
| ENSG00000120907 | ADRA1A | rs1079078 | 8 | 26698047 | C | A | 0.194274 | -0.0014228 | 0.0072927 | 8.45E-01 | 2.12E-01 | 20 | Artery Aorta |
| ENSG00000120907 | ADRA1A | rs1079078 | 8 | 26698047 | C | A | 0.194274 | -0.0011252 | 0.0057674 | 8.45E-01 | 5.86E-01 | 20 | Adipose Visceral Omentum |
| ENSG00000144891 | AGTR1 | rs275691 | 3 | 148343434 | A | T | 0.602249 | 0.0028279 | 0.0160288 | 8.60E-01 | 3.61E-01 | 7 | Artery Tibial |
| ENSG00000144891 | AGTR1 | rs275677 | 3 | 148369183 | A | G | 0.191207 | 0.0010548 | 0.021097 | 9.60E-01 | 9.57E-01 | 20 | Artery Aorta |
| ENSG00000196557 | CACNA1H | rs117177120 | 16 | 1238875 | G | T | 0.0664622 | 0.0005497 | 0.0200641 | 9.78E-01 | 4.61E-02 | 7 | Adipose Visceral Omentum |
| ENSG00000196557 | CACNA1H | rs117177120 | 16 | 1238875 | G | T | 0.0664622 | 0.0005715 | 0.0208609 | 9.78E-01 | 4.62E-02 | 14 | Artery Tibial |

eQTL, expression quantitative trait locus; SMR, summary data-based Mendelian randomization; FI, frailty index; EAF, effect allele frequency; HEIDI, heterogeneity in dependent instruments.

**^**^,** statistical significance level after the correction for multiple testing.

**^*^,** suggestive significance level.

**Supplementary Table 8. Causal associations between BP and frailty status using unweighted genetic risk score as IVs.**

| **IV set** | **Pre‐frail vs. non‐frail** | | **Frail vs. non‐frail** | |
| --- | --- | --- | --- | --- |
|  | **RRR (95% CI)** | **P** | **RRR (95% CI)** | **P** |
| Sysolic BP | 0.84 (0.83,0.86) | <0.001^**^ | 0.73 (0.68,0.77) | <0.001^**^ |
| ACEI | 0.72 (0.39,1.33) | 0.296 | 0.46 (0.09,2.31) | 0.345 |
| BB | 0.78 (0.66,0.92) | 0.004^**^ | 0.62 (0.39,0.96) | 0.032^*^ |
| CCB | 0.82 (0.71,0.94) | 0.005^**^ | 0.86 (0.59,1.24) | 0.409 |
| Thiazides | 0.69 (0.50,0.97) | 0.033^*^ | 0.64 (0.26,1.54) | 0.318 |
| Diasolic BP | 0.86 (0.84,0.88) | <0.001^**^ | 0.77 (0.73,0.82) | <0.001^**^ |
| ACEI | 0.76 (0.52,1.11) | 0.151 | 0.60 (0.22,1.62) | 0.313 |
| BB | 0.83 (0.74,0.93) | 0.001^**^ | 0.68 (0.50,0.92) | 0.012^**^ |
| CCB | 0.83 (0.73,0.94) | 0.004^**^ | 0.91 (0.65,1.28) | 0.594 |
| Thiazides | 0.71 (0.51,0.98) | 0.038^*^ | 0.66 (0.28,1.56) | 0.343 |

IV, instrumental variable; RRR, relative risk ratios; CI, confidence interval; BP, blood pressure; ACEi, angiotensin-converting enzyme inhibitors; BB, β-blockers; CCB, calcium channel blockers.

**^**^,** statistical significance level after the correction for multiple testing.

**^*^,** suggestive significance level.

**Supplementary Table 9. Causal effect of life-long lowering of BP through different antihypertensive drug classes on frailty status indicated by the constructed IVs (r2<0.1).**

| **IV set** | **Pre‐frail vs. non‐frail** | | **Frail vs. non‐frail** | |
| --- | --- | --- | --- | --- |
|  | **RRR (95% CI)** | **P** | **RRR (95% CI)** | **P** |
| Sysolic BP |  |  |  |  |
| ACEI | 0.72 (0.39,1.33) | 0.296 | 0.46 (0.09,2.31) | 0.345 |
| BB | 0.76 (0.65,0.90) | 0.001^**^ | 0.62 (0.40,0.97) | 0.034^*^ |
| CCB | 0.83 (0.72,0.94) | 0.005^**^ | 0.87 (0.61,1.24) | 0.447 |
| Thiazides | 0.74 (0.53,1.03) | 0.072 | 0.61 (0.25,1.49) | 0.279 |
| Diasolic BP |  |  |  |  |
| ACEI | 0.76 (0.52,1.11) | 0.151 | 0.60 (0.22,1.62) | 0.313 |
| BB | 0.83 (0.74,0.92) | 0.001^**^ | 0.69 (0.52,0.92) | 0.011^**^ |
| CCB | 0.83 (0.73,0.94) | 0.004^**^ | 0.90 (0.64,1.26) | 0.530 |
| Thiazides | 0.71 (0.51,0.99) | 0.044^*^ | 0.61 (0.25,1.47) | 0.271 |

IV, instrumental variable; RRR, relative risk ratios; CI, confidence interval; BP, blood pressure; ACEi, angiotensin-converting enzyme inhibitors; BB, β-blockers; CCB, calcium channel blockers.

**^**^,** statistical significance level after the correction for multiple testing.

**^*^,** suggestive significance level.

**Supplementary Table 10. Causal effect of life-long lowering of BP through different antihypertensive drug classes on frailty status indicated by the constructed IVs (r2<0.01).**

| **IV set** | **Pre‐frail vs. non‐frail** | | **Frail vs. non‐frail** | |
| --- | --- | --- | --- | --- |
|  | **RRR (95% CI)** | **P** | **RRR (95% CI)** | **P** |
| Sysolic BP |  |  |  |  |
| ACEI | 0.72 (0.39,1.33) | 0.296 | 0.46 (0.09,2.31) | 0.345 |
| BB | **0.69 (0.56,0.85)** | **0.001^**^** | 0.59 (0.34,1.01) | 0.056 |
| CCB | **0.79 (0.69,0.89)** | **<0.001^**^** | 0.79 (0.56,1.11) | 0.171 |
| Thiazides | 0.96 (0.63,1.46) | 0.315 | 0.57 (0.19,1.71) | 0.279 |
| Diasolic BP |  |  |  |  |
| ACEI | 0.76 (0.52,1.11) | 0.151 | 0.60 (0.22,1.62) | 0.313 |
| BB | **0.83 (0.75,0.91)** | **0.001^**^** | 0.69 (0.53,0.91) | 0.008**^**^** |
| CCB | **0.81 (0.72,0.91)** | **<0.001^**^** | 0.79 (0.58,1.08) | 0.137 |
| Thiazides | 0.71 (0.51,0.99) | 0.044**^*^** | 0.61 (0.25,1.47) | 0.271 |

IV, instrumental variable; RRR, relative risk ratios; CI, confidence interval; BP, blood pressure; ACEi, angiotensin-converting enzyme inhibitors; BB, β-blockers; CCB, calcium channel blockers.

**^**^,** statistical significance level after the correction for multiple testing.

**^*^,** suggestive significance level.

**Supplementary Table 11. Causal associations between BP and frailty status among participants with missing data on less than 10 FI items.**

| **IV set** | **Pre‐frail vs. non‐frail** | | **Frail vs. non‐frail** | |
| --- | --- | --- | --- | --- |
|  | **RRR (95% CI)** | **P** | **RRR (95% CI)** | **P** |
| Sysolic BP | 0.86 (0.84,0.88) | <0.001^**^ | 0.75 (0.71,0.78) | <0.001^**^ |
| ACEI | 0.71 (0.42,1.18) | 0.185 | 0.44 (0.12,1.68) | 0.233 |
| BB | 0.78 (0.67,0.90) | 0.001^**^ | 0.64 (0.43,0.93) | 0.019^**^ |
| CCB | 0.87 (0.77,0.98) | 0.025^**^ | 0.99 (0.72,1.36) | 0.960 |
| Thiazides | 0.75 (0.55,1.02) | 0.065 | 0.85 (0.38,1.91) | 0.699 |
| Diasolic BP | 0.87 (0.86,0.89) | <0.001^**^ | 0.77 (0.74,0.81) | <0.001^**^ |
| ACEI | 0.70 (0.50,0.98) | 0.038^*^ | 0.55 (0.23,1.31) | 0.177 |
| BB | 0.82 (0.74,0.91) | <0.001^**^ | 0.67 (0.52,0.87) | 0.003^**^ |
| CCB | 0.88 (0.78,0.98) | 0.024^**^ | 0.98 (0.73,1.33) | 0.918 |
| Thiazides | 0.79 (0.56,1.10) | 0.157 | 0.92 (0.38,2.19) | 0.846 |

RRR, relative risk ratios; CI, confidence interval; BP, blood pressure; FI, frailty index; ACEi, angiotensin-converting enzyme inhibitors; BB, β-blockers; CCB, calcium channel blockers.

**^**^,** statistical significance level after the correction for multiple testing.

**^*^,** suggestive significance level.

**Supplementary Table 12. Causal associations between BP and frailty status among participants from multiple ethnic/genetic groups.**

| **IV set** | **Pre‐frail vs. non‐frail** | | **Frail vs. non‐frail** | |
| --- | --- | --- | --- | --- |
|  | **RRR (95% CI)** | **P** | **RRR (95% CI)** | **P** |
| Sysolic BP | 0.85 (0.83,0.87) | <0.001^**^ | 0.74 (0.70,0.78) | <0.001^**^ |
| ACEI | 0.80 (0.47,1.37) | 0.418 | 0.57 (0.14,2.31) | 0.434 |
| BB | 0.81 (0.69,0.94) | 0.006^**^ | 0.71 (0.48,1.06) | 0.096 |
| CCB | 0.86 (0.75,0.97) | 0.017^**^ | 0.80 (0.57,1.11) | 0.182 |
| Thiazides | 0.77 (0.56,1.05) | 0.099 | 0.60 (0.26,1.38) | 0.232 |
| Diasolic BP | 0.87 (0.85,0.88) | <0.001^**^ | 0.79 (0.75,0.82) | <0.001^**^ |
| ACEI | 0.81 (0.58,1.12) | 0.202 | 0.85 (0.36,1.99) | 0.699 |
| BB | 0.85 (0.77,0.95) | 0.003^**^ | 0.73 (0.56,0.67) | 0.028^*^ |
| CCB | 0.86 (0.77,0.97) | 0.011^**^ | 0.82 (0.61,1.11) | 0.216 |
| Thiazides | 0.71 (0.50,1.00) | 0.051 | 0.55 (0.22,1.34) | 0.185 |

RRR, relative risk ratios; CI, confidence interval; BP, blood pressure; FI, frailty index; ACEi, angiotensin-converting enzyme inhibitors; BB, β-blockers; CCB, calcium channel blockers.

**^**^,** statistical significance level after the correction for multiple testing.

**^*^,** suggestive significance level.

**Supplementary Table 13.** **Causal associations between BP and frailty status indicated by Fried frailty phenotype.**

| **IV set** | **Pre‐frail vs. non‐frail** | | **Frail vs. non‐frail** | |
| --- | --- | --- | --- | --- |
|  | **RRR (95% CI)** | **P** | **RRR (95% CI)** | **P** |
| Sysolic BP | 0.99 (0.97,1.01) | 0.381 | 0.94 (0.89,0.99) | 0.023^**^ |
| ACEI | 0.73 (0.42,1.28) | 0.272 | 0.58 (0.12,2.67) | 0.481 |
| BB | 0.88 (0.76,1.02) | 0.094 | 1.10 (0.74,1.64) | 0.648 |
| CCB | 1.00 (0.88,1.13) | 0.966 | 0.91 (0.65,1.27) | 0.564 |
| Thiazides | 0.86 (0.64,1.16) | 0.318 | 0.70 (0.30,1.59) | 0.389 |
| Diasolic BP | 0.99 (0.98,1.01) | 0.514 | 0.95 (0.90,0.99) | 0.021^**^ |
| ACEI | 0.92 (0.65,1.29) | 0.631 | 0.77 (0.30,1.99) | 0.595 |
| BB | 0.93 (0.84,1.02) | 0.140 | 1.00 (0.76,1.32) | 0.999 |
| CCB | 0.99 (0.88,1.11) | 0.907 | 0.88 (0.64,1.21) | 0.422 |
| Thiazides | 0.89 (0.64,1.23) | 0.469 | 0.94 (0.38,2.30) | 0.892 |

RRR, relative risk ratios; CI, confidence interval; BP, blood pressure; FI, frailty index; ACEi, angiotensin-converting enzyme inhibitors; BB, β-blockers; CCB, calcium channel blockers.

**^**^,** statistical significance level after the correction for multiple testing.

**^*^,** suggestive significance level.

**Supplementary Figure 1. Study design schematic for initial exclusion criteria and genetic data quality control**


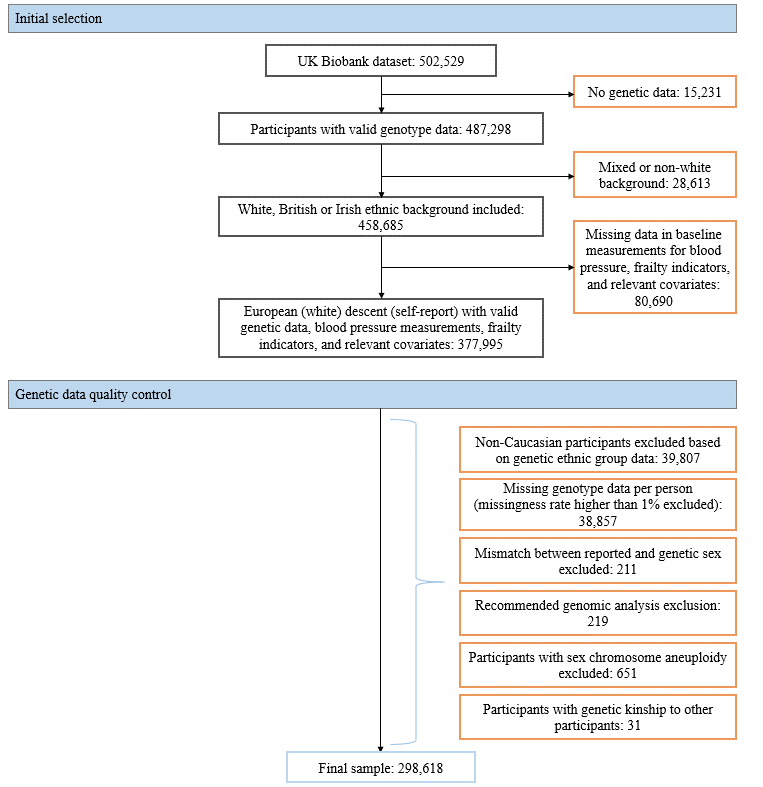


**Supplementary Figure 2. Regression line represent intercorrelations between weighted genetic risk score and BP measurements.**


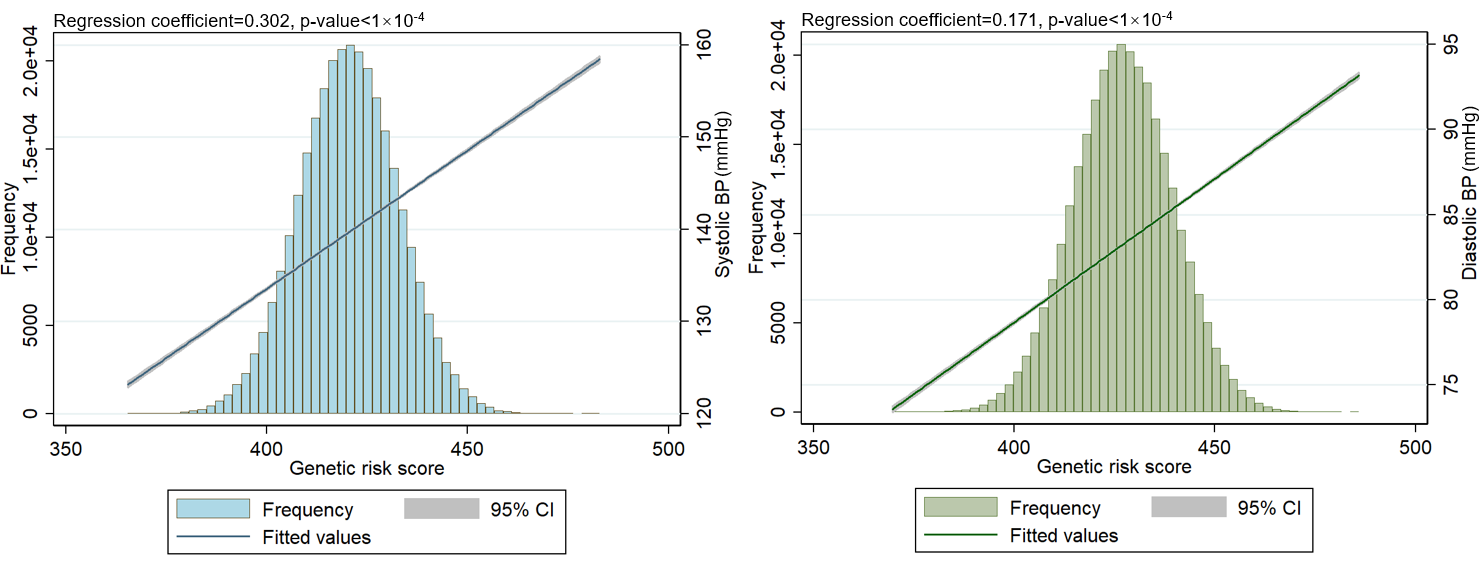


CI, confidence interval; BP, blood pressure.
